# Achieving Long‐Wavelength Electroluminescence Using Two‐Coordinate Gold(I) Complexes: Overcoming the Energy Gap Law

**DOI:** 10.1002/advs.202305745

**Published:** 2023-11-12

**Authors:** Sreenivas Avula, Byung Hak Jhun, Unhyeok Jo, Seunga Heo, Jun Yeob Lee, Youngmin You

**Affiliations:** ^1^ Department of Chemical and Biomolecular Engineering Yonsei University Seoul 03722 Republic of Korea; ^2^ School of Chemical Engineering Sungkyunkwan University Suwon Gyeonggi‐do 16419 Republic of Korea; ^3^ Division of Chemical Engineering and Materials Science Ewha Womans University Seoul 03760 Republic of Korea

**Keywords:** electroluminescence, gold complexes, radiative control, thermally activated delayed fluorescence

## Abstract

Two‐coordinate coinage metal complexes have emerged as promising emitters for highly efficient organic light‐emitting devices (OLEDs). However, achieving efficient long‐wavelength electroluminescence emission from these complexes remains as a daunting challenge. To address this challenge, molecular design strategies aimed at bolstering the photoluminescence quantum yield (*Φ*) of Au(I) complex emitters in low‐energy emission regions are investigated. By varying amido ligands, a series of two‐coordinate Au(I) complexes is developed that exhibit photoluminescence peak wavelengths over a broad range of 533−750 nm. These complexes, in particular, maintain *Φ* values up to 10% even in the near‐infrared emission region, overcoming the constraints imposed by an energy gap. Quantum chemical calculations and photophysical analyses reveal the action of radiative control, which serves to overcome the energy gap law, becomes more pronounced as the overlap between hole and electron distributions (*S*_r_(*r*)) in the excited state increases. It is further elucidated that *S*_r_(*r*) increases with the distance between the hole‐distribution centroid and the nitrogen atom in an amido ligand. Finally, multilayer OLEDs involving the Au(I) complex emitters exhibit performances beyond the borderline of the electroluminescence wavelength−external quantum efficiency space set by previous devices of coinage metal complexes.

## Graphical abstract

Synthetic research illustrates the successful use of radiative control to enhance electroluminescence efficiencies in two‐coordinate carbene‐Au(I)‐amido complexes with varied amido ligand structures, particularly within the long‐wavelength emission region that typically suffers from low emission efficiencies due to the constraints of energy‐gap laws.

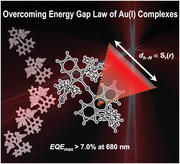

## 1 Introduction

The advances of organic light‐emitting devices (OLEDs) have been driven by the development of emitting molecules capable of harvesting all the electrogenerated excitons. In particular, organic molecules exhibiting thermally activated delayed fluorescence (TADF) enable high‐efficiency electroluminescence across a broad range of visible regions.^[^ ^1^ ^]^ However, incorporating organic TADF emitters into OLEDs presents challenges such as the moderate operational stability and a decline in electroluminescence efficiencies at high brightness, primarily due to slow exciton conversion.^[^ ^2^ ^]^ Recently, two‐coordinate complexes involving coinage metals such as Cu(I), Ag(I), and Au(I) have emerged as promising alternatives to organic TADF emitters.^[^ ^3^ ^]^ Benefiting from the strong spin−orbit coupling provided by the metals and the effective separation of frontier molecular orbitals, these metal complexes exhibit TADF through fast exciton harvesting.^[^ ^3^, ^4^ ^]^ Importantly, this exciton harvest does not compromise the quantum yield for photoluminescence (*Φ*), facilitating high‐brightness emission. As a result, coinage metal complexes are capable of uniquely combining the advantages of both organic TADF molecules and phosphorescent complexes of late transition metals, such as Ir(III) and Pt(II).

Since the first report of solution‐processed OLEDs based on a two‐coordinate Au(I) complex,^[^ ^3a^ ^]^ the research groups of Credgington and Romanov have pioneered the development of electroluminescent coinage metal complexes.^[^ ^3b–k^ ^]^ The two‐coordinate coinage metal complexes typically involve an anionic amido ligand and a neutral carbene ligand. This structure permits the ligand‐to‐ligand charge‐transfer (LLCT) transition that produces TADF emission. The group of Thompson provided comprehensive understanding of TADF processes in two‐coordinate coinage metal complexes.^[^ ^3p–w^ ^]^ Subsequent research led by Che demonstrated effective suppression of a roll‐off in electroluminescence efficiencies.^[^ ^3l^ ^]^ The Che group also explored synthetic approaches, such as controlling steric encumbrance to increase *Φ* and incorporating multi‐resonance electronic character to improve color purity of emission.^[^ ^3^, ^5^ ^]^

Inspired by the trailblazing research outlined above, recent investigations have actively expanded the electroluminescence utility of two‐coordinate coinage metal complexes.^[^ ^3^, ^6^ ^]^ However, coinage metal complexes capable of producing long‐wavelength electroluminescence in the red to near‐infrared (NIR) regions remain scarce.^[^ ^3^, ^7^ ^]^ The scarcity primarily arises from the sharp decline in *Φ* within low‐energy emission regions, as evident from previous studies reporting high *Φ* values of blue‐ or green‐emissive coinage metal complexes,^[^ ^6^, ^8^ ^]^ but extremely low *Φ* values for red‐ or NIR‐emissive complexes.^[^ ^3^, ^7^ ^]^ Nevertheless, the importance of low‐energy‐emissive compounds persists, given their significant potential in diverse applications, including night vision devices, optical communication, and information security, thus driving a continued high demand for the development of narrow energy‐gap coinage metal complexes.

To meet the demand, we embarked on our research to develop two‐coordinate coinage metal complexes producing long‐wavelength emission. Among the coinage metal complexes, Au(I) complexes were chosen due to their high intrinsic stability and their strong oscillator strengths for electronic transitions.^[^ ^3^, ^4^ ^]^ Our molecular strategy was based on using a carbene ligand with the deep lowest‐unoccupied molecular orbital (LUMO) and an amido ligand with the shallow highest‐occupied molecular orbital (HOMO). Our main focus was placed on facilitating a radiative process, because most low‐energy emitters are inevitably subject to a high nonradiative decay rate (*k*_nr_) as governed by the energy gap law (see below).^[^ ^9^ ^]^ Given the relationship of Equation (1), one can improve *Φ* by maximizing the radiative decay rate (*k*_r_) even at a large *k*_nr_:

(1) $$\Phi =\frac{k_{r}}{k_{r}+k_{\mathrm{nr}}}$$

We aimed at identifying molecular factors that regulate *k*_r_ in two‐coordinate Au(I) complexes, thereby achieving high *Φ* values suitable for OLED applications with long‐wavelength emissions.

**Figure 1:**
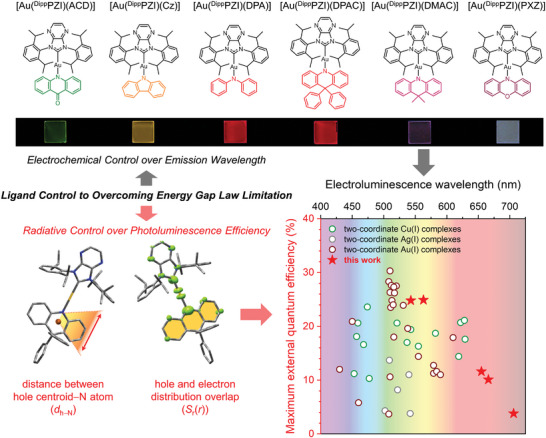
Ligand control strategy toward the development of two‐coordinate Au(I) complexes with low‐energy emissions. Top structures show the chemical structures of the Au(I) complexes with varied amido ligands. Bottom images illustrate the electronic effects exerted by the amido ligands, and comparisons of maximum external quantum efficiencies and peak electroluminescence wavelengths of devices with previous (empty circles) and our (red stars) coinage metal complexes. Refer to the main text for definitions of symbols.

In this research, we created a series of electroluminescent two‐coordinate Au(I) complexes featuring various amido ligands. We investigated the steady‐state and transient photoluminescence properties of these complexes, and analyzed them based on ground‐ (S_0_) and excited‐state geometries obtained from X‐ray single‐crystallography and quantum chemical calculations. Our analyses led to a molecular strategy to overcome the trade‐off limitation between the *Φ* and an emission wavelength. Subsequently, we fabricated multi‐layer OLEDs utilizing these Au(I) complexes as dopants, achieving long‐wavelength electroluminescence with a maximum external quantum efficiency (*EQE*_max_) of 7.0% and a peak wavelength (*λ*_EL_) of 680 nm that extended up to 706 nm at high doping ratios. The electroluminescence results expand the borderline of the *EQE*_max_−*λ*_EL_ space of coinage metal complexes (see the bottom panel in **Figure** **1**).

## 2 Results and Discussion

In the design of our Au(I) complexes, we used the 1,3‐bis(2,6‐diisopropylphenyl)pyrazinoimidazolium (^Dipp^PZI) carbene ligand with a deep LUMO energy. This carbene ligand was independently utilized by the Che group for the development of green‐emissive Cu(I) complexes.^[^ ^3^, ^5^ ^]^ Six amido ligands, including acridin‐9‐onide (ACD), cabazolide (Cz), *N*,*N*‐diphenylamide (DPA), 9,9‐diphenylacridinide (DPAC), 9,9‐dimethylacridinide (DMAC), and phenoxazinide (PXZ), were chosen to prepare a series of Au(I) complexes of [Au(^Dipp^PZI)(ACD)], [Au(^Dipp^PZI)(Cz)], [Au(^Dipp^PZI)(DPA)], [Au(^Dipp^PZI)(DPAC)], [Au(^Dipp^PZI)(DMAC)], and [Au(^Dipp^PZI)(PXZ)] (Figure 1). We anticipated two electronic effects imparted by the amido ligand control. The first electronic effect is fluorescence color tuning, a result of bandgap energy changes arising from the HOMO localized exclusively on the amido ligand. However, this color tuning inevitably lowers the *Φ* value because *k*_nr_ increases exponentially at a low emission energy (*E*_00_) following the energy gap law (Equation 2):^[^ ^9^ ^]^

(2) $$k_{\mathrm{nr}}=\frac{C^{2}\sqrt{2\pi }}{\hbar \sqrt{\hbar \omega_{M}E_{00}}}\exp\left[-\frac{E_{00}}{\hbar \omega_{M}}\left\{\ln\left(\frac{E_{00}}{l\lambda_{M}}-1\right)\right\}\right]$$

In Equation (2), *C* is the effective electronic coupling constant, *ħ* is the reduced Planck constant, *ω*_M_ is the frequency of the vibrational mode that promotes nonradiative processes of the emissive singlet excited (S_1_) state, *l* is the number of the vibrational mode, and *λ*_M_ is the reorganization energy.

It is envisioned that the limitation imposed by the energy gap law can be surpassed through the radiative control exerted by amido ligands, which represents the second electronic effect. According to Fermi's golden rule, *k*_r_ is predicted to increase in proportion to a cubic *E*_00_, as shown in Equation (3):^[^ ^10^ ^]^

(3) $$k_{r}=\frac{4E_{00}^{3}}{3\hbar c^{3}}{\left|\left\langle \phi_{S_{1}}|\mu |\phi_{S_{0}}\right\rangle \right|}^{2}$$

In Equation (3), *c* is the speed of light, *ϕ*_S1_ and *ϕ*_S0_ are wavefunctions of the S_1_ and S_0_ states, respectively, and *µ* is the electric transition dipole moment. Consequently, *k*_r_ must be low in low‐energy emission regions. We noted that *k*_r_ is proportional not only to *E*_00_^3^ but also to the transition probability ${|\left\langle \phi_{S_{1}}|\mu |\phi_{S_{0}}\right\rangle |}^{2}$. Specifically, *k*_r_ is enhanced through the overlap between the hole and electron distributions (*S*_r_(*r*)) in the S_1_ state that is directly proportional to the transition probability (see below and the Supporting Information for more discussion). The structurally varied amido ligands would exhibit different *S*_r_(*r*) values in their Au(I) complexes, enabling us to elucidate the molecular factor governing *k*_r_. Therefore, our structural control provides a valuable opportunity to establish the molecular design strategy toward improving *Φ* of long‐wavelength emissions from Au(I) complexes.

Six Au(I) complexes were prepared through the four‐step synthesis established by Hamze et al.^[^ ^3r^ ^]^ that involved a Pd‐catalyzed C─N coupling reaction, a condensation reaction to form a pyrazinoimidazolium precursor, the Au(I) complexation reaction, and the substitution of a chloro ligand with an amido ligand in the presence of NaO*^t^* Bu. The details of the synthetic procedures and spectroscopic identification data are shown in the Supporting Information. All complexes were highly soluble in polar organic solvents, such as CH_2_Cl_2_.

**Figure 2:**
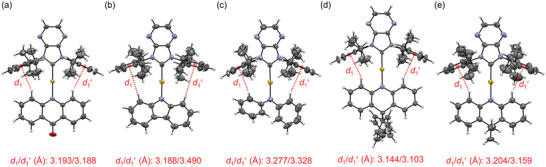
Oak Ridge Thermal Ellipsoid Plot (ORTEP) drawing at the 50% probability level of crystal structures: a) [Au(^Dipp^PZI)(ACD)], b) [Au(^Dipp^PZI)(Cz)], c) [Au(^Dipp^PZI)(DPA)], d) [Au(^Dipp^PZI)(DPAC)], and e) [Au(^Dipp^PZI)(DMAC)]. The *d*_1_ and *d*_1_′ are C─H···π distances between the H atoms in the amido ligand and the centroids of the peripheral Dipp units of the carbene ligand (red dotted lines).

Single crystals of the Au(I) complexes, excluding [Au(^Dipp^PZI)(PXZ)], could be obtained by layering pentane or hexane onto CH_2_Cl_2_ or by diffusing diethyl ether vapor. Crystallographic data and the key geometric parameters of the Au(I) complexes are compiled in the, Tables S1−S11 (Supporting Information). X‐ray crystallography reveals that the intermolecular Au···Au distance is >7.000 Å, which indicates the absence of aurophilic interactions (Figures S1−S5, Supporting Information). As depicted in **Figure** **2**, the *π*−planes of the carbene and amido ligands are co‐planar. The co‐planarity is due to the C─H·*π* interaction with distances ranging from 3.188 to 3.490 Å between the amido ligand and the Dipp moieties in the carbene ligand.

The amido ligand has a pronounced effect on the crystal structure geometry of the Au(I) complex. The bond length between Au and the amido N‐atom (*d*_Au−N_) increases in the order [Au(^Dipp^PZI)(Cz)] (2.021 Å) < [Au(^Dipp^PZI)(DMAC)] (2.027 Å) < [Au(^Dipp^PZI)(DPAC)] (2.036 Å) < [Au(^Dipp^PZI)(ACD)] (2.040 Å) < [Au(^Dipp^PZI)(DPA)] (2.063 Å). Apparently, the acyclic DPA ligand and the cyclic ACD ligand having a carbonyl unit elongate *d*_Au−N_. The dihedral angle, which is defined as the angle between two ligand planes, shows the similar trend in *d*_Au−N_: [Au(^Dipp^PZI)(Cz)] (0.3°) < [Au(^Dipp^PZI)(DPAC)] (2.8°) < [Au(^Dipp^PZI)(DMAC)] (3.5°) < [Au(^Dipp^PZI)(ACD)] (10.7°) < [Au(^Dipp^PZI)(DPA)] (24.1°). These structural parameters appear to align with the trend of the percent buried volume (%*V*_bur_) that quantifies the degree of encapsulation of the Au(I) center by ligands (Figure S6, Supporting Information). Overall, our X‐ray crystallographic studies indicate a loosening in the coordination geometry of Au(I) complexes upon the integration of an acyclic amido ligand and a cyclic amido ligand having a carbonyl unit.

**Figure 3:**
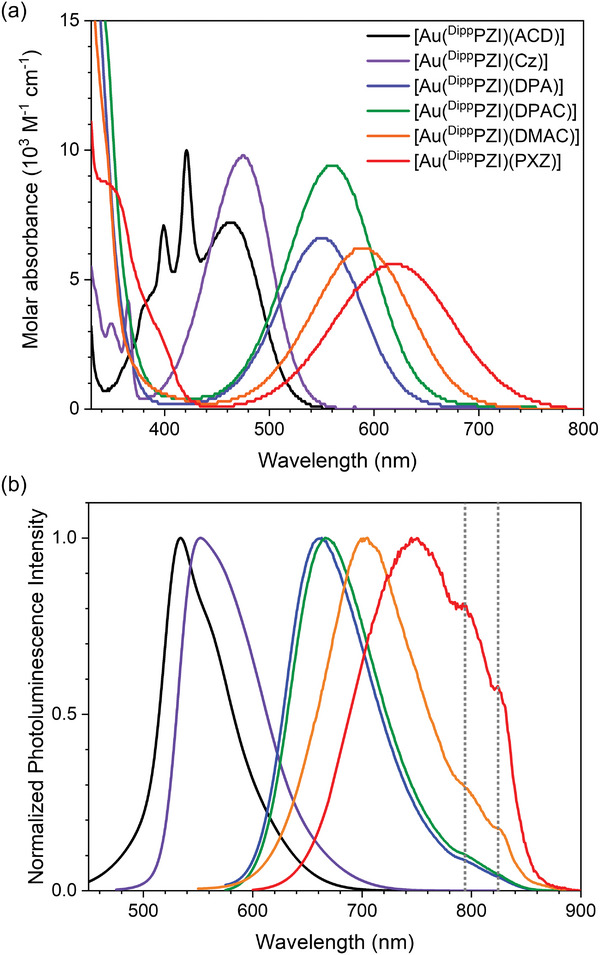
a) UV−Vis absorption spectra of 10 µm Au(I) complexes in toluene. b) Photoluminescence spectra of Zeonex thin films doped with 5 wt. % Au(I) complexes. Photoexcitation wavelengths: [Au(^Dipp^PZI)(ACD)], 472 nm; [Au(^Dipp^PZI)(Cz)], 473 nm; [Au(^Dipp^PZI)(DPA)], 575 nm; [Au(^Dipp^PZI)(DPAC)], 583 nm; [Au(^Dipp^PZI)(DMAC)], 596 nm; [Au(^Dipp^PZI)(PXZ)], 626 nm. The shoulder peaks at 795 and 824 nm (marked with vertical grey lines) are due to instrumental drifts.

**Figure** **3**a depicts the UV−Vis absorption spectra of 10 µm Au(I) complexes recorded in toluene. The LLCT transitions, characterized with the broad spectral shape, are clearly observed across the 400−800 nm range. The structured bands near 400 nm of [Au(^Dipp^PZI)(ACD)] are due to the local excitation centered at the ACD ligand.^[^ ^11^ ^]^ Peak wavelengths of the LLCT bands are distributed from 462 to 619 nm and their molar absorbance (*ε*) are in the range 5.6−9.8 × 10^3^ m ^−1^ cm^−1^ (see **Table** **1**). The molar absorbance values are similar to those of reported LLCT transitions for two‐coordinate coinage metal complexes (2−10 × 10^3^ m ^−1^ cm^−1^).^[^ ^3^, ^12^ ^]^

**Table 1:** Photophysical and electrochemical parameters for Au(I) complexes.

	*λ*_abs_ [nm (*ε*, 10^3^ m ^−1^ cm^−1^)]^a)^	*λ*_em_ [nm]^b)^	*τ*_obs_ [µs]^c)^	*Φ* ^d)^	*E*_ox_ (V vs SCE)^e)^	*E*_red_ (V vs SCE)^f)^	*k*_r_^TADF^ (10^5^ s^−1^)^g)^	*k*_nr_ (10^5^ s^−1^)^h)^	Δ*E*_S1−T1_ (meV)^i)^	*k*_ISC_ (10^8^ s^−1^)^j)^	*k*_rISC_ (10^8^ s^−1^)^k)^
[Au(^Dipp^PZI)(ACD)]	462 (7.2)	533	0.32	0.84	1.05	−1.49	26	5.0	40	21	6.6
[Au(^Dipp^PZI)(Cz)]	475 (9.8)	553	0.32	0.87	0.84	−1.62	27	2.4	55	22	4.5
[Au(^Dipp^PZI)(DPA)]	549 (6.6)	663	0.30	0.26	0.50	−1.58	8.7	25	49	7.9	1.5
[Au(^Dipp^PZI)(DPAC)]	560 (9.4)	666	0.26	0.22	0.54	−1.54	8.5	30	64	25	2.6
[Au(^Dipp^PZI)(DMAC)]	590 (6.2)	705	0.16	0.10	0.39	−1.54	6.3	56	52	3.8	4.7
[Au(^Dipp^PZI)(PXZ)]	619 (5.6)	750	0.019	N.D.^l)^	0.25	−1.49	N.D.^l)^	N.D.^l)^	N.D.^l)^	N.D.^l)^	N.D.^l)^

^a)^ Absorption peak wavelengths recorded for 10 µm Au(I) complex in deaerated toluene at 298 K;

^b)^ Photoluminescence peak wavelengths recorded for Zeonex thin films doped with 5 wt % Au(I) complexes (quartz substrates) at 298 K;

^c)^ Weighted average photoluminescence lifetime determined through triexponential decay fits of the photoluminescence decay traces monitored at emission peak wavelengths of Zeonex thin films doped with 5 wt % Au(I) complexes (quartz substrates) after pulsed laser excitation under 377 nm (time resolution: 32 ps);

^d)^ Photoluminescence quantum yields of Zeonex thin films doped with 5 wt. % Au(I) complexes (quartz substrates) determined absolutely by integrating over a sphere at 298 K;

^e)^ Oxidation;

^f)^ reduction potentials determined by cyclic and differential pulse voltammetry for anhydrous THF containing 2.0 mm samples and 0.10 m TBAPF_6_. A Pt working and a Pt counter electrodes, and an Ag/AgNO_3_ pseudo reference electrode were employed. Scan rate = 0.10 V s^−1^ (cyclic voltammetry) and 0.004 V s^−1^ (differential pulse voltammetry);

^g)^ Radiative decay rate of TADF emission, *k*_r_^TADF^ = *Φ* /*τ*_obs_;

^h)^ Nonradiative decay rate, *k*_nr_ = (1 − *Φ*) /*τ*_obs_;

^i)^ Energy difference between the S_1_ and T_1_ states determined through Boltzmann fitting of variable‐temperature *τ*_obs_ values (see Figures S10 and S11, Supporting Information);

^j)^ Rate constant for intersystem crossing computed from Equation S5 (Supporting Information);

^k)^ Rate constant for reverse intersystem crossing computed from Equation S4 (Supporting Information);

^l)^ Not determined due to weak emission.

The Au(I) complexes exhibit fluorescence emission over the broad range of peak emission wavelengths (*λ*_em_s) of 598−808 nm in toluene (Figure S7, Supporting Information). However, as with existing red‐emissive Au(I) complexes, there is a rapid decrease of *Φ* as low as < 0.01 in the long‐wavelength emission region (Table S12, Supporting Information).^[^ ^3^, ^7^ ^]^ We thus resorted to investigate emission behaviors for thin films of Zeonex doped with 5 wt. % Au(I) complexes. Photophysical parameters determined for thin films are compiled in Table 1. As shown in Figure 3b, thin films exhibit broad emissions with *λ*_em_ increasing from the 533 nm ([Au(^Dipp^PZI)(ACD)]) to 750 nm ([Au(^Dipp^PZI)(PXZ)]). The emission spectra of thin films are hypsochromically shifted relative to those of toluene, indicating rigidochromism. Corresponding Stokes shift ranges between 2765 and 3132 cm^−1^, which are similar to the previously reported values.^[^ ^3d^ ^]^ The Au(I) complexes show absolutely determined *Φ* values ranging from 0.10 to 0.84, which decreases with an increase of *λ*_em_. [Au(^Dipp^PZI)(PXZ)] did not produce a reliable *Φ* value, due to its low emission intensity. Evidently, there is an inverse relationship between *Φ* and *λ*_em_.

**Figure 4:**
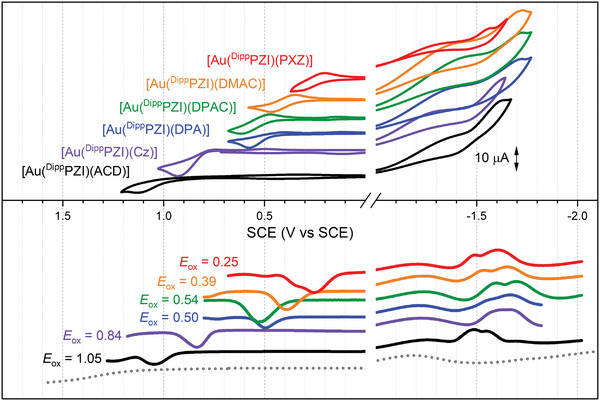
Cyclic (CV, upper panel) and differential pulse (DPV, lower panel) voltammograms of the Au(I) complexes. Conditions: Ar‐saturated, anhydrous THF containing a 2.0 mm Au(I) complex and a 0.10 m tetrabutylammonium hexafluorophosphate (TBAPF_6_) supporting electrolyte; a Pt disk and a Pt wire for the working and counter electrodes, respectively; an Ag/AgNO_3_ pseudo reference electrode; scan rate = 0.1 V s^−1^ (CV) and 0.004 V s^−1^ (DPV). The grey dotted line is the background signal of the blank solution containing only 0.10 m TBAPF_6_.

To understand the *λ*_em_ control by the amido ligands, electrochemical analyses using cyclic and differential pulse voltammetry were employed. Anhydrous THF containing a 2.0 mm Au(I) complex and a 0.10 m tetrabutylammonium hexafluorophosphate (TBAPF_6_) supporting electrolyte exhibits one‐electron oxidation and reduction processes of Au(I) complexes (**Figure** **4**). The oxidation potentials (*E*_ox_s) show a significant shift, depending on the amido ligand structure (1.05 to 0.25 V vs saturated calomel electrode (SCE)). On the other hand, the reduction potentials (*E*_red_s) remain relatively unperturbed, values ranging from −1.49 to −1.62 V vs SCE. This invariance suggests that the reduction process occurs mainly at the ^Dipp^PZI carbene ligand.^[^ ^3v^ ^]^ The corresponding electrochemical bandgap energy (*E*_g_^elec^), which is calculated through *E*_g_^elec^ = −*e*⋅(*E*_red_ − *E*_ox_) where *e* is the elementary charge, shows a linear correlation with the LLCT transition energy obtained from the UV−Vis absorption spectra (Figure S8, Supporting Information). Since our Au(I) complexes exhibit LLCT fluorescence, the emission energy of Au(I) complexes is governed primarily by the electron‐donating capability of amido ligands.

The Au(I) complexes exhibit short fluorescence lifetime (*τ*_obs_) in the range 0.019−0.32 µs at 298 K (Table 1; Figure S9, Supporting Information). Applying the two‐level Boltzmann model to the temperature‐dependent *τ*_obs_ values, we could deduce the energy difference between the singlet and triplet states (Δ*E*_S1−T1_) to be in the range 40−64 meV (Figures S10 and S11; Table S12, Supporting Information). The Δ*E*_S1−T1_ values comparable to the thermal energy at 298 K (ca. 25 meV) suggest the TADF nature. This notion is also supported by thermal enhancements (Figure S12, Supporting Information) and O_2_‐induced quenching (Figure S13, Supporting Information) of the photoluminescence intensity. Rate constants for intersystem crossing (*k*_ISC_) and reverse intersystem crossing (*k*_rISC_) could also be determined following the approach established by Ying et al.,^[^ ^3o^ ^]^ and are tabulated in Table 1. The *k*_nr_, which can be derived using the relationship *k*_nr_ = (1 − *Φ*)/*τ*_obs_, increases by an order of magnitude from the green‐emissive [Au(^Dipp^PZI)(ACD)] (5.0 × 10^5^ s^−1^) to the NIR‐emissive [Au(^Dipp^PZI)(DMAC)] (5.6 × 10^6^ s^−1^) (Table 1). At the same time, the rate constant for TADF (*k*_r_^TADF^), which is calculated through the relationship *k*_r_^TADF^ = *Φ*/*τ*_obs_, decreases from 2.6 × 10^6^ s^−1^ for [Au(^Dipp^PZI)(ACD)] to 6.3 × 10^5^ s^−1^ for [Au(^Dipp^PZI)(DMAC)]. The increase of *k*_nr_ and the decrease of *k*_r_^TADF^ indicate nonradiative and radiative control governed by *E*_00_ following Equations (2) and (3), respectively. In particular, the linearity found between the logarithm of *k*_nr_ and *E*_00_ presents compelling evidence for the adherence to the energy gap law (Figure S14, Supporting Information). Meanwhile, the absence of a substantial correlation between *k*_r_^TADF^ and the ratio of *k*_rISC_ to *k*_ISC_ indicates that the radiative process is not governed by a pre‐equilibrium between the singlet and triplet excited states through ISC/rISC cycles (Figure S15, Supporting Information).

To identify the molecular parameters governing the radiative control by the amido ligand, we conducted quantum chemical calculations for the Au(I) complexes at the CAM‐B3LYP level of theory. The optimized geometries of the S_0_ state exhibited a co‐planar structure, consistent with the single‐crystal structures (Table S13, Supporting Information). The relaxed geometries of the S_1_ and T_1_ states were obtained at time‐dependent (TD) CAM‐B3LYP level of theory, and the geometry parameters are summarized in the Tables S14−S19 and Figure S16 (Supporting Information). The T_1_ state geometry is characterized with a coplanar conformation between the two ligands, whereas the S_1_ state geometry has an orthogonal disposition between the two ligands. These geometry trends are in agreement with previous reports.^[^ ^3a,d,e,g,p,w^ ^]^

Electronic transitions were predicted for the T_1_ state geometry, as it with a longer lifetime better describes excited state behaviors.^[^ ^3^, ^13^ ^]^ The full calculation results for the TD‐DFT calculations are summarized in the Tables S20−S25 (Supporting Information). The lowest‐energy singlet transition is found to bear LLCT character with the HOMO and the LUMO being localized on the amido and carbene ligands, respectively.^[^ ^3^, ^4^ ^]^ The transition character is consistent with the notion derived from our UV−Vis absorption and fluorescence spectra. Notably, the HOMO and LUMO isosurfaces exhibit partial delocalization onto the 5*d*‐orbitals of Au, indicating a potential spin−orbit coupling exerted by Au.

Conventionally, natural transition orbitals (NTOs), which are obtained by transforming multiple molecular orbitals involved in the electronic transition into the most dominant contributing orbitals following Equation (4), are used to describe an electronic transition:^[^ ^3^, ^4^, ^14^ ^]^

(4) $$\left|{\left[U^{\prime }TV\right]}_{\mathrm{ij}}\right|=\sqrt{\lambda_{i}}\;\delta_{\mathrm{ij}}$$

In this equation, *U*′ is the occupied orbital matrix, *T* is the single particle transition density matrix, *V* is the virtual orbital matrix, *λ*_i_ is the associated eigenvalue, and *δ*_ij_ is a set of orbitals defined by unitary transformation. For instance, Li et al. compared NTOs of the electronic transitions in the S_0_ and T_1_ state geometries to elucidate nonradiative processes of two‐coordinate metal complexes.^[^ ^3u^ ^]^ The same group also discovered the correlation between *k*_r_^TADF^ and an NTO overlap integral.^[^ ^3^, ^4^ ^]^ Despite their utility, the limitation of NTOs is that they often represent only the most representative pair contributing to the electronic transition, as shown in Equation (4). This approach can lead to erroneous results because it may result in a partial loss of information about hole‐electron transitions where there are multiple NTOs describing electronic transition.^[^ ^14^, ^15^ ^]^

To circumvent the limitation, we chose to employ density‐based hole (*ρ* ^hole^(*r*)) and electron (*ρ* ^electron^(*r*)) distributions and their overlap (S_r_(*r*)) that is defined as follows:

(5) $$S_{r}\left(r\right)=\sqrt{\rho^{\mathrm{hole}}\left(r\right)\rho^{\mathrm{electron}}\left(r\right)}$$

**Figure 5:**
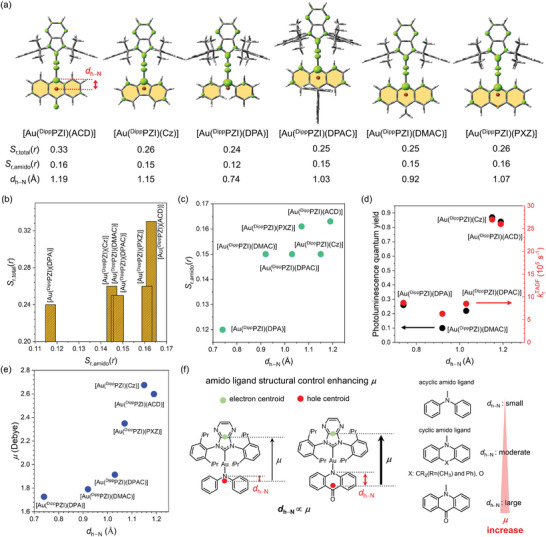
a) *S*_r,total_(*r*) isosurfaces of the singlet transition (green; isovalue = 0.001) for the Au(I) complexes. *S*_r,amido_(*r*) denotes *S*_r_(*r*) of the amido ligand fragment (yellow regions). *d*_h−N_ refers to the distance between the centroid of the hole distribution (dark‐orange sphere) and the N‐atom in the amido ligand. b) Correlation between *S*_r,total_(*r*) and *S*_r,amido_(*r*). c) Correlation between *S*_r,amido_(*r*) and *d*_h−N_. d) Correlations of *d*_h−N_ with *Φ* (black circles) and *k*_r_^TADF^ (red circles). e) Correlation between transition dipole moment (*µ*) and *d*_h−N_. f) Graphical description for the amido ligand structural effect on *µ*.

We reasoned that *S*_r_(*r*) provides a more precise representation for analyzing an electronic transition because it does not rely on multiple pairs of molecular orbitals but instead focuses on a single pair of hole and electron density distributions.^[^ ^16^ ^]^ Therefore, *S*_r_(*r*) is directly proportional to the term ${|\left\langle \phi_{S_{1}}|\mu |\phi_{S_{0}}\right\rangle |}^{2}$ in Equation (3), thereby quantifying radiative control more accurately. The *S*_r_(*r*) value over an entire structure of an Au(I) complex (denoted as *S*_r,total_(*r*)) could be calculated for the singlet transition in the T_1_ geometry, with the values ranging from 0.24 to 0.33 (**Figure** **5**a). As anticipated, the *k*_r_^TADF^ values typically exhibit an upward trend with increasing *S*_r,total_(*r*) until it reaches a value of 0.26. Beyond this threshold, *k*_r_^TADF^ levels off (Figure S17, Supporting Information). Interestingly, despite the decrease in emission energy, we consistently observed high *k*_r_^TADF^ values over 10^5^ s^−1^ in the red to NIR regions, prompting us to delve deeper into the underlying factors in more detail by analyzing *S*_r,total_(*r*) values. To facilitate this investigation, *S*_r,total_(*r*) was fragmented into three components within the Au(I) complex: the carbene ligand (referred to as *S*_r,carbene_(*r*)), Au(I) (referred to as *S*_r,Au_(*r*)), and the amido ligand (referred to as *S*_r,amido_(*r*)). The *S*_r,amido_(*r*) values varied within the range of 0.12−0.16, whereas *S*_r,carbene_(*r*) and *S*_r,Au_(*r*) remained relatively unchanged at 0.09−0.10 and 0.05−0.06, respectively (Table S26, Supporting Information). These findings are especially intriguing because they defy the intuitive expectation of the hole‐electron overlap being greatest at the Au(I), thereby adding an additional layer of interest to our investigation. Figure 5b demonstrates a rough proportionality relationship between *S*_r,total_(*r*) and *S*_r,amido_(*r*). This observation suggests that the radiative control exerted by the amido ligands is best represented by *S*_r,amido_(*r*).

We also discovered that *S*_r,amido_(*r*) is primarily governed by the distance between the centroid of the hole distribution and the N‐atom in the amido ligand (*d*_h−N_, refer to Figure 5a). Notably, *d*_h−N_ exhibits a substantial dependence on the structure of the amido ligand, with values from 0.74 to 1.19 Å. As depicted in Figure 5c, Au(I) complexes with larger *d*_h−N_ values yield higher *S*_r,amido_(*r*) values. Additionally, both *Φ* and *k*_r_^TADF^ values exhibit proportionality relationships with *d*_h−N_ (Figure 5d). The deviation of *Φ* from linearity is likely attributable to additional nonradiative processes. Moreover, *d*_h−N_ serves as a significant parameter intricately tied to radiative control due to its proportionality relationship with the transition dipole moment (*µ*) (Figure 5e). Note that this relationship is not limited to our Au(I) complexes, but is applicable to previous Au(I) complexes (Figure S18, Supporting Information). Therefore, *d*_h−N_ can serve as a pivotal parameter, intimately connected to both *S*_r,total_(*r*) and *µ*, within the context of radiative control. These findings suggest that maximizing *Φ* should involve increasing *d*_h−N_, providing valuable guidelines for molecular design, including the utilization of conjugated cyclic amido ligands and electron‐withdrawing substituents (Figure 5f). Ultimately, the incorporation of cyclic amido ligands in the investigated Au(I) complexes enables for the attainment of high *k*_r_^TADF^ values, even under low‐energy‐emissive regions, effectively surpassing the constraints imposed by the energy gap law.

To further confirm the radiative control, we computed theoretical *Φ* curves using *k*_nr_ and *k*_r_ values based on Equations (2) and (3), respectively (Figure S19a, Supporting Information). It is predicted that *k*_r_ increases with *E*_00_, whereas *k*_nr_ decreases sharply with *E*_00_. However, contrary to *k*_nr_, *k*_r_ depends on both *E*_00_ and the transition dipole moment. The *E*_00_ dependence of *k*_r_ is mitigated by the transition dipole moment. Correspondingly, a larger *Φ* value is obtainable from an emitter with a greater transition dipole moment than that with a smaller transition dipole moment. Experimental *Φ* values of our Au(I) complexes are located close to the theoretical curves of *Φ* having large transition dipole moment (Figure S19b, Supporting Information). This observation demonstrates that judicious control of *S*_r,total_(*r*) through *d*_h−N_ could overcome the constraints of *E*_00_ toward improved *Φ*.

**Figure 6:**
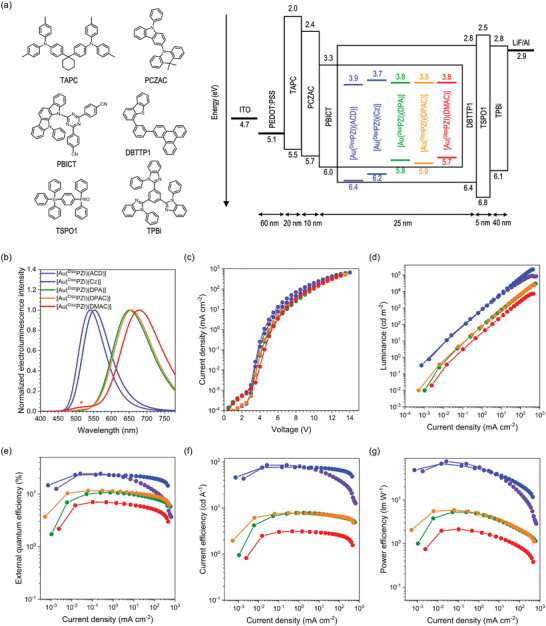
a) The configuration of the electroluminescence devices tested, and the chemical structures of constituent materials. b) Normalized electroluminescence spectra. The shoulder band marked with a red asterisk is due to the host emission. c) Current density−voltage curves. d) Luminance−current density curves. e) External quantum efficiency−current density curves. f) Current efficiency−current density curves. g) Power efficiency−current density curves.

In the concluding phase of our research, we fabricated multi‐layer OLEDs with the configuration of 50 nm indium tin oxide (ITO)/60 nm poly(3,4,‐ethylenedioxythiophene):polystyrenesulfonate (PEDOT:PSS)/20 nm 4,4′‐cyclohexylidene bis[*N,N*‐bis(4‐methylphenyl)aniline] (TAPC)/10 nm 9,9‐dimethyl‐10‐(9‐phenyl‐9*H*‐carbazol‐3‐yl)−9,10‐dihydroacridine (PCZAC)/25 nm 2‐phenyl‐4,6‐bis(12‐phenylindolo[2,3‐*a*]carbazole‐11‐yl)−1,3,5‐triazine (PBICT):4‐(3‐(triphenylene‐2‐yl)phenyl)dibenzo[*b*,*d*]thiophene (DBTTP1):Au(I) complex/5 nm diphenyl‐4‐triphenylsilylphenylphosphineoxide (TSPO1)/40 nm 2,2′,2′′‐(1,3,5‐benzenetriyl)‐tris(1‐phenyl‐1*H*‐benzimidazole) (TPBi)/1.5 nm LiF/200 nm Al. **Figure** **6**a depicts the chemical structures and energy levels of the constituent materials. PEDOT:PSS served as the hole‐injection layer, TAPC was the hole‐transporting material, PCZAC was the electron‐blocking material, PBICT was the TADF host, DBTTP1 was the triplet‐exciton‐guiding host,^[^ ^17^ ^]^ TSPO1 was the hole‐blocking layer, TPBi was the electron‐transporting layer, and LiF was the electron injection layer. The emission layer consisted of mixed hosts of PBICT and DBTTP1 (7:3, w/w) that were doped with Au(I) complexes in the range of 1−10 wt. %. [Au(^Dipp^PZI)(PXZ)] was excluded from our device fabrication due to its very low *Φ*. The Au(I) complexes were thermally stable, as evidenced by high temperature of 5 wt. % decomposition greater than 269 °C (Figures S20 and S21, Supporting Information). **Table** **2** summarizes electroluminescence performances of OLEDs with our Au(I) complex emitters.

**Table 2:** Summary of the electroluminescence characteristics of devices containing 3 wt. % Au(I) complexes.

	*V*_d_ [V]^a)^	*λ*_EL_ [nm]^b)^	(CIEx, CIEy)^c)^	*EQE*_max_ [%]^d)^	*CE*_max_ [cd A^−1^]^e)^	*PE*_max_ [lm W^−1^]^f)^
[Au(^Dipp^PZI)(ACD)]	4.5	539	(0.36, 0.59)	24.4	85.7	76.7
[Au(^Dipp^PZI)(Cz)]	4.8	551	(0.41, 0.56)	23.3	77.5	67.7
[Au(^Dipp^PZI)(DPA)]	7.3	650	(0.63, 0.36)	10.7	7.9	5.3
[Au(^Dipp^PZI)(DPAC)]	7.0	655	(0.63, 0.36)	11.6	7.8	5.9
[Au(^Dipp^PZI)(DMAC)]	8.2	680	(0.62, 0.36)	7.0	2.8	2.1

^a)^ Driving voltage at a luminance of 1000 cd m^−2^;

^b)^ Peak wavelength of the electroluminescence spectrum;

^c)^ Commission Internationale de l'Eclairage coordinates of devices recorded at luminances of 3000 cd m^−2^ ([Au(^Dipp^PZI)(ACD)] and [Au(^Dipp^PZI)(Cz)]), 5000 cd m^−2^ ([Au(^Dipp^PZI)(DPA)] and [Au(^Dipp^PZI)(DPAC)]), and 1000 cd m^−2^ ([Au(^Dipp^PZI)(DMAC)]);

^d)^ Maximum external quantum efficiency;

^e)^ Maximum current efficiency;

^f)^ Maximum power efficiency.

Figure 6b shows the electroluminescence spectra recorded for OLEDs containing 3 wt. % Au(I) complexes. Electroluminescence results for devices with 1, 5, and 10 wt. % Au(I) complex dopants are shown in the, Figures S22−S24 (Supporting Information). The *λ*_EL_ ranges 539−680 nm that is hypsochromically shifted with respect to each of the photoluminescence spectra, due likely to optical cavity effects. The [Au(^Dipp^PZI)(DMAC)] device exhibits the host emission at 520 nm, which disappears at higher doping concentrations at the expense of *EQE*_max_ (Figure S24, Supporting Information). The longest *λ*_EL_ was recorded for the [Au(^Dipp^PZI)(DMAC)] device (680 nm), which shifted bathochromically to 706 nm at increased doping concentrations (Figures S23 and S24, Supporting Information).

The current density (*J*) remains the highest for the device of [Au(^Dipp^PZI)(ACD)], and decreases following the order [Au(^Dipp^PZI)(Cz)] > [Au(^Dipp^PZI)(DPA)]  ∼ [Au(^Dipp^PZI)(DPAC)] ∼ [Au(^Dipp^PZI)(DMAC)] at the same voltage. This trend is rationalized by comparing the HOMO energy levels of the PBICT host and the Au(I) complex dopant, where the HOMO levels of [Au(^Dipp^PZI)(ACD)] and [Au(^Dipp^PZI)(Cz)] deeper than that of the PBICT host leads to higher *J* values. In contrast, the lower *J* values observed for the devices of [Au(^Dipp^PZI)(DPA)], [Au(^Dipp^PZI)(DPAC)], and [Au(^Dipp^PZI)(DMAC)] can be attributed to hole trapping within the Au(I) complex dopants. The lower luminance of the long‐wavelength‐emissive devices (i.e., devices of [Au(^Dipp^PZI)(DPA)], [Au(^Dipp^PZI)(DPAC)], and [Au(^Dipp^PZI)(DMAC)]) may, thus, be ascribed to the impaired *J* characteristics, as well as lower *Φ* values, while the maximum luminance as high as 7284 cd m^−2^ could be achieved for the [Au(^Dipp^PZI)(DMAC)] device at an *λ*_EL_ of 680 nm.

The green‐emissive [Au(^Dipp^PZI)(ACD)] device exhibits an *EQE*_max_ of 24.4% at an *λ*_EL_ of 539 nm. The *EQE*_max_ decreases as *λ*_EL_ increases, in the order of 23.3% for the [Au(^Dipp^PZI)(Cz)] device (*λ*_EL_ = 551 nm), 11.6% for the [Au(^Dipp^PZI)(DPAC)] device (*λ*_EL_ = 655 nm), 10.7% for the [Au(^Dipp^PZI)(DPA)] device (*λ*_EL_ = 650 nm), and 7.0% for the [Au(^Dipp^PZI)(DMAC)] device (*λ*_EL_ = 680 nm) (Figure 6e). A positive correlation is found between *Φ* and *EQE*_max_, which indicates that the hole trapping does not affect *EQE* values appreciably (Figure S25, Supporting Information). It should be emphasized that our Au(I) complexes set a new boundary in the *EQE*_max_−*λ*_EL_ space shown in Figure 1. In particular, the [Au(^Dipp^PZI)(DMAC)] device produces the longest *λ*_EL_ value (680 nm) among the two‐coordinate coinage metal complexes that have been reported thus far.^[^ ^3o^ ^]^ OLEDs based on our Au(I) complexes exhibit maximum current efficiencies from 2.8 to 85.7 cd A^−1^ (Figure 6f). As shown in Figure 6g, the power efficiency exhibits an analogous trend.

Finally, our Au(I) complex‐based OLEDs exhibit a suppressed roll‐off in *EQE*. The roll‐off quantified as (*EQE*_max_ − *EQE*_10_)/*EQE*_max_, where *EQE*_10_ denotes an *EQE* value at a *J* of 10 mA cm^−2^, is the largest for the [Au(^Dipp^PZI)(ACD)] device (0.25). Significantly reduced roll‐off behaviors are found for the [Au(^Dipp^PZI)(Cz)] device (0.048) and the [Au(^Dipp^PZI)(DPA)] device (0.024), whereas the devices of [Au(^Dipp^PZI)(DPAC)] (0.16) and [Au(^Dipp^PZI)(DMAC)] (0.12) show intermediate roll‐off behaviors. Particularly, devices of [Au(^Dipp^PZI)(Cz)] and [Au(^Dipp^PZI)(DPA)] exhibit roll‐off values of less than 5%, which is the lowest among the reported two‐coordinate Au(I) complexes.^[^ ^3^, ^7^, ^8^, ^12^ ^]^ Overall, the *λ*_EL_, the *EQE*, and the suppressed roll‐off behaviors highlight the electroluminescence utility of our Au(I) complexes.

## 3 Conclusion

Achieving long‐wavelength luminescence from two‐coordinate coinage metal complexes remains as a formidable challenge due to the significant decline in the luminescence efficiency. This limitation hampers the utilization of two‐coordinate metal complexes in full‐color OLEDs. In this research, we investigated the effects of the amido ligand structure on *λ*_em_ and *Φ* of two‐coordinate Au(I) complexes. It was found that the *λ*_em_ showed a correlation with *E*_ox_ influenced by the amido ligand, in accordance with the LLCT transition nature. *Φ* decreased rapidly as *λ*_em_ increased, indicating nonradiative control governed by the energy gap law. Remarkably, our quantum chemical calculations and photophysical analyses unveiled a radiative control mechanism capable of circumventing the limitation imposed by the energy gap law. Specifically, *k*_r_^TADF^ increases with *S*_r,amido_(*r*) that is directly proportional to *d*_h−N_. These findings offer insights into a molecular design strategy for maximizing *Φ* in two‐coordinate metal complexes. Finally, OLEDs based on one of our Au(I) complexes produced an *EQE*_max_ of 7.0% at an *λ*_EL_ of 680 nm that extended up to 706 nm at high concentrations. This represents the lowest‐energy electroluminescence achieved to date for two‐coordinate coinage metal complexes. It is envisioned that our research will guide the future molecular design strategies for developing high‐efficiency OLEDs that emit long‐wavelength emissions.

## Conflict of Interest

The authors declare no conflict of interest.

## Supporting information

Supporting Information

## Data Availability

The data that support the findings of this study are available from the corresponding author upon reasonable request.
