# Supplementary material for: Achieving Long‐Wavelength Electroluminescence Using Two‐Coordinate Gold(I) Complexes: Overcoming the Energy Gap Law
Source: Adv Sci (Weinh). 2023 Nov 12;11(1):2305745. doi: 10.1002/advs.202305745 (PMC10767458; doi:10.1002/advs.202305745)
Supplement: Supplementary file 1 — Supporting Information [file ADVS-11-2305745-s001.pdf]

ADVANCED SCIENCE
Open Access

Supporting Information

for *Adv. Sci.*, DOI 10.1002/advs.202305745

Achieving Long-Wavelength Electroluminescence Using Two-Coordinate Gold(I) Complexes: Overcoming the Energy Gap Law

Sreenivas Avula, Byung Hak Jhun, Unhyeok Jo, Seunga Heo, Jun Yeob Lee and Youngmin You**

Copyright WILEY-VCH Verlag GmbH & Co. KGaA, 69469 Weinheim, Germany, 2016.

Supporting Information

Achieving Long-Wavelength Electroluminescence Using Two-Coordinate Gold(I) Complexes: Overcoming the Energy Gap Law

Sreenivas Avula,[1†] Byung Hak Jhun,[1†] Unhyeok Jo,[2] Seunga Heo,[3] Jun Yeob Lee,[2] and Youngmin You[1]**

[1] Department of Chemical and Biomolecular Engineering, Yonsei University, Seoul 03722, Republic of Korea.
[2] School of Chemical Engineering, Sungkyunkwan University, Suwon, Gyeonggi-do 16419, Republic of Korea.
[3] Division of Chemical Engineering and Materials Science, Ewha Womans University, Seoul 03760, Republic of Korea.

Experimental Details

Materials. Commercially available chemicals, including 2,3-dichloropyrazine, 2,6-diisopropyl aniline, tris(dibenzylideneacetone)dipalladium(0) ($Pd_2(dba)_3$), tricyclohexylphosphine (PCy_3), trimethylsilyl chloride, sodium *tert*-butoxide (NaO^{t}Bu), chloro(dimethylsulfido)gold(I), potassium bis(trimethylsilyl)amide (KHMDS), and triethylorthoformate were used as received, unless otherwise stated. Toluene and tetrahydrofuran (THF) were distilled over sodium prior to use. Au(I) complexes were prepared following the methods developed by Hame *et al*. after minor modifications.[1] The synthesis of [Au(^{Dipp}PZI)(Cz)] was reported by Muniz *et al*.[2] All glassware, magnetic stir bars, syringes, and needles were dried in a convection oven at 120 °C. Reactions were monitored by using thin-layer chromatography (TLC). Commercial TLC plates (silica gel 254, Merck Co.) were developed, and the spots were visualized under UV irradiation at wavelengths of 254 or 365 nm. Column chromatography was performed on silica gel 60G (particle size 5–40 μm, Merck Co.). ^{1}H and ^{13}C{^{1}H} NMR spectra were collected using Bruker, Avance III-300 and 500 NMR spectrometers. Chemical shifts were referenced to tetramethylsilane. High-resolution mass spectra were acquired using JEOL, JMS-700GC or Agilent, 6890 series instruments. Elemental analyses were performed using a Thermo Fisher Scientific, Flash 2000 instrument.

Synthesis of N^2,N^3-bis(2,6-diisopropylphenyl)pyrazine-2,3-diamine. 2,3-Dichloropyrazine (5.00 g, 33.6 mmol), 2,6-diisopropylaniline (17.9 g, 101 mmol), and NaO^{t}Bu (9.70 g, 101 mmol) were added to an oven-dried 250 mL two-neck round-bottom flask equipped with a condenser and and magnetic stir bar. The mixtures were suspended in dry toluene (150 mL), and the mixture was purged with stream of an Ar gas for 15 min. After then, $Pd_2(dba)_3$ (7.68 g, 8.39 mmol) and PCy_3 (3.76 g, 13.4 mmol) dissolved in 150 mL dry tolyene were added to the reaction solution. The reaction mixture was allowed to be refluxed for 15 h under an Ar atmosphere. After cooling the reaction mixture, the solvent was evaporated under a reduced pressure. The crude product was diluted with CH_2Cl_2, and filtered through a celite pad to remove solid particles. The filtrate was poured onto water, and extracted with CH_2Cl_2 (100

mL × twice). The combined organic layers were dried over anhydrous $MgSO_4$, filtered, and concentrated. Finally, the product was purified by silica gel column chromatography using an eluent of EtOAc:*n*-hexane (3:17, v/v) to afford 5.80 g of red powders (40%). R_f = 0.4 (EtOAc:*n*-hexane = 1:4, v/v). ^{1}H NMR (300 MHz, CD_2Cl_2) δ (ppm): 7.45 (s, 2H), 7.29–7.32 (m, 2H), 7.22–7.25 (m, 4H), 5.72 (s, 2H), 3.11 (m, 4H), 1.17 (d, J = 6.6 Hz, 24 H). ^{13}C{^{1}H} NMR (126 MHz, CD_2Cl_2) δ (ppm): 146.8, 144.8, 134.7, 132.5, 128.0, 124.2, 29.2, 24.1, 23.9, 23.4. HR MS (FAB$^+$, *m*-NBA): calcd for $C_{28}H_{39}N_4$ ([M+H]$^+$), 431.3169; found, 431.3180.

Synthesis of 1,3-bis(2,6-diisopropylphenyl)-1H-imidazo[4,5-b]pyrazine-3-ium chloride. N^2,N^3-Bis(2,6-diisopropylphenyl)pyrazine-2,3-diamine (1.000 g, 2.32 mmol) was dissolved in 250 mL of triethylorthoformate in an oven-dried 250 mL round-bottom flask equipped with a condenser and a magnetic stir bar. The reaction mixture was heated at 150 °C for 48 h. Fractional distillation apparatus was equipped to the reaction flask, and ca. 200 mL of a liquid containing ethanol formed during the condensation reaction was distilled off over the course of 4 h. After cooling the reaction solution to room temperature, 50 mL of fresh triethylorthoformate and trimethylsilyl chloride (2.52 g, 23.2 mmol) were added and the reaction mixture was stirred at 50 °C for additional 8 h. After the reaction mixtures cooled to room temperature, the solvent was removed under a reduced pressure, and the resultant solids were washed with diethyl ether to give 0.550 g of brown solids (50%). ^{1}H NMR (300 MHz, CD_2Cl_2) δ (ppm): 14.14 (s, 1H), 8.88 (s, 2H), 7.73 (t, J = 7.8 Hz, 2H), 7.49 (d, J = 7.8 Hz, 4H), 2.18–2.26 (m, 4H), 1.31 (d, J = 6.6 Hz, 12H), 1.14 (d, J = 6.6 Hz, 12H). ^{13}C{^{1}H} NMR (126 MHz, CD_2Cl_2) δ (ppm): 163.5, 151.2, 151.0, 148.2, 147.0, 146.5, 146.2, 140.7, 138.2, 134.1, 133.1, 132.0, 130.4, 127.9, 126.8, 126.5, 125.4, 125.2, 124.9, 124.4, 124.2, 30.5, 30.4, 29.5, 28.9, 24.8, 24.2, 23.7. LR MS (FAB$^+$, *m*-NBA): 441 ([M–Cl]$^+$).

Synthesis of [Au(^{Dipp}PZI)(Cl)]. An oven-dried 50 mL Schlenk flask was charged with 1,3-bis(2,6-diisopropylphenyl)-1*H*-imidazo[4,5-*b*]pyrazine-3-ium chloride (0.500 g, 1.05 mmol) and freshly distilled THF. 1.0 M KHMDS (THF) (1.4 mL, 1.38 mmol) was dropwise added to the stirred reaction mixture, and the solution was stirred at room temperature for 1 h. Chloro(dimethylsulfido)gold(I) (0.412 g, 1.4 mmol) was added to the reaction flask. The reaction mixture was stirred at room temperature under an Ar atmosphere overnight. The reaction mixture was filtered through a layer of celite. The filtrate was collected and concentrated under a reduced pressure. Diethyl ether was poured onto the concentrated solution to afford precipitates. 0.250 g of brown solids were collected (35%). ^{1}H NMR (300 MHz, CD_2Cl_2) δ (ppm): 8.57 (s, 2H), 7.69 (t, J = 7.8 Hz, 2H), 7.46 (d, J = 7.8 Hz, 4H), 2.33 (m, 4H), 1.33 (d, J = 6.9 Hz, 12H), 1.10 (d, J = 6.9 Hz, 12H). ^{13}C{^{1}H} NMR (126 MHz, CD_2Cl_2) δ (ppm): 188.2, 147.0, 146.9, 142.7, 142.3, 140.4, 132.3, 132.1, 130.6, 125.3, 30.0, 24.8, 24.6, 24.1. LR MS (FAB$^+$, *m*-NBA): 637 ([M–Cl]$^+$) and 439 ([M–(AuCl)]$^+$).

Synthesis of [Au(^{Dipp}PZI)(ACD)]. To a flame-dried round-bottom flask, 9(10*H*)-acridone (0.072 g, 0.37 mmol) was added and dissolved in dry THF (10 mL). Na^{t}BuO (0.046 g, 0.48 mmol) was added to the stirred solution, and stirred for 0.5 h under an Ar atmosphere. [Au(^{Dipp}PZI)(Cl)] (0.250 g, 0.37 mmol) was subsequently added to the reaction mixture, which was stirred at room temperature for additional 12 h under dark. The reaction solution was diluted with 25 mL CH_2Cl_2, and filtered through celite. The filtrate was concentrated under a reduced pressure. The addition of 13 mL of CH_2Cl_2:diethyl ether (3:10, v/v) yielded yellow powders. Finally, purification by recrystallization in CH_2Cl_2 and *n*-pentane afforded 0.220 g of single crystals (72%). ^{1}H NMR (300 MHz, CD_2Cl_2) δ (ppm): 8.65 (s, 2H), 8.27

(dd, *J* = 8.4 and 1.8 Hz, 4 H), 7.86 (t, *J* = 7.8 Hz, 2H), 7.58 (d, *J* = 7.8 Hz, 4H), 7.12 (dt, *J* = 6.6 and 1.5 Hz, 2H), 6.94–7.00 (m, 4H), 2.47 (m, 4H), 1.30 (d, *J* = 6.9 Hz, 12H), 1.17 (d, *J* = 6.9 Hz, 12H). $^{13}C\{^1H\}$ NMR (126 MHz, CD_2Cl_2) δ(ppm): 189.8, 177.8, 150.6, 147.6, 142.3, 140.7, 133.7, 132.2, 131.4, 131.0, 127.2, 126.7, 125.4, 125.3, 123.3, 122.7, 121.5, 119.7, 117.7, 30.2, 24.5, 24.4. HR MS (FAB^+, *m*-NBA): calcd for $C_{42}H_{45}AuN_5O$ ($[M+H]^+$), 832.3284; found, 832.3282. Anal. Calcd for $C_{42}H_{44}AuN_5O$: C, 60.65; N, 8.42; H, 5.33. Found: C, 60.84; N, 8.30; H, 5.24%.

Characterization of [Au(^{Dipp}PZI)(Cz)]. ^1H NMR (300 MHz, CD_2Cl_2) δ(ppm): 8.60 (s, 2H), 7.88–7.91 (m, 2 H), 7.81 (t, *J* = 7.8 Hz, 2H), 7.57 (d, *J* = 7.8 Hz, 4H), 7.02–7.08 (m, 2H), 6.89 (td, *J* = 7.8 and 0.9 Hz, 2H), 6.63 (d, *J* = 8.1 Hz, 2H), 2.50 (m, 4H), 1.35 (d, *J* = 6.9 Hz, 12H), 1.16 (d, *J* = 6.9 Hz, 12H). $^{13}C\{^1H\}$ NMR (126 MHz, CD_2Cl_2) δ(ppm): 192.2, 149.7, 147.5, 141.9, 140.9, 132.1, 131.1, 125.3, 124.2, 124.1, 119.7, 116.7, 113.9, 30.1, 24.6, 24.3. HR MS (FAB^+, *m*-NBA): calcd for $C_{41}H_{44}AuN_5$ ($[M]^+$), 803.3262; found, 803.3262. Anal. Calcd for $C_{41}H_{44}AuN_5$: C, 61.27; N, 8.71; H, 5.52. Found: C, 61.51; N, 9.83; H, 5.54%.

Synthesis of [Au(^{Dipp}PZI)(DPA)]. [Au(^{Dipp}PZI)(DPA)] was prepared following the procedure used for the synthesis of [Au(^{Dipp}PZI)(ACD)], except employing diphenylamine (63 mg, 0.37 mmol) in place of 9(10*H*)-acridone. Purification by recrystallization with CH_2Cl_2 and diethyl ether gave 0.215 g of red crystalline solids (72%). ^1H NMR (300 MHz, CD_2Cl_2) δ(ppm): 8.53 (s, 2H), 7.73 (t, *J* = 7.8 Hz, 2H), 7.63 (d, *J* = 7.8 Hz, 4H), 6.80 (m, 4H), 6.61 (t, *J* = 2.1 and 1.2 Hz, 4H), 6.59 (m, 2H), 2.39 (m, 4H), 1.24 (d, *J* = 6.9 Hz, 12H), 1.11 (d, *J* = 6.9 Hz, 12H). $^{13}C\{^1H\}$ NMR (126 MHz, CD_2Cl_2) δ(ppm): 191.4, 154.4, 147.3, 141.5, 141.0, 131.8, 131.3, 128.7, 120.0, 118.1, 117.5, 30.3, 30.0, 24.3, 24.3, 24.1. HR MS (FAB^+, *m*-NBA): calcd for $C_{41}H_{46}AuN_5$ ($[M]^+$), 805.3419; found, 805.3423. Anal. Calcd for $C_{41}H_{46}AuN_5$: C, 61.11; N, 8.69; H, 6.75. Found: C, 61.99; N, 8.33; H, 6.06%.

Synthesis of [Au(^{Dipp}PZI)(DPAC)]. [Au(^{Dipp}PZI)(DPAC)] was prepared following the procedure used for the synthesis of [Au(^{Dipp}PZI)(ACD)], except employing 9,9-diphenyl-9,10-dihydroacridine (0.123 g, 0.37 mmol) in place of 9(10*H*)-acridone. Purification by recrystallization with CH_2Cl_2 and pentane gave 0.252 g of red crystalline solids (70%). ^1H NMR (300 MHz, CD_2Cl_2) δ(ppm): 8.54 (s, 2H), 7.74 (t, *J* = 7.8 Hz, 2H), 7.48 (d, *J* = 7.8 Hz, 4H), 7.06–7.12 (m, 6H), 6.85 (dt, *J* = 8.1 and 1.8 Hz, 4H), 6.61 (td, *J* = 8.1 and 1.8 Hz, 2H), 6.55 (dd, *J* = 8.1 and 1.8 Hz, 2H), 6.46–6.50 (m, 4H), 2.41 (m, 4H), 1.21 (d, *J* = 6.9 Hz, 12H), 1.11 (d, *J* = 6.9 Hz, 12H). $^{13}C\{^1H\}$ NMR (126 MHz, CD_2Cl_2) δ(ppm): 192.0, 149.3, 148.6, 147.4, 141.6, 141.0, 131.8, 131.1, 130.8, 130.1, 129.4, 127.6, 126.3, 125.8, 125.2, 117.6, 117.3, 57.2, 30.2, 30.0, 24.4, 24.3, 24.1. HR MS (FAB^+, *m*-NBA): calcd for $C_{54}H_{54}AuN_5$ ($[M]^+$), 969.4045; found, 969.4047. Anal. Calcd for $C_{54}H_{54}AuN_5$: C, 66.86; N, 7.22; H, 5.61. Found: C, 67.02; N, 7.62; H, 5.95%.

Synthesis of [Au(^{Dipp}PZI)(DMAC)]. [Au(^{Dipp}PZI)(DMAC)] was prepared following the procedure used for the synthesis of [Au(^{Dipp}PZI)(ACD)], except employing 9,9-dimethyl-9,10-dihydroacridine (0.077 g, 0.37 mmol) in place of 9(10*H*)-acridone. Purification by recrystallization with CH_2Cl_2 and hexane gave 0.150 g of red crystalline solids (48%). ^1H NMR (300 MHz, CD_2Cl_2) δ(ppm): 8.57 (s, 2H), 7.76 (t, *J* = 7.8 Hz, 2H), 7.51 (d, *J* = 7.8 Hz, 4H), 7.08 (dd, *J* = 7.2 and 1.8 Hz, 2H), 6.51–6.45 (m, 4H), 6.38 (dd, *J* = 7.8 and 1.8 Hz, 2H), 2.47 (m, 4H), 1.37 (s, 6H), 1.32 (d, *J* = 6.9 Hz, 12H), 1.14 (d, *J* = 6.9 Hz, 12H). $^{13}C\{^1H\}$ NMR (126 MHz, CD_2Cl_2) δ(ppm): 192.3, 148.4, 147.4, 147.0, 142.3, 141.6, 141.1, 132.1,

132.0, 131.9, 131.2, 130.4, 125.9, 125.7, 125.4, 125.3, 125.2, 121.0, 117.5, 117.2, 32.7, 30.1, 30.0, 24.6, 24.4, 24.4, 24.1. LR MS (FAB$^+$, *m*-NBA): 867 ([M+Na]$^+$), 637 ([M−DMAC]$^+$), and 439 ([M−(AuDMAC)]$^+$). Anal.Calcd for $C_{44}H_{50}AuN_5$: C, 62.48; N, 8.28; H, 5.96. Found: C, 62.56; N, 7.99; H, 5.81%.

Synthesis of [Au(^{Dipp}PZI)(PXZ)]. [Au(^{Dipp}PZI)(PXZ)] was prepared following the procedure used for the synthesis of [Au(^{Dipp}PZI)(ACD)], except employing 10*H*-phenoxazine (0.068 g, 0.37 mmol) in place of 9(10*H*)-acridone. Purification by repeated reprecipitation with CH_2Cl_2 and hexane gave 0.164 g of blue powders (54%). ^{1}H NMR (300 MHz, CD_2Cl_2) δ(ppm): 8.57 (d, J = 9.9 Hz, 2H), 7.66–7.74 (m, 2 H), 7.44–7.50 (m, 4H), 6.57–6.62 (m, 2H), 6.04–6.18 (m, 4H), 5.62–5.77 (m, 2H), 2.33–2.47 (m, 4H), 1.30–1.35 (m, 12H), 1.11–1.15 (m, 12H); ^{13}C{^{1}H} NMR (126 MHz, CD_2Cl_2) δ(ppm): 191.6, 191.1, 147.4, 147.2, 145.6, 144.5, 142.9, 142.3, 141.8, 141.6, 141.0, 140.4, 135.4, 132.1, 131.9, 131.8, 131.0, 130.6, 128.2, 125.3, 125.2, 124.7, 123.8, 123.1, 121.1, 118.8, 118.0, 116.7, 115.9, 115.7, 114.6, 114.4, 114.0, 30.1, 30.0, 24.6, 24.5, 24.4; LR MS (FAB$^+$, *m*-NBA): 819 ([M+H]$^+$), 637 ([M−PXZ]$^+$), and 429 ([M−(AuPXZ)]$^+$). Anal.Calcd for $C_{41}H_{44}AuN_5O$: C, 60.07; N, 8.54; H, 5.41. Found: C, 60.58; N, 8.40; H, 5.40%.

X-ray Crystallography. Single crystals suitable for X-ray crystallographic analysis were grown by layering pentane or hexane on top of dichloromethane containing Au(I) complexes, or by diffusion of diethyl ether vapor at room temperature. A single crystal was picked up from the solution and mounted on a Bruker SMART CCD diffractometer equipped with a graphite-monochromated Mo *K*α (λ = 0.71073 Å) radiation source under nitrogen cold stream at 223 K. The CCD data collected and integrated by using a Bruker-SAINT software program. Semi-empirical absorption corrections based on equivalent reflections were applied by Bruker SADABS. Structures were solved and refined using SHELXL97.[3] Hydrogen atoms were placed on the geometrically ideal positions. All non-hydrogen atoms were refined anisotropic thermal parameters. Crystal data for [Au(^{Dipp}PZI)(ACD)] (CCDC 2256305): $C_{42}H_{44}AuN_5O$, monoclinic, $P2_1/n$, Z = 4, a = 15.218(3), b = 14.128(3), c = 19.271(4) Å, α = 90°, β = 111.515(6)°, γ = 90°, V = 3854.5(15) Å^3, μ = 3.854 mm^{-1}, ρ_{calcd} = 1.433 Mg/m^3, R_1 = 0.0225, wR_2 = 0.0500 for 9595 unique reflections and 450 variables. Crystal data for [Au(^{Dipp}PZI)(Cz)] (CCDC 2256275): $C_{41}H_{44}AuN_5$, triclinic, $P\bar{1}$, Z = 8, a = 14.649(9), b = 17.043(10), c = 30.319(18) Å, α = 89.146(19)°, β = 82.470(19)°, γ = 88.197(19)°, V = 7500(8) Å^3, μ = 3.957 mm^{-1}, ρ_{calcd} = 1.424 Mg/m^3, R_1 = 0.0459, wR_2 = 0.0696 for 36720 unique reflections and 1724 variables. Crystal data for [Au(^{Dipp}PZI)(DPA)] (CCDC 2256306): $C_{41}H_{46}AuN_5$, orthorhombic, $Pbca$, Z = 8, a = 19.901(17), b = 16.114(16), c = 24.04(3) Å, α = 90°, β = 90°, γ = 90°, V = 7709(13) Å^3, μ = 3.850 mm^{-1}, ρ_{calcd} = 1.389 Mg/m^3, R_1 = 0.0554, wR_2 = 0.1202 for 9215 unique reflections and 432 variables. Crystal data for [Au(^{Dipp}PZI)(DPAC)] (CCDC 2256307): $C_{54}H_{54}AuN_5$, monoclinic, $P2_1/c$, Z = 4, a = 14.645(3), b = 14.248(3), c = 21.785(5) Å, α = 90°, β = 98.474(7)°, γ = 90°, V = 4496.2(16) Å^3, μ = 3.314 mm^{-1}, ρ_{calcd} = 1.433 Mg/m^3, R_1 = 0.0512, wR_2 = 0.0882 for 11156 unique reflections and 549 variables. Crystal data for [Au(^{Dipp}PZI)(DMAC)] (CCDC 2256308): $C_{44}H_{50}AuN_5$, orthorhombic, $P2_12_12_1$, Z = 8, a = 16.035(9), b = 20.631(10), c = 23.907(11) Å, α = 90°, β = 90°, γ = 90°, V = 7909(7) Å^3, μ = 3.756 mm^{-1}, ρ_{calcd} = 1.421 Mg/m^3, R_1 = 0.0449, wR_2 = 0.0752 for 20215 unique reflections and 920 variables.

Steady-State UV−Vis Absorption Measurements. UV−Vis absorption spectra were collected on a Shimazu, UV-1650 PC spectrometer at 298 K. Sample solutions were prepared prior to measurements at a concentration of 10 μM in toluene, unless otherwise stated. The solution was delivered into a quartz cell (Hellma, beam path length = 1.0 cm). Toluene containing 5 wt % of Zeonex and Au(I) complexes (5 wt % relative to Zeonex) were allowed to pass poly(tetrafluoroethylene) syringe filters (pore size = 0.45 μm), and spincoated onto 2 cm × 2 cm quartz plates using an EPLEX, SPIN-1200D spin coater.

Steady-state Photoluminescence Measurements. Photoluminescence spectra were collected on a PTI, Quanta Master 40 scanning spectrofluorimeter or a Varian, Cary Eclipse fluorescence spectrophotometer. The solutions and films used for the steady-state UV−Vis absorption studies were also used for the photoluminescence measurements. Solution samples were photoexcited at the following wavelengths: [Au(^{Dipp}PZI)(ACD)], 462 nm; [Au(^{Dipp}PZI)(Cz)], 475 nm; [Au(^{Dipp}PZI)(DPA)], 549 nm; [Au(^{Dipp}PZI)(DPAC)], 560 nm; [Au(^{Dipp}PZI)(DMAC)], 590 nm; [Au(^{Dipp}PZI)(PXZ)], 619 nm. Film samples were photoexcited at the following wavelengths: [Au(^{Dipp}PZI)(ACD)], 472 nm; [Au(^{Dipp}PZI)(Cz)], 473 nm; [Au(^{Dipp}PZI)(DPA)], 575 nm; [Au(^{Dipp}PZI)(DPAC)], 583 nm; [Au(^{Dipp}PZI)(DMAC)], 596 nm; [Au(^{Dipp}PZI)(PXZ)], 626 nm. The solutions were deaerated by bubbling Ar prior to the measurements. A quartz cell (Hellma, beam path length = 1.0 cm) was used for solution samples.

Determination of Relative Photoluminescence Quantum Yields. The photoluminescence quantum yield (Φ) was determined for 10 μM sample solutions dissolved in Ar-saturated toluene. The Φ was calculated using the equation $\Phi = \Phi_{ref} \times (I/I_{ref}) \times (A_{ref}/A) \times (n/n_{ref})^2$, where A, I, and n are the absorbance at the excitation wavelength, the integrated photoluminescence intensity, and the refractive index of the solvent, respectively. 9,10-Diphenylanthracene (Φ_{ref} = 1.00, toluene; λ_{ex} = 366 nm) was used as the reference material.[4] Photoluminescence spectra were collected at 298 K in the emission range 350−850 nm and were integrated using the OriginLab, OriginPro 2018 software.

Determination of Absolute Photoluminescence Quantum Yields. The Φ values of Zeonex films doped with 5 wt % samples (quartz substrates) were determined absolutely, using the methods embedded on a PTI, FelixGX software. The photon flux of an excitation beam ($I_{ex}(0)$) was quantified in the absence of a sample. The sample was placed into an integrating sphere (PTI), and an excitation beam was focused at the center of the sample. The flux of the excitation photons ($I_{ex}(s)$) was then quantitated. Finally, the photon flux of the photoluminescence emission from the sample (I_{em}) was measured under the following conditions: integration time, 0.1 s; step size, 0.0625 nm; emission range, 410−900 nm. The ratio between the absorbed photon flux and the emission photon flux corresponded to Φ: $\Phi = I_{em}/(I_{ex}(0) - I_{ex}(s))$. The measurement was repeated in triplicate for each fresh sample.

Photoluminescence Lifetime Measurements. Photoluminescence decay traces were collected by employing time-correlated single-photon-counting (TCSPC) technique using a PicoQuant, FluoTime 200 instrument through a motorized monochromator at the peak emission wavelength of each sample. A diode laser that produced 377 nm pulses (PicoQuant, LDH375) was driven by a PDL800-D driver (PicoQuant). The details about the measurement conditions are indicated in the caption of Figures S9 and S10. Temperature-dependent measurements were conducted in the range 79−315 K using an Oxford Instruments Omicron

Nanoscience, Optistat DN2 variable-temperature liquid N_2 cryostat equipped with a MercuryiTC temperature controller. Fluorescence decay profiles were fitted to a triexponential decay model embedded on an OriginLab, OriginPro 2022b software.

Determination of S_1–T_1 Energy Difference. The energy difference between the S_1 and the T_1 states (ΔE_{S1-T1}) was determined based on the variable-temperature photoluminescence lifetime data, following eq S1 developed by Hofbeck *et al.*[5]

$$\tau(T) = \frac{3 + exp\left(\frac{-\Delta E_{\mathrm{S1-T1}}}{k_B \mathrm{T}}\right)}{3k_{\mathrm{r}}^{T1} + k_{\mathrm{r}}^{S1}\exp\left(\frac{-\Delta E_{\mathrm{S1-T1}}}{k_B \mathrm{T}}\right)} \quad \text{(eq S1)}$$

In eq S1, τ(T) is the observed photoluminescence lifetime determined at temperature T, k_B is the Boltzmann constant, T is the absolute temperature, k_r^{S1} and k_r^{T1} are the radiative decay rates of the S_1 and T_1 states, respectively.

Determination of Rate Constants for ISC and rISC. The photophysical processes involved in TADF of the Au(I) complexes were analyzed based on eqs S2–S5 developed by Ying *et al.*:[6]

$$k_{\mathrm{r}}^{S1} = \Phi_{\mathrm{PF}} k_{\mathrm{PF}} + \Phi_{\mathrm{DF}} k_{\mathrm{DF}} \quad \text{(eq S2)}$$

$$k_{\mathrm{nr}}^{S1} = \frac{1 - \Phi_{\mathrm{total}}}{\Phi_{\mathrm{total}}} k_{\mathrm{r}}^{S1} \quad \text{(eq S3)}$$

$$k_{\mathrm{rISC}} \approx \frac{(\Phi_{\mathrm{PF}} + \Phi_{\mathrm{DF}}) k_{\mathrm{PF}} k_{\mathrm{DF}}}{\Phi_{\mathrm{PF}} k_{\mathrm{PF}} + \Phi_{\mathrm{DF}} k_{\mathrm{DF}}} = \frac{\Phi_{\mathrm{total}} k_{\mathrm{PF}} k_{\mathrm{DF}}}{k_{\mathrm{r}}^{S1}} \quad \text{(eq S4)}$$

$$k_{\mathrm{ISC}} = \frac{\Phi_{\mathrm{PF}} \Phi_{\mathrm{DF}} k_{\mathrm{PF}} k_{\mathrm{DF}} (k_{\mathrm{PF}} - k_{\mathrm{DF}})^2}{(\Phi_{\mathrm{PF}} k_{\mathrm{PF}} + \Phi_{\mathrm{DF}} k_{\mathrm{DF}})^2 k_{\mathrm{rISC}}} \approx \frac{\Phi_{\mathrm{PF}} \Phi_{\mathrm{DF}} (k_{\mathrm{PF}} - k_{\mathrm{DF}})^2}{k_{\mathrm{r}}^{S1} \Phi_{\mathrm{total}}} \quad \text{(eq S5)}$$

In these equations, k_{nr}^{S1} is the nonradiative decay rate of the S_1 state, k_{PF} is the rate for prompt fluorescence, k_{DF} is the rate for delayed fluorescence, k_{ISC} is the rate for intersystem crossing from the S_1 state to the T_1 state, k_{rISC} is the rate for reverse intersystem crossing, Φ_{PF} is the quantum yield for prompt fluorescence, Φ_{DF} is the quantum yield for delayed fluorescence, and $\Phi_{total} = \Phi_{PF} + \Phi_{DF}$.

Electrochemical Characterization. Cyclic and differential pulse voltammograms of the Ar-saturated anhydrous THF solutions (2.0 mL) containing 2.0 mM sample and 0.10 M tetrabutylammonium hexafluorophosphate were collected using a CH instruments, CHI630B instrument at 298 K. Standard three-electrode-assembly consisting of a platinum working electrode, a platinum counter electrode and an Ag/$AgNO_3$ pseudo reference electrode was used. The potentials were reported against saturated calomel electrode. The ferrocene/ferrocenium redox couple was employed as an external standard. Scan rates for cyclic and differential pulse voltammetries were 0.1 V s^{-1} and 4 mV s^{-1}, respectively.

Quantum Chemical Calculations. Quantum chemical calculations based on density functional theory (DFT) were performed using a Gaussian 09 program. Ground-state geometry optimization was performed using the B3LYP functional, the "double-ξ" quality LANL2DZ basis set for the Au atom, and the 6-311G(d) basis set for all other atoms (i.e., C, H, O, and N). A pseudo-potential (LANL2DZ) was used to replace the inner core electrons of the Au(I) atom, leaving the outer core and valence electrons. The S_1 and T_1 state geometries were optimized at the time-dependent (TD)-Coulomb-attenuated method (CAM)-B3LYP level of theory using basis sets identical to those used for the ground-state optimization. Single-point calculations were carried out for the optimized geometries using the TD-CAM-B3LYP functional with the same basis set used in geometry optimization. The conductor-like polarizable continuum model (CPCM) parameterized for THF was applied for TD-DFT calculations.

Determination of Overlap between Hole and Electron Distribution. The overlap between hole and electron distributions ($S_r(r)$) was calculated using a Multiwfn software,[7] following the method developed by Liu *et al*.[8] Hole and electron distributions of the excited state were calculated based on the protocols reported by Li *et al*.[9] and Jin *et al*.[10] The hole and electron distributions for each fragment of the carbene ligand, Au(I), and the amido ligand were computed using the Multiwfn program. Each fragment is defined as a selection of constituent atoms excluding hydrogen. $S_{r,total}(r)$ value was multiplied by the fragmental contribution to the entire structure to derive the $S_r(r)$ value for each fragment. This calculation automatically yielded the Cartesian coordinates for centroids of hole and electron distributions. The distance between the centroid of hole and the N atom in the amido ligand ($d_{h\text{–}N}$) was computed from their Cartesian coordinates.

Thermal Analysis. Thermogravimetric analysis (TGA) and differential scanning calorimetry (DSC) were simultaneously collected using a TA instruments, SDT Q600 in the range 30−500 °C under nitrogen atmosphere with heating rate of 10 °C min^{-1}. Au(I) complexes were loaded on an alumina pan. The decomposition temperature and melting temperature of the Au(I) complexes were characterized by the temperature at their 5 % of weight loss point in TGA and that at the endothermic peak in DSC.

Device Fabrication and Characterization. Multilayer OLEDs were constructed with the following configuration: 50 nm of indium tin oxide (ITO)/60 nm of poly(3,4-ethlyenedioxythiophene):polystyrenesulfonate (PEDOT:PSS)/20 nm of 4,4′-cyclohexylidenebis[*N*,*N*-bis(4-methylphenyl)aniline] (TAPC)/10 nm of 9,9-dimethyl-10-(9-phenyl-9H-carbazol-3-yl)-9,10-dihydroacridine (PCZAC)/emitting layer (25 nm)/5 nm of diphenyl-4-triphenylsilylphenyl-phosphineoxide (TSPO1)/40 nm of 2,2′,2″-(1,3,5-benzinetriyl)-tris(1-phenyl-1H-benzimidazole) (TPBi)/LiF (1.5 nm)/Al (200 nm).
The emitting layer involved a mixed host system of 2-phenyl-4,6-bis(12-phenylindolo[2,3-*a*]carbazole-11-yl)-1,3,5-triazine (PBICT) and 4-(3-(triphenylene-2-yl)phenyl)dibenzo[*b*,*d*]thiophene (DBTTP1) at a ratio of 70 and 30%, respectively. Au(I) complexes were co-evaporated with the hosts at doping concentrations of 1−10%. ITO served as the anode, PEDOT:PSS was a hole-injection layer, TAPC was a hole-transporting layer, PCZAC was an electron-blocking layer, PBICT was a TADF host, DBTTP1 was a triplet-exciton-guiding host,[11] TSPO1 was a hole-blocking layer, TPBi was an electron-transporting layer, LiF was an electron-injection layer, and Al served as a cathode. All devices were fabricated by thermal evaporation under 5 × 10^{-7} torr. Encapsulation was subsequently

conducted under a nitrogen atmosphere to prevent the degradation. The characteristics of devices were measured using a Keithley, 2400 source meter and a Konica Minolta, CS2000 spectroradiometer.

Table S1. Selected geometry parameters of the single crystal structures of Au(I) complexes

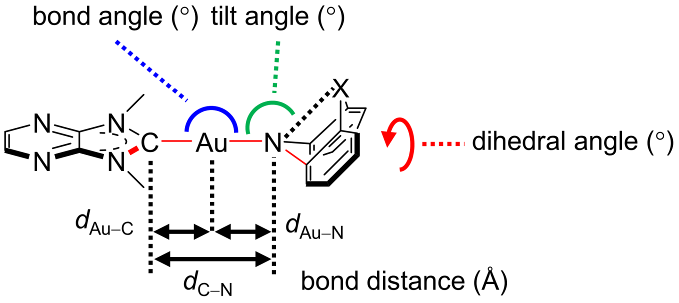

	bond length (Å)			bond angle (°)	dihedral angle (°)	tilt angle (°)
	d_{Au-C}	d_{Au-N}	d_{C-N}			
[Au(^{Dipp}PZI)(ACD)]	1.970(2)	2.040(2)	4.010	179.4(1)	10.7	176.9
[Au(^{Dipp}PZI)(Cz)]	1.982(5)	2.021(4)	3.998	174.4(2)	0.3	172.2
[Au(^{Dipp}PZI)(DPA)]	2.000(9)	2.063(7)	4.062	176.7(3)	24.1	not defined
[Au(^{Dipp}PZI)(DPAC)]	1.956(6)	2.036(5)	3.991	176.6(2)	2.8	178.4
[Au(^{Dipp}PZI)(DMAC)]	1.968(10)	2.027(8)	3.993	178.7(4)	3.5	172.9

Table S2. Crystallographic data for [Au(^{Dipp}PZI)(ACD)]

	[Au(^{Dipp}PZI)(ACD)]
Empirical formula	$C_{42}H_{44}AuN_5O$
Formula weight	831.79
Temperature (K)	223(2)
Wavelength (Å)	0.71073
Crystal system	monoclinic
Space group	$P2_1/n$
Unit cell dimensions	
a (Å)	15.218(3)
b (Å)	14.128(3)
c (Å)	19.271(4)
α (°)	90
β (°)	111.515(6)
γ (°)	90
Volume (Å^3)	3854.5(15)
Z	4
Calculated density (g cm^{-3})	1.433
Absorption coefficient (mm^{-1})	3.854
F(000)	1672
Crystal size (mm^3)	0.240 × 0.142 × 0.105
Theta range for data collection	2.135 to 28.412°.
Index ranges	$-15 \le h \le 20$, $-18 \le k \le 18$, $-25 \le l \le 25$
Reflections collected	84055
Independent reflections [*R*(int)]	9595 [0.0593]
Completeness to theta = 25.242°	99.90%
Absorption correction	Semi-empirical from equivalents
Max. and min. transmission	0.7457 and 0.6563
Refinement method	Full-matrix least-squares on F^2
Data / restraints / parameters	9595 / 0 / 450
Goodness-of-fit on F^2	1.03
Final *R* indices [I > 2sigma(*I*)]	R_1 = 0.0225, wR_2 = 0.0500
R indices (all data)	R_1 = 0.0302, wR_2 = 0.0534
Extinction coefficient	n/a
Largest diff. peak and hole (e Å^{-3})	0.461 and -0.416

Table S3. Selected bond distances and bond angles of [Au(^{Dipp}PZI)(ACD)]

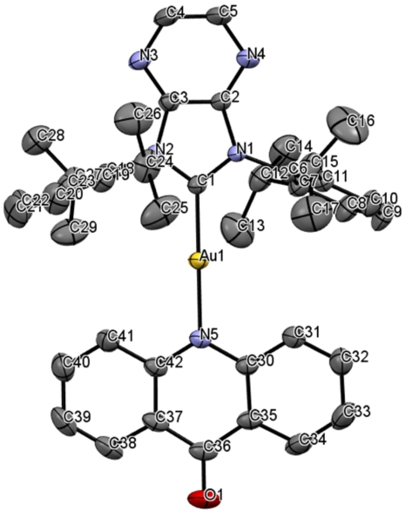

bond distances (Å)				bond angles (°)			
Au(1)-C(1)	1.970(2)	C(30)-C(31)	1.408(3)	C(1)-Au(1)-N(5)	179.42(9)	C(19)-C(18)-C(23)	124.1(2)
Au(1)-N(5)	2.0397(19)	C(30)-C(35)	1.413(3)	C(1)-N(1)-C(2)	109.60(18)	C(19)-C(18)-N(2)	117.6(2)
O(1)-C(36)	1.245(3)	C(31)-C(32)	1.373(4)	C(1)-N(1)-C(6)	122.15(18)	C(23)-C(18)-N(2)	118.3(2)
N(1)-C(1)	1.357(3)	C(32)-C(33)	1.406(4)	C(2)-N(1)-C(6)	128.16(18)	C(20)-C(19)-C(18)	116.7(3)
N(1)-C(2)	1.399(3)	C(33)-C(34)	1.359(4)	C(1)-N(2)-C(3)	109.83(19)	C(20)-C(19)-C(24)	121.0(3)
N(1)-C(6)	1.460(3)	C(34)-C(35)	1.411(4)	C(1)-N(2)-C(18)	123.40(18)	C(18)-C(19)-C(24)	122.3(2)
N(2)-C(1)	1.362(3)	C(35)-C(36)	1.460(4)	C(3)-N(2)-C(18)	126.37(18)	C(21)-C(20)-C(19)	121.0(3)
N(2)-C(3)	1.391(3)	C(36)-C(37)	1.454(4)	C(3)-N(3)-C(4)	111.5(2)	C(20)-C(21)-C(22)	120.7(3)
N(2)-C(18)	1.442(3)	C(37)-C(38)	1.408(4)	C(2)-N(4)-C(5)	111.7(2)	C(21)-C(22)-C(23)	121.0(3)
N(3)-C(3)	1.328(3)	C(37)-C(42)	1.421(3)	C(42)-N(5)-C(30)	118.40(19)	C(18)-C(23)-C(22)	116.5(2)
N(3)-C(4)	1.342(3)	C(38)-C(39)	1.360(4)	C(42)-N(5)-Au(1)	121.27(15)	C(18)-C(23)-C(27)	122.2(2)
N(4)-C(2)	1.328(3)	C(39)-C(40)	1.410(4)	C(30)-N(5)-Au(1)	120.31(15)	C(22)-C(23)-C(27)	121.2(2)
N(4)-C(5)	1.347(3)	C(40)-C(41)	1.370(4)	N(1)-C(1)-N(2)	107.15(18)	C(26)-C(24)-C(19)	111.1(3)
N(5)-C(42)	1.373(3)	C(41)-C(42)	1.419(3)	N(1)-C(1)-Au(1)	127.21(16)	C(26)-C(24)-C(25)	111.9(3)
N(5)-C(30)	1.387(3)			N(2)-C(1)-Au(1)	125.61(16)	C(19)-C(24)-C(25)	110.9(3)
C(2)-C(3)	1.386(3)			N(4)-C(2)-C(3)	124.1(2)	C(23)-C(27)-C(28)	112.3(2)
C(4)-C(5)	1.394(4)			N(4)-C(2)-N(1)	129.2(2)	C(23)-C(27)-C(29)	110.9(2)
C(6)-C(11)	1.394(3)			C(3)-C(2)-N(1)	106.71(19)	C(28)-C(27)-C(29)	110.9(2)
C(6)-C(7)	1.398(3)			N(3)-C(3)-C(2)	124.9(2)	N(5)-C(30)-C(31)	119.5(2)
C(7)-C(8)	1.392(3)			N(3)-C(3)-N(2)	128.4(2)	N(5)-C(30)-C(35)	122.6(2)
C(7)-C(12)	1.520(4)			C(2)-C(3)-N(2)	106.69(19)	C(31)-C(30)-C(35)	117.9(2)
C(8)-C(9)	1.382(4)			N(3)-C(4)-C(5)	123.8(2)	C(32)-C(31)-C(30)	121.3(2)
C(9)-C(10)	1.377(4)			N(4)-C(5)-C(4)	123.8(2)	C(31)-C(32)-C(33)	120.3(3)
C(10)-C(11)	1.404(3)			C(11)-C(6)-C(7)	124.2(2)	C(34)-C(33)-C(32)	119.6(3)
C(11)-C(15)	1.514(4)			C(11)-C(6)-N(1)	117.9(2)	C(33)-C(34)-C(35)	121.3(3)
C(12)-C(14)	1.528(4)			C(7)-C(6)-N(1)	117.8(2)	C(34)-C(35)-C(30)	119.6(2)
C(12)-C(13)	1.538(4)			C(8)-C(7)-C(6)	116.4(2)	C(34)-C(35)-C(36)	119.9(2)
C(15)-C(16)	1.512(5)			C(8)-C(7)-C(12)	120.1(2)	C(30)-C(35)-C(36)	120.5(2)
C(15)-C(17)	1.515(5)			C(6)-C(7)-C(12)	123.4(2)	O(1)-C(36)-C(37)	122.4(3)
C(18)-C(19)	1.393(3)			C(9)-C(8)-C(7)	121.2(3)	O(1)-C(36)-C(35)	122.7(3)
C(18)-C(23)	1.393(3)			C(10)-C(9)-C(8)	120.9(2)	C(37)-C(36)-C(35)	114.9(2)
C(19)-C(20)	1.389(4)			C(9)-C(10)-C(11)	120.6(3)	C(38)-C(37)-C(42)	119.2(3)
C(19)-C(24)	1.523(4)			C(6)-C(11)-C(10)	116.6(2)	C(38)-C(37)-C(36)	119.8(2)
C(20)-C(21)	1.376(5)			C(6)-C(11)-C(15)	123.5(2)	C(42)-C(37)-C(36)	120.9(2)

C(21)-C(22)	1.379(4)	C(10)-C(11)-C(15)	119.9(2)	C(39)-C(38)-C(37)	121.4(3)
C(22)-C(23)	1.393(4)	C(7)-C(12)-C(14)	112.3(2)	C(38)-C(39)-C(40)	119.8(3)
C(23)-C(27)	1.519(3)	C(7)-C(12)-C(13)	110.4(2)	C(41)-C(40)-C(39)	120.4(3)
C(24)-C(26)	1.521(5)	C(14)-C(12)-C(13)	110.0(3)	C(40)-C(41)-C(42)	120.8(3)
C(24)-C(25)	1.525(4)	C(16)-C(15)-C(11)	110.8(3)	N(5)-C(42)-C(41)	119.5(2)
C(27)-C(28)	1.528(4)	C(16)-C(15)-C(17)	111.0(3)	N(5)-C(42)-C(37)	122.2(2)
C(27)-C(29)	1.528(4)	C(11)-C(15)-C(17)	111.7(2)	C(41)-C(42)-C(37)	118.3(2)

Table S4. Crystallographic data for [Au(^{Dipp}PZI)(Cz)]

	[Au(^{Dipp}PZI)(Cz)]
Empirical formula	$C_{41}H_{44}AuN_5$
Formula weight	803.78
Temperature (K)	223(2)
Wavelength (Å)	0.71073
Crystal system	triclinic
Space group	*P*-1
Unit cell dimensions	
a (Å)	14.649(9)
b (Å)	17.043(10)
c (Å)	30.319(18)
α (°)	89.146(19)
β (°)	82.470(19)
γ (°)	88.197(19)
Volume (Å^3)	7500(8)
Z	8
Calculated density (g cm^{-3})	1.424
Absorption coefficient (mm^{-1})	3.957
F(000)	3232
Crystal size (mm^3)	0.190 × 0.165 × 0.150
Theta range for data collection	1.797 to 28.232°
Index ranges	$-19 \le h \le 19$, $-22 \le k \le 22$, $-40 \le l \le 40$
Reflections collected	348632
Independent reflections [*R*(int)]	36720 [0.1464]
Completeness to theta = 25.242°	100.00%
Absorption correction	Semi-empirical from equivalents
Max. and min. transmission	0.7457 and 0.6110
Refinement method	Full-matrix least-squares on F^2
Data / restraints / parameters	36720 / 0 / 1724
Goodness-of-fit on F^2	1.009
Final *R* indices [I > 2sigma(*I*)]	R_1 = 0.0459, wR_2 = 0.0696
R indices (all data)	R_1 = 0.1098, wR_2 = 0.0846
Extinction coefficient	n/a
Largest diff. peak and hole (e Å^{-3})	0.950 and -1.818

Table S5. Selected bond distances and bond angles of [Au(^{Dipp}PZI)(Cz)]

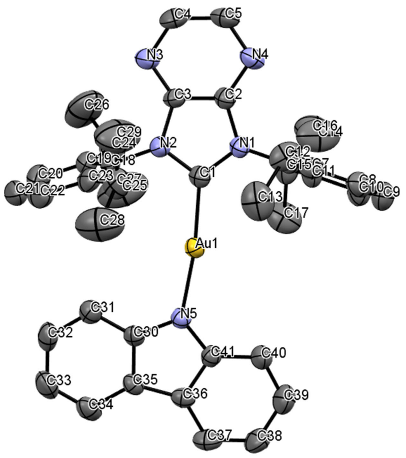

bond distances (Å)				bond angles (°)			
Au(1)-C(1)	1.985(5)	C(24)-C(25)	1.511(8)	C(1)-Au(1)-N(5)	172.26(19)	C(11)-C(15)-C(17)	111.1(5)
Au(1)-N(5)	2.022(4)	C(27)-C(29)	1.502(9)	C(1)-N(1)-C(2)	109.9(4)	C(11)-C(15)-C(16)	110.0(5)
N(1)-C(1)	1.372(6)	C(27)-C(28)	1.518(8)	C(1)-N(1)-C(6)	124.2(4)	C(17)-C(15)-C(16)	111.7(5)
N(1)-C(2)	1.403(6)	C(30)-C(31)	1.400(7)	C(2)-N(1)-C(6)	125.8(4)	C(23)-C(18)-C(19)	123.9(5)
N(1)-C(6)	1.448(6)	C(30)-C(35)	1.419(6)	C(1)-N(2)-C(3)	110.2(4)	C(23)-C(18)-N(2)	118.4(5)
N(2)-C(1)	1.370(6)	C(31)-C(32)	1.381(7)	C(1)-N(2)-C(18)	123.5(4)	C(19)-C(18)-N(2)	117.7(5)
N(2)-C(3)	1.397(6)	C(32)-C(33)	1.397(8)	C(3)-N(2)-C(18)	126.2(4)	C(18)-C(19)-C(20)	116.5(6)
N(2)-C(18)	1.459(6)	C(33)-C(34)	1.374(8)	C(3)-N(3)-C(4)	111.7(5)	C(18)-C(19)-C(24)	122.1(5)
N(3)-C(3)	1.322(6)	C(34)-C(35)	1.407(7)	C(2)-N(4)-C(5)	111.7(5)	C(20)-C(19)-C(24)	121.3(6)
N(3)-C(4)	1.340(7)	C(35)-C(36)	1.435(7)	C(30)-N(5)-C(41)	105.9(4)	C(21)-C(20)-C(19)	120.8(7)
N(4)-C(2)	1.329(6)	C(36)-C(37)	1.413(7)	C(30)-N(5)-Au(1)	121.8(3)	C(22)-C(21)-C(20)	121.0(7)
N(4)-C(5)	1.345(7)	C(36)-C(41)	1.434(6)	C(41)-N(5)-Au(1)	131.7(3)	C(21)-C(22)-C(23)	120.7(7)
N(5)-C(30)	1.391(6)	C(37)-C(38)	1.371(8)	N(2)-C(1)-N(1)	106.4(4)	C(18)-C(23)-C(22)	117.0(6)
N(5)-C(41)	1.393(6)	C(38)-C(39)	1.405(8)	N(2)-C(1)-Au(1)	122.8(4)	C(18)-C(23)-C(27)	122.1(5)
C(2)-C(3)	1.389(7)	C(39)-C(40)	1.383(7)	N(1)-C(1)-Au(1)	130.6(4)	C(22)-C(23)-C(27)	120.9(6)
C(4)-C(5)	1.405(8)	C(40)-C(41)	1.392(7)	N(4)-C(2)-C(3)	124.3(5)	C(26)-C(24)-C(25)	110.3(6)
C(6)-C(11)	1.397(7)			N(4)-C(2)-N(1)	129.0(5)	C(26)-C(24)-C(19)	112.8(6)
C(6)-C(7)	1.405(7)			C(3)-C(2)-N(1)	106.7(4)	C(25)-C(24)-C(19)	111.4(6)
C(7)-C(8)	1.385(7)			N(3)-C(3)-C(2)	124.9(5)	C(29)-C(27)-C(23)	112.5(6)
C(7)-C(12)	1.516(7)			N(3)-C(3)-N(2)	128.3(5)	C(29)-C(27)-C(28)	110.6(6)

C(8)-C(9)	1.367(8)			C(2)-C(3)-N(2)	106.8(4)	C(23)-C(27)-C(28)	113.4(6)
C(9)-C(10)	1.378(8)			N(3)-C(4)-C(5)	123.8(5)	N(5)-C(30)-C(31)	128.0(5)
C(10)-C(11)	1.386(7)			N(4)-C(5)-C(4)	123.6(5)	N(5)-C(30)-C(35)	111.5(4)
C(10)-H(10)	0.94			C(11)-C(6)-C(7)	123.4(5)	C(31)-C(30)-C(35)	120.5(5)
C(11)-C(15)	1.519(7)			C(11)-C(6)-N(1)	118.6(5)	C(32)-C(31)-C(30)	118.2(5)
C(12)-C(14)	1.527(8)			C(7)-C(6)-N(1)	117.9(4)	C(31)-C(32)-C(33)	121.6(5)
C(12)-C(13)	1.551(8)			C(8)-C(7)-C(6)	116.3(5)	C(34)-C(35)-C(36)	134.3(5)
C(15)-C(17)	1.533(7)			C(8)-C(7)-C(12)	122.3(5)	C(30)-C(35)-C(36)	105.8(4)
C(15)-C(16)	1.537(7)			C(6)-C(7)-C(12)	121.4(5)	C(37)-C(36)-C(41)	118.5(5)
C(18)-C(23)	1.387(7)			C(9)-C(8)-C(7)	122.0(6)	C(37)-C(36)-C(35)	135.3(5)
C(18)-C(19)	1.390(7)			C(8)-C(9)-C(10)	120.1(6)	C(41)-C(36)-C(35)	106.2(4)
C(19)-C(20)	1.398(8)			C(9)-C(10)-C(11)	121.6(6)	C(38)-C(37)-C(36)	120.2(5)
C(19)-C(24)	1.519(8)			C(10)-C(11)-C(6)	116.6(5)	C(37)-C(38)-C(39)	120.1(5)
C(20)-C(21)	1.380(9)			C(10)-C(11)-C(15)	120.9(5)	C(40)-C(39)-C(38)	121.8(6)
C(21)-C(22)	1.365(9)			C(6)-C(11)-C(15)	122.5(5)	C(39)-C(40)-C(41)	118.5(5)
C(22)-C(23)	1.399(8)			C(7)-C(12)-C(14)	113.8(6)	C(40)-C(41)-N(5)	128.7(5)
C(23)-C(27)	1.516(8)			C(7)-C(12)-C(13)	110.9(5)	C(40)-C(41)-C(36)	120.8(5)
C(24)-C(26)	1.501(8)			C(14)-C(12)-C(13)	109.9(5)	N(5)-C(41)-C(36)	110.5(4)

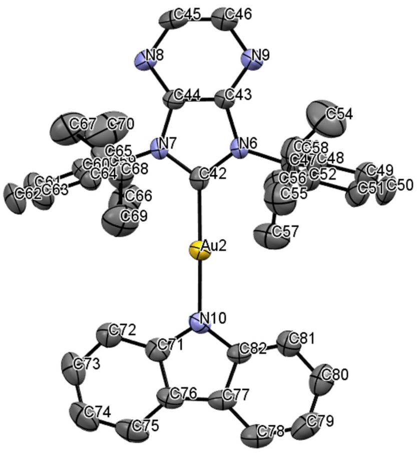

bond distances (Å)				bond angles (°)			
Au(2)-C(42)	1.982(5)	C(71)-C(72)	1.391(7)	C(42)-Au(2)-N(10)	174.41(18)	C(57)-C(56)-C(58)	109.5(5)

Au(2)-N(10)	2.021(4)	C(71)-C(76)	1.424(7)	C(42)-N(6)-C(43)	109.8(4)	C(60)-C(59)-C(64)	124.1(5)
N(6)-C(42)	1.373(6)	C(72)-C(73)	1.384(7)	C(42)-N(6)-C(47)	125.0(4)	C(60)-C(59)-N(7)	118.3(5)
N(6)-C(43)	1.399(6)	C(73)-C(74)	1.400(8)	C(43)-N(6)-C(47)	125.1(4)	C(64)-C(59)-N(7)	117.6(5)
N(6)-C(47)	1.446(6)	C(74)-C(75)	1.368(8)	C(42)-N(7)-C(44)	109.7(4)	C(59)-C(60)-C(61)	116.2(5)
N(7)-C(42)	1.368(6)	C(75)-C(76)	1.414(7)	C(42)-N(7)-C(59)	124.2(4)	C(59)-C(60)-C(65)	121.3(5)
N(7)-C(44)	1.399(6)	C(76)-C(77)	1.432(7)	C(44)-N(7)-C(59)	125.9(4)	C(61)-C(60)-C(65)	122.4(5)
N(7)-C(59)	1.455(6)	C(77)-C(78)	1.407(7)	C(44)-N(8)-C(45)	110.5(5)	C(62)-C(61)-C(60)	121.6(6)
N(8)-C(44)	1.332(6)	C(77)-C(82)	1.422(7)	C(43)-N(9)-C(46)	111.1(5)	C(63)-C(62)-C(61)	120.7(6)
N(8)-C(45)	1.357(7)	C(78)-C(79)	1.389(8)	C(82)-N(10)-C(71)	106.6(4)	C(62)-C(63)-C(64)	121.6(6)
N(9)-C(43)	1.326(6)	C(79)-C(80)	1.405(8)	C(82)-N(10)-Au(2)	128.4(3)	C(63)-C(64)-C(59)	115.8(5)
N(9)-C(46)	1.348(7)	C(80)-C(81)	1.377(7)	C(71)-N(10)-Au(2)	124.2(3)	C(63)-C(64)-C(68)	122.4(5)
N(10)-C(82)	1.388(6)	C(81)-C(82)	1.390(7)	N(7)-C(42)-N(6)	106.5(4)	C(59)-C(64)-C(68)	121.7(5)
N(10)-C(71)	1.393(6)			N(7)-C(42)-Au(2)	126.7(4)	C(66)-C(65)-C(60)	110.2(5)
C(43)-C(44)	1.381(7)			N(6)-C(42)-Au(2)	126.2(4)	C(66)-C(65)-C(67)	110.4(5)
C(45)-C(46)	1.377(8)			N(9)-C(43)-C(44)	125.0(5)	C(60)-C(65)-C(67)	112.7(5)
C(47)-C(48)	1.386(7)			N(9)-C(43)-N(6)	128.2(5)	C(70)-C(68)-C(69)	114.0(6)
C(47)-C(52)	1.398(7)			C(44)-C(43)-N(6)	106.7(4)	C(70)-C(68)-C(64)	111.5(5)
C(48)-C(49)	1.408(8)			N(8)-C(44)-C(43)	124.8(4)	C(69)-C(68)-C(64)	111.6(5)
C(48)-C(53)	1.533(8)			N(8)-C(44)-N(7)	128.0(5)	C(72)-C(71)-N(10)	128.4(5)
C(49)-C(50)	1.376(8)			C(43)-C(44)-N(7)	107.2(4)	C(72)-C(71)-C(76)	120.9(5)
C(50)-C(51)	1.368(8)			N(8)-C(45)-C(46)	124.6(5)	N(10)-C(71)-C(76)	110.7(5)
C(51)-C(52)	1.391(7)			N(9)-C(46)-C(45)	124.0(5)	C(73)-C(72)-C(71)	117.9(5)
C(52)-C(56)	1.520(8)			C(48)-C(47)-C(52)	123.6(5)	C(72)-C(73)-C(74)	122.7(6)
C(53)-C(54)	1.521(8)			C(48)-C(47)-N(6)	117.9(5)	C(75)-C(74)-C(73)	119.3(6)
C(53)-C(55)	1.536(8)			C(52)-C(47)-N(6)	118.5(5)	C(74)-C(75)-C(76)	120.4(6)
C(56)-C(57)	1.539(7)			C(47)-C(48)-C(49)	116.7(6)	C(75)-C(76)-C(71)	118.7(6)
C(56)-C(58)	1.551(8)			C(47)-C(48)-C(53)	122.7(5)	C(75)-C(76)-C(77)	135.7(5)
C(59)-C(60)	1.391(7)			C(49)-C(48)-C(53)	120.6(6)	C(71)-C(76)-C(77)	105.6(5)
C(59)-C(64)	1.412(7)			C(50)-C(49)-C(48)	120.4(6)	C(78)-C(77)-C(82)	118.9(6)

C(60)-C(61)	1.394(7)			C(51)-C(50)-C(49)	121.5(6)	C(78)-C(77)-C(76)	134.0(5)
C(60)-C(65)	1.526(7)			C(50)-C(51)-C(52)	120.5(6)	C(82)-C(77)-C(76)	107.1(5)
C(61)-C(62)	1.373(8)			C(51)-C(52)-C(47)	117.3(6)	C(79)-C(78)-C(77)	119.4(6)
C(62)-C(63)	1.368(8)			C(51)-C(52)-C(56)	119.6(6)	C(78)-C(79)-C(80)	120.2(6)
C(63)-C(64)	1.395(7)			C(47)-C(52)-C(56)	123.1(5)	C(81)-C(80)-C(79)	121.7(6)
C(64)-C(68)	1.516(7)			C(54)-C(53)-C(48)	112.9(6)	C(80)-C(81)-C(82)	118.4(5)
C(65)-C(66)	1.524(7)			C(54)-C(53)-C(55)	110.8(6)	N(10)-C(82)-C(81)	128.6(5)
C(65)-C(67)	1.534(8)			C(48)-C(53)-C(55)	110.0(5)	N(10)-C(82)-C(77)	110.0(5)
C(68)-C(70)	1.499(8)			C(52)-C(56)-C(57)	112.2(5)	C(81)-C(82)-C(77)	121.4(5)
C(68)-C(69)	1.514(7)			C(52)-C(56)-C(58)	111.2(5)		

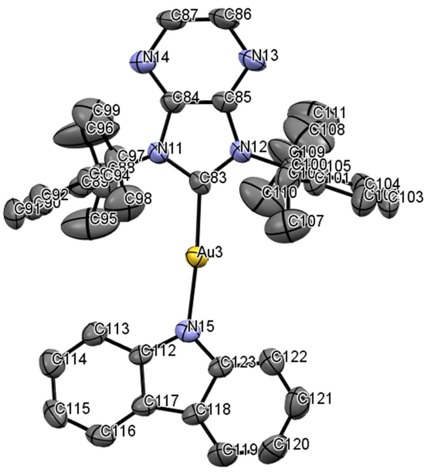

bond distances (Å)				bond angles (°)			
Au(3)-C(83)	1.977(5)	C(112)-C(113)	1.398(7)	C(83)-Au(3)-N(15)	174.72(18)	C(99)-C(97)-C(98)	110.8(5)
Au(3)-N(15)	2.009(4)	C(112)-C(117)	1.401(7)	C(83)-N(11)-C(84)	109.4(4)	C(105)-C(100)-C(101)	124.7(6)
N(11)-C(83)	1.369(6)	C(113)-C(114)	1.385(7)	C(83)-N(11)-C(88)	122.7(4)	C(105)-C(100)-N(12)	117.5(5)
N(11)-C(84)	1.397(6)	C(114)-C(115)	1.393(8)	C(84)-N(11)-C(88)	127.1(4)	C(101)-C(100)-N(12)	117.8(5)
N(11)-C(88)	1.449(6)	C(115)-C(116)	1.380(8)	C(83)-N(12)-C(85)	109.3(4)	C(102)-C(101)-C(100)	116.3(6)
N(12)-C(83)	1.365(6)	C(116)-C(117)	1.414(7)	C(83)-N(12)-C(100)	123.8(4)	C(102)-C(101)-C(106)	120.6(6)
N(12)-C(85)	1.389(6)	C(117)-C(118)	1.440(7)	C(85)-N(12)-C(100)	126.9(4)	C(100)-C(101)-C(106)	123.0(5)
N(12)-C(100)	1.442(6)	C(118)-C(119)	1.389(7)	C(85)-N(13)-C(86)	111.9(5)	C(103)-C(102)-C(101)	120.1(7)

N(13)-C(85)	1.330(6)	C(118)-C(123)	1.428(7)
N(13)-C(86)	1.341(7)	C(119)-C(120)	1.382(8)
N(14)-C(84)	1.330(6)	C(120)-C(121)	1.389(8)
N(14)-C(87)	1.343(6)	C(121)-C(122)	1.384(8)
N(15)-C(123)	1.381(6)	C(122)-C(123)	1.395(7)
N(15)-C(112)	1.404(6)		
C(84)-C(85)	1.383(7)		
C(86)-C(87)	1.401(8)		
C(88)-C(89)	1.399(7)		
C(88)-C(93)	1.412(7)		
C(89)-C(90)	1.403(7)		
C(89)-C(94)	1.527(7)		
C(90)-C(91)	1.371(8)		
C(91)-C(92)	1.379(8)		
C(92)-C(93)	1.386(7)		
C(93)-C(97)	1.517(7)		
C(94)-C(96)	1.469(9)		
C(94)-C(95)	1.505(9)		
C(97)-C(99)	1.525(8)		
C(97)-C(98)	1.529(8)		
C(100)-C(105)	1.395(8)		
C(100)-C(101)	1.404(7)		
C(101)-C(102)	1.398(8)		
C(101)-C(106)	1.515(8)		
C(102)-C(103)	1.379(9)		
C(103)-C(104)	1.360(10)		
C(104)-C(105)	1.399(9)		
C(105)-C(109)	1.532(9)		
C(106)-C(107)	1.506(8)		

C(84)-N(14)-C(87)	110.0(5)	C(104)-C(103)-C(102)	121.8(7)
C(123)-N(15)-C(112)	106.1(4)	C(103)-C(104)-C(105)	121.7(7)
C(123)-N(15)-Au(3)	130.4(4)	C(100)-C(105)-C(104)	115.3(6)
C(112)-N(15)-Au(3)	122.8(3)	C(100)-C(105)-C(109)	123.3(7)
N(12)-C(83)-N(11)	107.2(4)	C(104)-C(105)-C(109)	121.3(7)
N(12)-C(83)-Au(3)	129.4(4)	C(107)-C(106)-C(108)	111.3(6)
N(11)-C(83)-Au(3)	123.0(4)	C(107)-C(106)-C(101)	114.0(5)
N(14)-C(84)-C(85)	125.6(5)	C(108)-C(106)-C(101)	110.8(6)
N(14)-C(84)-N(11)	127.8(5)	C(110)-C(109)-C(111)	122.7(7)
C(85)-C(84)-N(11)	106.6(5)	C(110)-C(109)-C(105)	115.3(6)
N(13)-C(85)-C(84)	124.2(5)	C(111)-C(109)-C(105)	114.7(7)
N(13)-C(85)-N(12)	128.2(5)	C(113)-C(112)-C(117)	121.1(5)
C(84)-C(85)-N(12)	107.6(5)	C(113)-C(112)-N(15)	128.0(5)
N(13)-C(86)-C(87)	122.9(5)	C(117)-C(112)-N(15)	110.8(5)
N(14)-C(87)-C(86)	125.3(6)	C(114)-C(113)-C(112)	118.6(5)
C(89)-C(88)-C(93)	123.8(5)	C(113)-C(114)-C(115)	120.6(6)
C(89)-C(88)-N(11)	118.9(4)	C(116)-C(115)-C(114)	121.7(6)
C(93)-C(88)-N(11)	117.3(4)	C(115)-C(116)-C(117)	118.3(5)
C(88)-C(89)-C(90)	116.5(5)	C(112)-C(117)-C(116)	119.7(5)
C(88)-C(89)-C(94)	122.4(5)	C(112)-C(117)-C(118)	106.6(5)
C(90)-C(89)-C(94)	121.0(5)	C(116)-C(117)-C(118)	133.7(5)
C(91)-C(90)-C(89)	120.9(5)	C(119)-C(118)-C(123)	119.2(6)
C(90)-C(91)-C(92)	121.0(6)	C(119)-C(118)-C(117)	135.0(6)
C(91)-C(92)-C(93)	121.7(6)	C(123)-C(118)-C(117)	105.7(5)
C(92)-C(93)-C(88)	116.2(5)	C(120)-C(119)-C(118)	119.8(6)
C(92)-C(93)-C(97)	121.7(5)	C(119)-C(120)-C(121)	120.6(6)
C(88)-C(93)-C(97)	122.1(5)	C(122)-C(121)-C(120)	121.2(6)
C(96)-C(94)-C(95)	109.2(6)	C(121)-C(122)-C(123)	118.7(6)
C(96)-C(94)-C(89)	111.9(6)	N(15)-C(123)-C(122)	128.8(5)

C(106)-C(108)	1.512(8)			C(95)-C(94)-C(89)	112.8(6)	N(15)-C(123)-C(118)	110.8(5)
C(109)-C(110)	1.427(9)			C(93)-C(97)-C(99)	111.1(5)	C(122)-C(123)-C(118)	120.4(5)
C(109)-C(111)	1.440(8)			C(93)-C(97)-C(98)	111.6(5)		

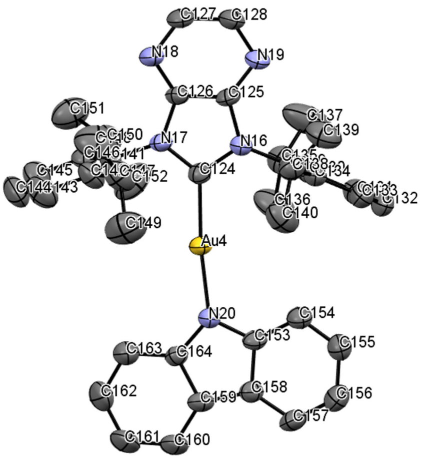

bond distances (Å)				bond angles (°)			
Au(4)-C(124)	1.974(5)	C(153)-C(154)	1.400(7)	C(124)-Au(4)-N(20)	172.34(19)	C(140)-C(138)-C(134)	111.3(5)
Au(4)-N(20)	1.999(4)	C(153)-C(158)	1.420(7)	C(124)-N(16)-C(125)	109.0(4)	C(142)-C(141)-C(146)	124.4(5)
N(16)-C(124)	1.369(6)	C(154)-C(155)	1.370(7)	C(124)-N(16)-C(129)	123.9(4)	C(142)-C(141)-N(17)	117.3(5)
N(16)-C(125)	1.395(6)	C(155)-C(156)	1.397(8)	C(125)-N(16)-C(129)	127.0(4)	C(146)-C(141)-N(17)	118.3(5)
N(16)-C(129)	1.442(6)	C(156)-C(157)	1.368(8)	C(124)-N(17)-C(126)	110.1(4)	C(141)-C(142)-C(143)	116.0(6)
N(17)-C(124)	1.358(6)	C(157)-C(158)	1.397(7)	C(124)-N(17)-C(141)	123.5(4)	C(141)-C(142)-C(147)	123.5(6)
N(17)-C(126)	1.384(6)	C(158)-C(159)	1.436(7)	C(126)-N(17)-C(141)	126.0(4)	C(143)-C(142)-C(147)	120.5(6)
N(17)-C(141)	1.456(6)	C(159)-C(160)	1.395(7)	C(126)-N(18)-C(127)	111.6(5)	C(144)-C(143)-C(142)	121.3(6)
N(18)-C(126)	1.317(6)	C(159)-C(164)	1.425(7)	C(125)-N(19)-C(128)	111.2(5)	C(143)-C(144)-C(145)	120.6(7)
N(18)-C(127)	1.345(6)	C(160)-C(161)	1.370(8)	C(153)-N(20)-C(164)	106.0(4)	C(144)-C(145)-C(146)	121.6(6)
N(19)-C(125)	1.328(6)	C(161)-C(162)	1.393(8)	C(153)-N(20)-Au(4)	121.8(3)	C(145)-C(146)-C(141)	116.1(5)
N(19)-C(128)	1.353(6)	C(162)-C(163)	1.381(8)	C(164)-N(20)-Au(4)	132.2(3)	C(145)-C(146)-C(150)	121.5(5)
N(20)-C(153)	1.387(6)	C(163)-C(164)	1.397(7)	N(17)-C(124)-N(16)	107.2(4)	C(141)-C(146)-C(150)	122.4(5)
N(20)-C(164)	1.392(6)			N(17)-C(124)-Au(4)	128.9(4)	C(142)-C(147)-C(148)	111.5(6)
C(125)-C(126)	1.394(7)			N(16)-C(124)-Au(4)	122.9(4)	C(142)-C(147)-C(149)	112.0(6)

C(127)-C(128)	1.395(7)	N(19)-C(125)-C(126)	124.9(5)	C(148)-C(147)-C(149)	109.6(5)
C(129)-C(134)	1.401(7)	N(19)-C(125)-N(16)	128.0(5)	C(146)-C(150)-C(151)	113.9(5)
C(129)-C(130)	1.408(7)	C(126)-C(125)-N(16)	107.0(4)	C(146)-C(150)-C(152)	111.2(5)
C(130)-C(131)	1.409(8)	N(18)-C(126)-N(17)	129.0(5)	C(151)-C(150)-C(152)	109.5(5)
C(130)-C(135)	1.505(8)	N(18)-C(126)-C(125)	124.4(5)	N(20)-C(153)-C(154)	127.9(5)
C(131)-C(132)	1.374(8)	N(17)-C(126)-C(125)	106.5(4)	N(20)-C(153)-C(158)	111.2(5)
C(132)-C(133)	1.378(8)	N(18)-C(127)-C(128)	124.3(5)	C(154)-C(153)-C(158)	120.9(5)
C(133)-C(134)	1.393(7)	N(19)-C(128)-C(127)	123.4(5)	C(155)-C(154)-C(153)	118.1(5)
C(134)-C(138)	1.534(7)	C(134)-C(129)-C(130)	123.4(5)	C(154)-C(155)-C(156)	121.6(6)
C(135)-C(136)	1.526(7)	C(134)-C(129)-N(16)	118.1(4)	C(157)-C(156)-C(155)	120.7(6)
C(135)-C(137)	1.529(8)	C(130)-C(129)-N(16)	118.5(5)	C(156)-C(157)-C(158)	119.7(6)
C(138)-C(139)	1.529(8)	C(129)-C(130)-C(131)	115.8(6)	C(157)-C(158)-C(153)	119.0(5)
C(138)-C(140)	1.533(7)	C(129)-C(130)-C(135)	122.7(5)	C(157)-C(158)-C(159)	135.0(5)
C(141)-C(142)	1.393(7)	C(131)-C(130)-C(135)	121.5(5)	C(153)-C(158)-C(159)	106.0(5)
C(141)-C(146)	1.404(7)	C(132)-C(131)-C(130)	121.6(6)	C(160)-C(159)-C(164)	119.8(5)
C(142)-C(143)	1.405(8)	C(131)-C(132)-C(133)	121.0(6)	C(160)-C(159)-C(158)	134.3(5)
C(142)-C(147)	1.514(8)	C(132)-C(133)-C(134)	120.5(6)	C(164)-C(159)-C(158)	105.9(5)
C(143)-C(144)	1.367(9)	C(133)-C(134)-C(129)	117.7(5)	C(161)-C(160)-C(159)	119.1(6)
C(144)-C(145)	1.383(8)	C(133)-C(134)-C(138)	120.5(5)	C(160)-C(161)-C(162)	121.1(6)
C(145)-C(146)	1.385(8)	C(129)-C(134)-C(138)	121.8(5)	C(163)-C(162)-C(161)	121.5(6)
C(146)-C(150)	1.521(8)	C(130)-C(135)-C(136)	112.5(5)	C(162)-C(163)-C(164)	118.1(6)
C(147)-C(148)	1.544(9)	C(130)-C(135)-C(137)	112.0(6)	N(20)-C(164)-C(163)	128.8(5)
C(147)-C(149)	1.544(8)	C(136)-C(135)-C(137)	109.1(5)	N(20)-C(164)-C(159)	110.8(5)
C(150)-C(151)	1.538(8)	C(139)-C(138)-C(140)	111.8(5)	C(163)-C(164)-C(159)	120.3(5)
C(150)-C(152)	1.542(8)	C(139)-C(138)-C(134)	111.0(5)		

Table S6. Crystallographic data for [Au(^{Dipp}PZI)(DPA)]

	[Au(^{Dipp}PZI)(DPA)]
Empirical formula	$C_{41}H_{46}AuN_5$
Formula weight	805.79
Temperature (K)	223(2)
Wavelength (Å)	0.71073
Crystal system	orthorhombic
Space group	*Pbca*
Unit cell dimensions	
a (Å)	19.901(17)
b (Å)	16.114(16)
c (Å)	24.04(3)
α (°)	90
β (°)	90
γ (°)	90
Volume (Å^3)	7709(13)
Z	8
Calculated density (g cm^{-3})	1.389
Absorption coefficient (mm^{-1})	3.850
F(000)	3248
Crystal size (mm^3)	0.132 × 0.116 × 0.020
Theta range for data collection	1.834 to 28.229°.
Index ranges	$-22 \leq h \leq 26$, $-21 \leq k \leq 20$, $-31 \leq l \leq 31$
Reflections collected	56503
Independent reflections [*R*(int)]	9215 [0.1665]
Completeness to theta = 25.242°	100.00%
Absorption correction	Semi-empirical from equivalents
Max. and min. transmission	0.7457 and 0.6039
Refinement method	Full-matrix least-squares on F^2
Data / restraints / parameters	9215 / 12 / 432
Goodness-of-fit on F^2	1.009
Final *R* indices [I > 2sigma(*I*)]	$R_1 = 0.0554$, $wR_2 = 0.1202$
R indices (all data)	$R_1 = 0.1633$, $wR_2 = 0.1757$
Extinction coefficient	n/a
Largest diff. peak and hole (e Å^{-3})	2.634 and -1.372

Table S7. Selected bond distances and bond angles of [Au(^{Dipp}PZI)(DPA)]

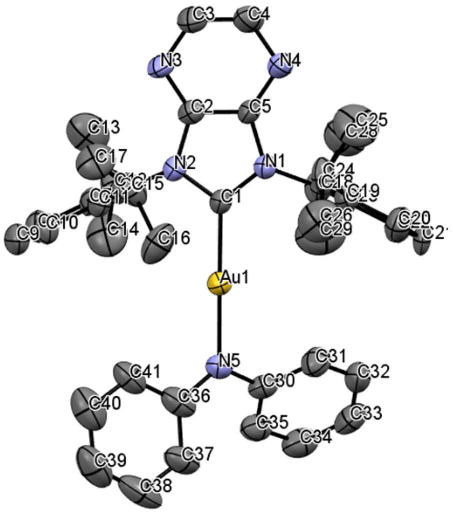

bond distances (Å)				bond angles (°)			
Au(1)-C(1)	2.000(9)	C(27)-C(29)	1.521(14)	C(1)-Au(1)-N(5)	176.7(3)	C(11)-C(15)-C(16)	110.2(8)
Au(1)-N(5)	2.063(7)	C(27)-C(28)	1.551(14)	C(1)-N(1)-C(5)	111.5(7)	C(11)-C(15)-C(17)	112.1(9)
N(1)-C(1)	1.378(10)	C(30)-C(35)	1.410(12)	C(1)-N(1)-C(18)	121.5(7)	C(16)-C(15)-C(17)	108.9(8)
N(1)-C(5)	1.396(10)	C(30)-C(31)	1.423(13)	C(5)-N(1)-C(18)	126.7(7)	C(23)-C(18)-C(19)	123.8(8)
N(1)-C(18)	1.454(9)	C(31)-C(32)	1.362(13)	C(1)-N(2)-C(2)	109.7(7)	C(23)-C(18)-N(1)	117.6(7)
N(2)-C(1)	1.380(10)	C(32)-C(33)	1.407(14)	C(1)-N(2)-C(6)	121.8(7)	C(19)-C(18)-N(1)	118.4(8)
N(2)-C(2)	1.423(10)	C(33)-C(34)	1.368(14)	C(2)-N(2)-C(6)	128.5(7)	C(20)-C(19)-C(18)	117.9(9)
N(2)-C(6)	1.452(10)	C(34)-C(35)	1.395(13)	C(2)-N(3)-C(3)	112.4(8)	C(20)-C(19)-C(24)	119.7(9)
N(3)-C(2)	1.348(10)	C(36)-C(41)	1.409(14)	C(5)-N(4)-C(4)	111.6(8)	C(18)-C(19)-C(24)	122.2(8)
N(3)-C(3)	1.362(11)	C(36)-C(37)	1.418(14)	C(36)-N(5)-C(30)	121.6(8)	C(21)-C(20)-C(19)	119.0(10)
N(4)-C(5)	1.358(10)	C(37)-C(38)	1.415(15)	C(36)-N(5)-Au(1)	122.5(7)	C(20)-C(21)-C(22)	123.8(9)
N(4)-C(4)	1.367(11)	C(38)-C(39)	1.368(17)	C(30)-N(5)-Au(1)	115.9(6)	C(21)-C(22)-C(23)	118.7(8)
N(5)-C(36)	1.412(12)	C(39)-C(40)	1.416(17)	N(1)-C(1)-N(2)	105.8(7)	C(18)-C(23)-C(22)	116.7(8)
N(5)-C(30)	1.421(12)	C(40)-C(41)	1.397(14)	N(1)-C(1)-Au(1)	128.1(6)	C(18)-C(23)-C(27)	122.8(8)
C(2)-C(5)	1.401(11)			N(2)-C(1)-Au(1)	125.7(6)	C(22)-C(23)-C(27)	120.5(8)
C(3)-C(4)	1.375(13)			N(3)-C(2)-C(5)	123.1(8)	C(19)-C(24)-C(25)	110.6(9)
C(6)-C(7)	1.425(12)			N(3)-C(2)-N(2)	130.1(8)	C(19)-C(24)-C(26)	114.0(9)
C(6)-C(11)	1.427(12)			C(5)-C(2)-N(2)	106.8(7)	C(25)-C(24)-C(26)	109.8(10)
C(7)-C(8)	1.399(12)			N(3)-C(3)-C(4)	124.5(9)	C(29)-C(27)-C(23)	112.8(9)
C(7)-C(12)	1.555(13)			N(4)-C(4)-C(3)	123.8(9)	C(29)-C(27)-C(28)	108.1(10)
C(8)-C(9)	1.390(14)			N(4)-C(5)-N(1)	129.2(7)	C(23)-C(27)-C(28)	111.2(8)
C(9)-C(10)	1.399(13)			N(4)-C(5)-C(2)	124.5(8)	C(35)-C(30)-N(5)	123.2(9)
C(10)-C(11)	1.415(12)			N(1)-C(5)-C(2)	106.2(7)	C(35)-C(30)-C(31)	118.2(9)
C(11)-C(15)	1.538(13)			C(7)-C(6)-C(11)	123.8(8)	N(5)-C(30)-C(31)	118.6(8)
C(12)-C(13)	1.550(15)			C(7)-C(6)-N(2)	118.8(8)	C(32)-C(31)-C(30)	119.7(10)
C(12)-C(14)	1.565(14)			C(11)-C(6)-N(2)	117.4(8)	C(31)-C(32)-C(33)	121.9(10)
C(15)-C(16)	1.569(13)			C(8)-C(7)-C(6)	116.8(9)	C(34)-C(33)-C(32)	118.8(11)
C(15)-C(17)	1.582(13)			C(8)-C(7)-C(12)	122.0(9)	C(33)-C(34)-C(35)	121.0(11)
C(18)-C(23)	1.399(11)			C(6)-C(7)-C(12)	121.2(8)	C(34)-C(35)-C(30)	120.3(10)
C(18)-C(19)	1.427(12)			C(9)-C(8)-C(7)	121.2(10)	C(41)-C(36)-N(5)	117.2(9)
C(19)-C(20)	1.404(12)			C(8)-C(9)-C(10)	121.2(10)	C(41)-C(36)-C(37)	116.2(10)
C(19)-C(24)	1.511(13)			C(9)-C(10)-C(11)	121.2(9)	N(5)-C(36)-C(37)	126.6(10)
C(20)-C(21)	1.372(13)			C(10)-C(11)-C(6)	115.8(9)	C(38)-C(37)-C(36)	122.4(12)
C(21)-C(22)	1.410(12)			C(10)-C(11)-C(15)	122.4(9)	C(39)-C(38)-C(37)	119.9(14)

C(22)-C(23)	1.431(11)	C(6)-C(11)-C(15)	121.8(8)	C(38)-C(39)-C(40)	119.2(13)
C(23)-C(27)	1.538(12)	C(13)-C(12)-C(7)	111.9(8)	C(41)-C(40)-C(39)	120.9(13)
C(24)-C(25)	1.540(15)	C(13)-C(12)-C(14)	110.5(9)	C(40)-C(41)-C(36)	121.3(12)
C(24)-C(26)	1.575(14)	C(7)-C(12)-C(14)	110.2(9)		

Table S8. Crystallographic data for [Au(^{Dipp}PZI)(DPAC)]

	[Au(^{Dipp}PZI)(DPAC)]
Empirical formula	$C_{54}H_{54}AuN_5$
Formula weight	969.99
Temperature (K)	223(2)
Wavelength (Å)	0.71073
Crystal system	monoclinic
Space group	$P2_1/c$
Unit cell dimensions	
a (Å)	14.645(3)
b (Å)	14.248(3)
c (Å)	21.785(5)
α (°)	90
β (°)	98.474(7)
γ (°)	90
Volume (Å^3)	4496.2(16)
Z	4
Calculated density (g cm^{-3})	1.433
Absorption coefficient (mm^{-1})	3.314
F(000)	1968
Crystal size (mm^3)	0.100 × 0.067 × 0.047
Theta range for data collection	1.890 to 28.404°.
Index ranges	$-17 \le h \le 19$, $-18 \le k \le 18$, $-28 \le l \le 28$
Reflections collected	69346
Independent reflections [*R*(int)]	11156 [0.1722]
Completeness to theta = 25.242°	100.00%
Absorption correction	Semi-empirical from equivalents
Max. and min. transmission	0.7457 and 0.6369
Refinement method	Full-matrix least-squares on F^2
Data / restraints / parameters	11156 / 0 / 549
Goodness-of-fit on F^2	0.998
Final *R* indices [I > 2sigma(*I*)]	$R_1 = 0.0512$, $wR_2 = 0.0882$
R indices (all data)	$R_1 = 0.1305$, $wR_2 = 0.1150$
Extinction coefficient	n/a
Largest diff. peak and hole (e Å^{-3})	1.649 and -0.810

Table S9. Selected bond distances and bond angles of [Au(^{Dipp}PZI)(DPAC)]

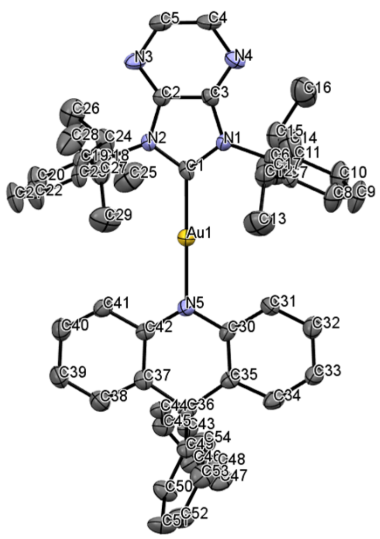

bond distances (Å)				bond angles (°)			
Au(1)-C(1)	1.956(6)	C(37)-C(42)	1.413(8)	C(1)-Au(1)-N(5)	176.6(2)	C(22)-C(23)-C(27)	120.1(6)
Au(1)-N(5)	2.036(5)	C(38)-C(39)	1.380(8)	C(1)-N(1)-C(3)	110.3(5)	C(18)-C(23)-C(27)	122.9(6)
N(1)-C(1)	1.375(7)	C(39)-C(40)	1.382(8)	C(1)-N(1)-C(6)	122.8(5)	C(25)-C(24)-C(26)	110.2(6)
N(1)-C(3)	1.387(7)	C(40)-C(41)	1.374(9)	C(3)-N(1)-C(6)	126.9(5)	C(25)-C(24)-C(19)	112.5(6)
N(1)-C(6)	1.445(7)	C(41)-C(42)	1.409(8)	C(1)-N(2)-C(2)	110.7(5)	C(26)-C(24)-C(19)	112.9(6)
N(2)-C(1)	1.375(7)	C(43)-C(48)	1.395(8)	C(1)-N(2)-C(18)	122.1(5)	C(28)-C(27)-C(29)	111.6(6)
N(2)-C(2)	1.386(7)	C(43)-C(44)	1.396(8)	C(2)-N(2)-C(18)	127.2(5)	C(28)-C(27)-C(23)	109.9(6)
N(2)-C(18)	1.462(7)	C(44)-C(45)	1.379(8)	C(2)-N(3)-C(5)	111.7(6)	C(29)-C(27)-C(23)	111.7(6)
N(3)-C(2)	1.318(7)	C(45)-C(46)	1.382(8)	C(3)-N(4)-C(4)	112.7(6)	N(5)-C(30)-C(35)	123.4(6)
N(3)-C(5)	1.357(7)	C(46)-C(47)	1.372(9)	C(30)-N(5)-C(42)	118.6(5)	N(5)-C(30)-C(31)	118.6(6)
N(4)-C(3)	1.324(7)	C(47)-C(48)	1.377(8)	C(30)-N(5)-Au(1)	120.5(4)	C(35)-C(30)-C(31)	118.0(6)
N(4)-C(4)	1.347(7)	C(49)-C(54)	1.381(8)	C(42)-N(5)-Au(1)	120.4(4)	C(32)-C(31)-C(30)	122.3(6)
N(5)-C(30)	1.390(7)	C(49)-C(50)	1.387(8)	N(2)-C(1)-N(1)	106.0(5)	C(31)-C(32)-C(33)	119.2(7)
N(5)-C(42)	1.393(7)	C(50)-C(51)	1.394(8)	N(2)-C(1)-Au(1)	126.7(4)	C(34)-C(33)-C(32)	119.1(6)
C(2)-C(3)	1.405(8)	C(51)-C(52)	1.381(9)	N(1)-C(1)-Au(1)	126.9(4)	C(33)-C(34)-C(35)	123.6(6)
C(4)-C(5)	1.400(9)	C(52)-C(53)	1.391(9)	N(3)-C(2)-N(2)	128.4(6)	C(30)-C(35)-C(34)	117.9(6)
C(6)-C(7)	1.388(8)	C(53)-C(54)	1.394(9)	N(3)-C(2)-C(3)	125.3(6)	C(30)-C(35)-C(36)	121.2(5)
C(6)-C(11)	1.399(8)			N(2)-C(2)-C(3)	106.3(5)	C(34)-C(35)-C(36)	120.7(5)
C(7)-C(8)	1.404(8)			N(4)-C(3)-N(1)	129.9(6)	C(35)-C(36)-C(43)	107.6(5)
C(7)-C(12)	1.533(9)			N(4)-C(3)-C(2)	123.2(6)	C(35)-C(36)-C(37)	109.9(5)
C(8)-C(9)	1.365(10)			N(1)-C(3)-C(2)	106.8(5)	C(43)-C(36)-C(37)	109.9(5)
C(9)-C(10)	1.365(10)			N(4)-C(4)-C(5)	123.6(6)	C(35)-C(36)-C(49)	111.0(5)

C(10)-C(11)	1.409(9)	N(3)-C(5)-C(4)	123.4(6)	C(43)-C(36)-C(49)	110.9(5)
C(11)-C(15)	1.514(8)	C(7)-C(6)-C(11)	123.4(6)	C(37)-C(36)-C(49)	107.5(5)
C(12)-C(14)	1.514(9)	C(7)-C(6)-N(1)	118.0(6)	C(38)-C(37)-C(42)	118.2(6)
C(12)-C(13)	1.534(9)	C(11)-C(6)-N(1)	118.6(5)	C(38)-C(37)-C(36)	120.5(6)
C(15)-C(17)	1.504(9)	C(6)-C(7)-C(8)	117.2(6)	C(42)-C(37)-C(36)	121.2(6)
C(15)-C(16)	1.546(9)	C(6)-C(7)-C(12)	123.5(6)	C(39)-C(38)-C(37)	124.0(6)
C(18)-C(19)	1.392(8)	C(8)-C(7)-C(12)	119.2(6)	C(38)-C(39)-C(40)	117.6(7)
C(18)-C(23)	1.406(8)	C(9)-C(8)-C(7)	120.3(6)	C(41)-C(40)-C(39)	120.1(7)
C(19)-C(20)	1.394(8)	C(8)-C(9)-C(10)	122.0(7)	C(40)-C(41)-C(42)	123.3(6)
C(19)-C(24)	1.534(8)	C(9)-C(10)-C(11)	120.5(7)	N(5)-C(42)-C(41)	120.4(5)
C(20)-C(21)	1.374(9)	C(6)-C(11)-C(10)	116.6(6)	N(5)-C(42)-C(37)	122.8(5)
C(21)-C(22)	1.386(9)	C(6)-C(11)-C(15)	123.9(6)	C(41)-C(42)-C(37)	116.8(6)
C(22)-C(23)	1.393(8)	C(10)-C(11)-C(15)	119.5(6)	C(48)-C(43)-C(44)	116.4(5)
C(23)-C(27)	1.534(8)	C(14)-C(12)-C(7)	109.1(6)	C(48)-C(43)-C(36)	121.6(5)
C(24)-C(25)	1.519(8)	C(14)-C(12)-C(13)	111.9(6)	C(44)-C(43)-C(36)	121.9(5)
C(24)-C(26)	1.523(8)	C(7)-C(12)-C(13)	113.5(6)	C(45)-C(44)-C(43)	121.4(6)
C(27)-C(28)	1.526(9)	C(17)-C(15)-C(11)	112.6(6)	C(44)-C(45)-C(46)	120.7(6)
C(27)-C(29)	1.527(9)	C(17)-C(15)-C(16)	110.7(7)	C(47)-C(46)-C(45)	119.0(6)
C(30)-C(35)	1.404(8)	C(11)-C(15)-C(16)	111.1(6)	C(46)-C(47)-C(48)	120.2(6)
C(30)-C(31)	1.432(8)	C(19)-C(18)-C(23)	123.2(6)	C(47)-C(48)-C(43)	122.3(6)
C(31)-C(32)	1.378(8)	C(19)-C(18)-N(2)	118.2(6)	C(54)-C(49)-C(50)	117.0(6)
C(32)-C(33)	1.396(8)	C(23)-C(18)-N(2)	118.6(5)	C(54)-C(49)-C(36)	120.0(6)
C(33)-C(34)	1.371(8)	C(18)-C(19)-C(20)	116.9(6)	C(50)-C(49)-C(36)	122.9(5)
C(34)-C(35)	1.406(8)	C(18)-C(19)-C(24)	122.7(6)	C(49)-C(50)-C(51)	122.6(6)
C(35)-C(36)	1.534(8)	C(20)-C(19)-C(24)	120.4(6)	C(52)-C(51)-C(50)	119.3(7)
C(36)-C(43)	1.541(8)	C(21)-C(20)-C(19)	121.7(7)	C(51)-C(52)-C(53)	119.2(6)
C(36)-C(37)	1.546(8)	C(20)-C(21)-C(22)	120.2(7)	C(52)-C(53)-C(54)	120.3(6)
C(36)-C(49)	1.551(8)	C(21)-C(22)-C(23)	120.9(7)	C(49)-C(54)-C(53)	121.5(6)
C(37)-C(38)	1.401(8)	C(22)-C(23)-C(18)	117.1(6)		

Table S10. Crystallographic data for [Au(^{Dipp}PZI)(DMAC)]

	[Au(^{Dipp}PZI)(DMAC)]
Empirical formula	$C_{44}H_{50}AuN_5$
Formula weight	845.85
Temperature (K)	223(2)
Wavelength (Å)	0.71073
Crystal system	orthorhombic
Space group	$P2_12_12_1$
Unit cell dimensions	
a (Å)	16.035(9)
b (Å)	20.631(10)
c (Å)	23.907(11)
α (°)	90
β (°)	90
γ (°)	90
Volume (Å^3)	7909(7)
Z	8
Calculated density (g cm^{-3})	1.421
Absorption coefficient (mm^{-1})	3.756
F(000)	3424
Crystal size (mm^3)	0.119 × 0.086 × 0.080
Theta range for data collection	1.820 to 29.275°.
Index ranges	$-21 \le h \le 21$, $-27 \le k \le 27$, $-30 \le l \le 32$
Reflections collected	145224
Independent reflections [*R*(int)]	20215 [0.1405]
Completeness to theta = 25.242°	99.90%
Absorption correction	Semi-empirical from equivalents
Max. and min. transmission	0.7457 and 0.6783
Refinement method	Full-matrix least-squares on F^2
Data / restraints / parameters	20215 / 0 / 920
Goodness-of-fit on F^2	1.005
Final *R* indices [I > 2sigma(*I*)]	R_1 = 0.0449, wR_2 = 0.0752
R indices (all data)	R_1 = 0.1221, wR_2 = 0.0984
Extinction coefficient	n/a
Largest diff. peak and hole (e Å^{-3})	1.508 and -1.656

Table S11. Selected bond distances and bond angles of [Au(^{Dipp}PZI)(DMAC)]

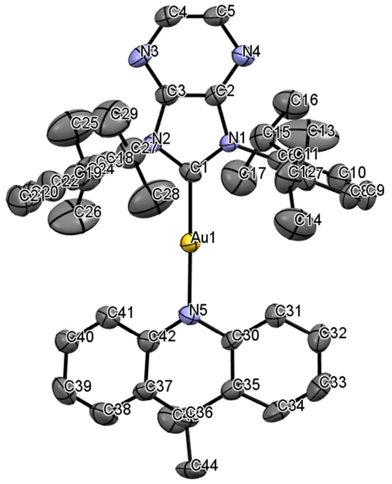

bond distances (Å)				bond angles (°)			
Au(1)-C(1)	1.968(10)	C(31)-C(32)	1.388(13)	C(1)-Au(1)-N(5)	178.7(4)	C(23)-C(18)-N(2)	115.3(10)
Au(1)-N(5)	2.027(8)	C(32)-C(33)	1.387(16)	C(1)-N(1)-C(2)	110.5(8)	C(20)-C(19)-C(18)	115.1(12)
N(1)-C(1)	1.363(11)	C(33)-C(34)	1.346(17)	C(1)-N(1)-C(6)	123.6(8)	C(20)-C(19)-C(24)	122.8(13)
N(1)-C(2)	1.384(12)	C(34)-C(35)	1.389(14)	C(2)-N(1)-C(6)	125.9(9)	C(18)-C(19)-C(24)	122.0(11)
N(1)-C(6)	1.472(11)	C(35)-C(36)	1.528(16)	C(1)-N(2)-C(3)	110.5(8)	C(21)-C(20)-C(19)	122.9(15)
N(2)-C(1)	1.359(11)	C(36)-C(37)	1.527(15)	C(1)-N(2)-C(18)	121.8(8)	C(20)-C(21)-C(22)	119.6(14)
N(2)-C(3)	1.396(13)	C(36)-C(43)	1.535(15)	C(3)-N(2)-C(18)	127.6(8)	C(21)-C(22)-C(23)	122.1(14)
N(2)-C(18)	1.455(12)	C(36)-C(44)	1.550(14)	C(3)-N(3)-C(4)	110.8(10)	C(22)-C(23)-C(18)	115.2(13)
N(3)-C(3)	1.332(13)	C(37)-C(38)	1.375(14)	C(2)-N(4)-C(5)	111.0(10)	C(22)-C(23)-C(27)	120.3(12)
N(3)-C(4)	1.341(14)	C(37)-C(42)	1.415(13)	C(42)-N(5)-C(30)	117.8(8)	C(18)-C(23)-C(27)	124.4(11)
N(4)-C(2)	1.333(13)	C(38)-C(39)	1.386(16)	C(42)-N(5)-Au(1)	121.3(6)	C(25)-C(24)-C(26)	114.7(14)
N(4)-C(5)	1.347(13)	C(39)-C(40)	1.388(17)	C(30)-N(5)-Au(1)	120.2(7)	C(25)-C(24)-C(19)	110.1(12)
N(5)-C(42)	1.384(12)	C(40)-C(41)	1.375(15)	N(2)-C(1)-N(1)	106.4(8)	C(26)-C(24)-C(19)	114.1(14)
N(5)-C(30)	1.401(12)	C(41)-C(42)	1.417(14)	N(2)-C(1)-Au(1)	127.6(7)	C(23)-C(27)-C(29)	114.7(11)
C(2)-C(3)	1.401(14)			N(1)-C(1)-Au(1)	126.0(7)	C(23)-C(27)-C(28)	109.5(13)
C(4)-C(5)	1.365(15)			N(4)-C(2)-N(1)	129.4(9)	C(29)-C(27)-C(28)	112.3(12)
C(6)-C(11)	1.392(14)			N(4)-C(2)-C(3)	123.9(10)	C(31)-C(30)-N(5)	119.0(9)
C(6)-C(7)	1.401(14)			N(1)-C(2)-C(3)	106.6(10)	C(31)-C(30)-C(35)	119.1(9)
C(7)-C(8)	1.393(13)			N(3)-C(3)-N(2)	129.5(10)	N(5)-C(30)-C(35)	121.9(10)

C(7)-C(12)	1.486(15)	N(3)-C(3)-C(2)	124.5(11)	C(32)-C(31)-C(30)	121.7(11)
C(8)-C(9)	1.381(15)	N(2)-C(3)-C(2)	105.9(9)	C(33)-C(32)-C(31)	118.9(12)
C(9)-C(10)	1.341(16)	N(3)-C(4)-C(5)	125.0(11)	C(34)-C(33)-C(32)	119.1(11)
C(10)-C(11)	1.400(14)	N(4)-C(5)-C(4)	124.7(12)	C(33)-C(34)-C(35)	124.4(13)
C(11)-C(15)	1.508(15)	C(11)-C(6)-C(7)	124.9(9)	C(34)-C(35)-C(30)	116.7(11)
C(12)-C(13)	1.469(19)	C(11)-C(6)-N(1)	117.7(9)	C(34)-C(35)-C(36)	122.3(11)
C(12)-C(14)	1.525(16)	C(7)-C(6)-N(1)	117.4(9)	C(30)-C(35)-C(36)	120.9(9)
C(15)-C(16)	1.518(15)	C(8)-C(7)-C(6)	115.4(10)	C(37)-C(36)-C(35)	109.6(9)
C(15)-C(17)	1.527(18)	C(8)-C(7)-C(12)	121.5(10)	C(37)-C(36)-C(43)	107.4(9)
C(18)-C(19)	1.393(15)	C(6)-C(7)-C(12)	123.0(9)	C(35)-C(36)-C(43)	109.0(9)
C(18)-C(23)	1.401(14)	C(9)-C(8)-C(7)	120.7(11)	C(37)-C(36)-C(44)	111.2(9)
C(19)-C(20)	1.389(17)	C(10)-C(9)-C(8)	122.1(11)	C(35)-C(36)-C(44)	111.1(10)
C(19)-C(24)	1.501(17)	C(9)-C(10)-C(11)	121.1(12)	C(43)-C(36)-C(44)	108.6(10)
C(20)-C(21)	1.367(19)	C(6)-C(11)-C(10)	115.8(11)	C(38)-C(37)-C(42)	117.5(10)
C(21)-C(22)	1.369(19)	C(6)-C(11)-C(15)	122.7(9)	C(38)-C(37)-C(36)	122.5(10)
C(21)-H(21)	0.94	C(10)-C(11)-C(15)	121.5(11)	C(42)-C(37)-C(36)	119.9(10)
C(22)-C(23)	1.399(15)	C(13)-C(12)-C(7)	114.1(12)	C(37)-C(38)-C(39)	124.5(12)
C(23)-C(27)	1.493(17)	C(13)-C(12)-C(14)	112.4(12)	C(38)-C(39)-C(40)	117.7(12)
C(24)-C(25)	1.458(18)	C(7)-C(12)-C(14)	113.0(12)	C(41)-C(40)-C(39)	120.2(12)
C(24)-C(26)	1.496(19)	C(11)-C(15)-C(16)	113.5(10)	C(40)-C(41)-C(42)	121.5(11)
C(27)-C(29)	1.512(17)	C(11)-C(15)-C(17)	109.9(11)	N(5)-C(42)-C(37)	123.1(9)
C(27)-C(28)	1.539(17)	C(16)-C(15)-C(17)	110.2(11)	N(5)-C(42)-C(41)	118.4(10)
C(30)-C(31)	1.391(14)	C(19)-C(18)-C(23)	125.0(11)	C(37)-C(42)-C(41)	118.5(10)
C(30)-C(35)	1.413(15)	C(19)-C(18)-N(2)	119.6(10)		

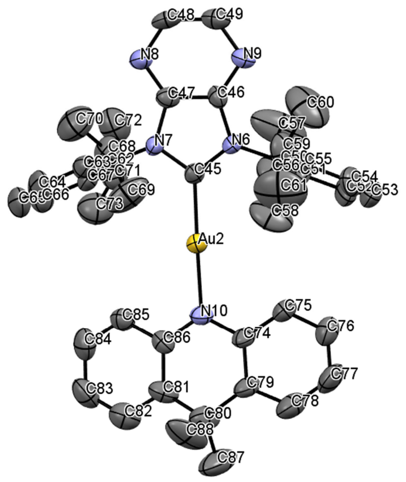

bond distances (Å)				bond angles (°)			
Au(2)-C(45)	1.972(10)	C(76)-C(77)	1.354(17)	C(45)-Au(2)-N(10)	178.6(4)	C(67)-C(62)-N(7)	117.7(10)
Au(2)-N(10)	2.035(8)	C(77)-C(78)	1.366(18)	C(45)-N(6)-C(46)	110.1(8)	C(62)-C(63)-C(64)	117.9(12)
N(6)-C(45)	1.355(12)	C(78)-C(79)	1.416(16)	C(45)-N(6)-C(50)	123.3(9)	C(62)-C(63)-C(68)	120.5(11)
N(6)-C(46)	1.396(13)	C(79)-C(80)	1.493(18)	C(46)-N(6)-C(50)	126.6(9)	C(64)-C(63)-C(68)	121.5(12)
N(6)-C(50)	1.455(12)	C(80)-C(81)	1.529(17)	C(45)-N(7)-C(47)	109.9(9)	C(65)-C(64)-C(63)	120.0(13)
N(7)-C(45)	1.377(11)	C(80)-C(87)	1.541(17)	C(45)-N(7)-C(62)	122.9(9)	C(64)-C(65)-C(66)	122.6(13)
N(7)-C(47)	1.395(14)	C(80)-C(88)	1.568(18)	C(47)-N(7)-C(62)	127.0(8)	C(65)-C(66)-C(67)	120.0(13)
N(7)-C(62)	1.451(13)	C(81)-C(82)	1.376(15)	C(47)-N(8)-C(48)	111.7(10)	C(66)-C(67)-C(62)	115.9(12)
N(8)-C(47)	1.318(13)	C(81)-C(86)	1.412(14)	C(46)-N(9)-C(49)	110.9(10)	C(66)-C(67)-C(71)	122.6(11)
N(8)-C(48)	1.343(15)	C(82)-C(83)	1.388(17)	C(86)-N(10)-C(74)	118.9(9)	C(62)-C(67)-C(71)	121.5(10)
N(9)-C(46)	1.318(14)	C(83)-C(84)	1.384(18)	C(86)-N(10)-Au(2)	120.2(6)	C(63)-C(68)-C(69)	109.6(12)
N(9)-C(49)	1.353(14)	C(84)-C(85)	1.356(16)	C(74)-N(10)-Au(2)	120.8(7)	C(63)-C(68)-C(70)	113.1(10)
N(10)-C(86)	1.388(12)	C(85)-C(86)	1.421(14)	N(6)-C(45)-N(7)	107.0(9)	C(69)-C(68)-C(70)	109.5(11)
N(10)-C(74)	1.393(12)			N(6)-C(45)-Au(2)	125.4(7)	C(73)-C(71)-C(67)	112.2(11)
C(46)-C(47)	1.403(14)			N(7)-C(45)-Au(2)	127.6(8)	C(73)-C(71)-C(72)	108.8(11)
C(48)-C(49)	1.382(17)			N(9)-C(46)-N(6)	128.5(10)	C(67)-C(71)-C(72)	112.5(11)
C(50)-C(55)	1.375(15)			N(9)-C(46)-C(47)	124.6(11)	N(10)-C(74)-C(75)	118.7(10)
C(50)-C(51)	1.394(15)			N(6)-C(46)-C(47)	106.9(10)	N(10)-C(74)-C(79)	122.3(11)
C(51)-C(52)	1.397(14)			N(8)-C(47)-N(7)	129.3(11)	C(75)-C(74)-C(79)	119.0(10)

C(51)-C(56)	1.484(16)	N(8)-C(47)-C(46)	124.2(12)	C(76)-C(75)-C(74)	121.0(11)
C(52)-C(53)	1.385(17)	N(7)-C(47)-C(46)	106.1(10)	C(77)-C(76)-C(75)	120.8(13)
C(53)-C(54)	1.348(18)	N(8)-C(48)-C(49)	123.8(11)	C(76)-C(77)-C(78)	118.8(12)
C(54)-C(55)	1.418(17)	N(9)-C(49)-C(48)	124.5(12)	C(77)-C(78)-C(79)	123.5(13)
C(55)-C(59)	1.498(17)	C(55)-C(50)-C(51)	124.9(10)	C(74)-C(79)-C(78)	116.8(12)
C(56)-C(57)	1.429(17)	C(55)-C(50)-N(6)	118.2(10)	C(74)-C(79)-C(80)	121.5(11)
C(56)-C(58)	1.526(15)	C(51)-C(50)-N(6)	116.8(9)	C(78)-C(79)-C(80)	121.5(12)
C(59)-C(60)	1.493(18)	C(50)-C(51)-C(52)	116.5(11)	C(79)-C(80)-C(81)	111.9(11)
C(59)-C(61)	1.50(2)	C(50)-C(51)-C(56)	122.4(10)	C(79)-C(80)-C(87)	109.9(12)
C(62)-C(63)	1.384(15)	C(52)-C(51)-C(56)	121.1(11)	C(81)-C(80)-C(87)	110.0(10)
C(62)-C(67)	1.412(14)	C(53)-C(52)-C(51)	120.3(12)	C(79)-C(80)-C(88)	109.7(11)
C(63)-C(64)	1.391(16)	C(54)-C(53)-C(52)	121.0(12)	C(81)-C(80)-C(88)	106.3(12)
C(63)-C(68)	1.510(16)	C(53)-C(54)-C(55)	121.8(13)	C(87)-C(80)-C(88)	109.0(13)
C(64)-C(65)	1.353(17)	C(50)-C(55)-C(54)	115.4(13)	C(82)-C(81)-C(86)	118.8(12)
C(65)-C(66)	1.385(18)	C(50)-C(55)-C(59)	123.8(11)	C(82)-C(81)-C(80)	121.4(12)
C(66)-C(67)	1.406(15)	C(54)-C(55)-C(59)	120.8(12)	C(86)-C(81)-C(80)	119.7(11)
C(67)-C(71)	1.527(16)	C(57)-C(56)-C(51)	117.0(12)	C(81)-C(82)-C(83)	122.5(13)
C(68)-C(69)	1.531(17)	C(57)-C(56)-C(58)	114.8(13)	C(84)-C(83)-C(82)	118.9(13)
C(68)-C(70)	1.531(15)	C(51)-C(56)-C(58)	114.3(12)	C(85)-C(84)-C(83)	120.1(13)
C(71)-C(73)	1.513(15)	C(60)-C(59)-C(61)	112.0(13)	C(84)-C(85)-C(86)	122.0(12)
C(71)-C(72)	1.530(17)	C(60)-C(59)-C(55)	113.0(13)	N(10)-C(86)-C(81)	122.9(10)
C(74)-C(75)	1.402(15)	C(61)-C(59)-C(55)	111.9(13)	N(10)-C(86)-C(85)	119.4(10)
C(74)-C(79)	1.406(15)	C(63)-C(62)-C(67)	123.7(11)	C(81)-C(86)-C(85)	117.6(10)
C(75)-C(76)	1.392(14)	C(63)-C(62)-N(7)	118.6(10)		

Table S12. Photophysical data for Au(I) complexes[a]

	λ_{abs} (nm)[b]	λ_{em} (nm)[c]	τ (μs)[d]	Φ[e]	ΔE_{S1-T1} (meV)[f]	τ_{DP} / τ_{PF} (μs / ns)[g]
[Au(^{Dipp}PZI)(ACD)]	462	598	0.12	0.35	40[g]	N.D.[h] / 20
[Au(^{Dipp}PZI)(Cz)]	475	607	0.11	0.13	55[g]	76 / 15
[Au(^{Dipp}PZI)(DPA)]	549	710	0.017	0.003	49[g]	39 / 40
[Au(^{Dipp}PZI)(DPAC)]	560	717	0.018	0.005	64[g]	34 / 10
[Au(^{Dipp}PZI)(DMAC)]	590	760	0.005	0.001	52[g]	23 / 14
[Au(^{Dipp}PZI)(PXZ)]	619	808	0.004	0.001	110[g]	N.D.[h] /N.D.[h]

[a]10 μM Au(I) complexes dissolved in toluene, 298 K. [b]Absorption peak wavelength. [c]Emission peak wavelength. [d]Weighted average photoluminescence lifetime determined through triexponential fits of the photoluminescence decay traces monitored after pulsed laser photoexcitation at 377 nm. Detection wavelengths: [Au(^{Dipp}PZI)(ACD)], 598 nm; [Au(^{Dipp}PZI)(Cz)], 607 nm; [Au(^{Dipp}PZI)(DPA)], 710 nm; [Au(^{Dipp}PZI)(DPAC)], 717 nm; [Au(^{Dipp}PZI)(DMAC)], 760 nm; [Au(^{Dipp}PZI)(PXZ)], 808 nm. [e]Photoluminescence quantum yields for 10 μM Au(I) complex in deaerated toluene determined relatively using 9,10-diphenylanthracene as a standard (toluene, Φ = 1.00) at 298 K. [f]Energy difference between the singlet and the triplet excited states. [g]Lifetime of delayed photoluminescence (τ_{DP}) and prompt fluorescence (τ_{PF}) which were determined through nonlinear least-squares fits of variable-temperature photoluminescence lifetime data for Zeonex films doped with 5 wt % Au(I) complexes (quartz substrate). [h]Not determined due to low photoluminescence intensities.

Table S13. Geometry parameters of the ground (S_0), S_1, and T_1 states of the Au(I) complexes

	Au–$C_{carbene}$ (Å)[a]			Au–N_{amido} (Å)[b]			$C_{carbene}$–Au–N_{amido} (°)[c]			dihedral angle (°)[d]		
	S_0 (crystal/cal)	S_1	T_1	S_0 (crystal/cal)	S_1	T_1	S_0 (crystal/cal)	S_1	T_1	S_0 (crystal/cal)	S_1	T_1
[Au(^{Dipp}PZI)(ACD)]	1.970/2.006	2.016	2.000	2.040/2.080	2.184	2.135	179/180	180	180	11/0.0	90	2.6
[Au(^{Dipp}PZI)(Cz)]	1.982/2.003	2.042	1.996	2.021/2.038	2.145	2.085	174/180	179	180	0.3/0.0	0	0
[Au(^{Dipp}PZI)(DPA)]	2.000/2.000	2.019	1.996	2.063/2.055	2.166	2.136	177/180	180	180	24/12	77	22
[Au(^{Dipp}PZI)(DPAC)]	1.956/2.001	2.017	1.996	2.036/2.058	2.171	2.143	177/177	179	179	2.8/1.8	90	3.6
[Au(^{Dipp}PZI)(DMAC)]	1.968/2.001	2.019	1.996	2.027/2.056	2.180	2.153	179/177	180	180	3.5/4.3	90	0.0
[Au(^{Dipp}PZI)(PXZ)]	N.D.[e]/1.999	2.018	1.994	N.D.[e]/2.056	2.163	2.137	N.D.[e]/180	180	180	N.D.[e]/0.0	90	3.6

[a]Bond length between Au and the C atom in the carbene ligand. [b]Bond length between Au and the N atom in the amido ligand. [c]Angle between Au–$C_{carbene}$ and Au–N_{amido} bonds. [d]Dihedral angle between $N_{carbene}$–$C_{carbene}$ and N_{amido}–C_{amido} bonds. Here, $N_{carbene}$ and C_{amido} refer to the nitrogen and carbon atoms connected directly to $C_{carbene}$ and N_{amido}, respectively. [e]Single crystal was not obtained.

Table S14. Cartesian coordinate for the optimized T_1 geometry of [Au(^{Dipp}PZI)(ACD)]

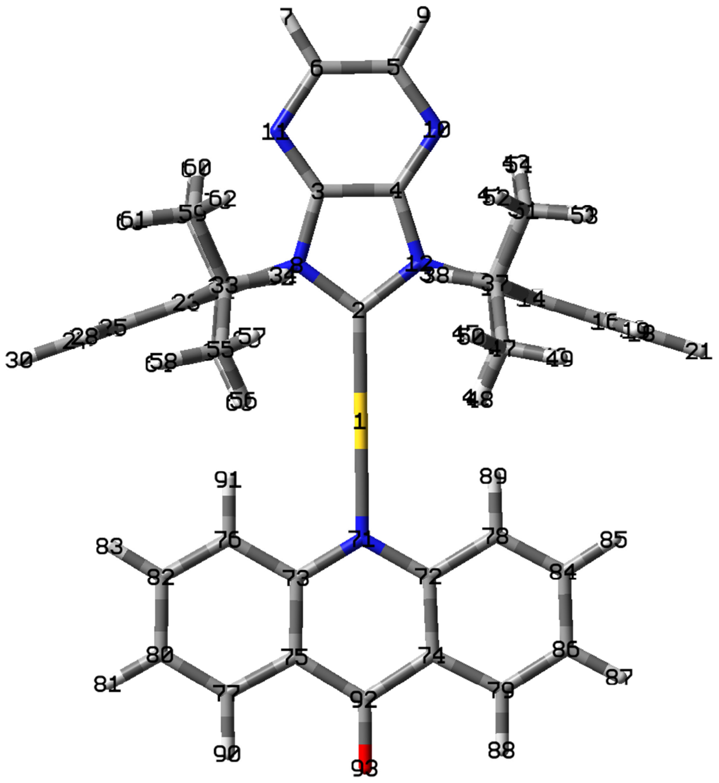

label	atomic number	coordinates (Å)		
		x	*y*	*z*
1	79	-0.70066	-0.000009	0.000003
2	6	1.299282	-0.000011	-0.000005
3	6	3.461177	0.703283	0.011532
4	6	3.461166	-0.703342	-0.01153
5	6	5.687779	-0.691412	-0.01122
6	6	5.687788	0.691328	0.011211
7	1	6.626385	1.236173	0.020055
8	7	2.134058	1.101286	0.018268
9	1	6.626369	-1.23627	-0.02006
10	7	4.554345	-1.456017	-0.02371
11	7	4.554364	1.455946	0.023708
12	7	2.134041	-1.101327	-0.01825
13	6	1.679519	-2.468886	-0.03745
14	6	1.468524	-3.12849	1.18685
15	6	1.484876	-3.100667	-1.27887
16	6	1.028331	-4.454475	1.140301
17	6	1.044101	-4.427233	-1.26801
18	6	0.8146	-5.097788	-0.07282

19	1	0.86353	-4.994848	2.066225
20	1	0.890429	-4.946373	-2.20783
21	1	0.480912	-6.130984	-0.08677
22	6	1.679563	2.468855	0.037467
23	6	1.484909	3.100634	1.278886
24	6	1.46864	3.128481	-1.18684
25	6	1.044184	4.427216	1.268015
26	6	1.028498	4.454483	-1.14029
27	6	0.814747	5.097792	0.072826
28	1	0.890506	4.946354	2.207843
29	1	0.863755	4.994871	-2.06622
30	1	0.4811	6.131001	0.086775
31	6	1.75614	2.466707	-2.5288
32	1	1.916842	1.401848	-2.34797
33	6	1.789326	2.409227	2.602073
34	1	1.950512	1.349068	2.395606
35	6	1.789355	-2.409278	-2.60206
36	1	1.950547	-1.349119	-2.39559
37	6	1.756005	-2.4667	2.528812
38	1	1.916675	-1.401838	2.347961
39	6	3.092873	-2.963416	-3.20826
40	1	2.990576	-4.021773	-3.46728
41	1	3.35281	-2.420491	-4.12217
42	1	3.922536	-2.867683	-2.50483
43	6	0.622164	-2.497387	-3.59855
44	1	-0.29854	-2.093047	-3.17023
45	1	0.852315	-1.923786	-4.50099
46	1	0.423159	-3.526061	-3.9122
47	6	0.577653	-2.578839	3.509676
48	1	-0.33853	-2.165234	3.080335
49	1	0.37602	-3.614504	3.797753
50	1	0.796773	-2.025573	4.42739
51	6	3.053637	-3.031761	3.137467
52	1	3.30265	-2.508148	4.065566
53	1	2.950417	-4.095585	3.372569
54	1	3.891091	-2.920001	2.445665
55	6	0.622089	2.497346	3.598513
56	1	-0.2986	2.092997	3.170162
57	1	0.852201	1.92376	4.500976
58	1	0.423068	3.526025	3.912136
59	6	3.092823	2.963338	3.20834
60	1	3.922519	2.867585	2.504954
61	1	2.99054	4.021697	3.467354
62	1	3.352702	2.420407	4.12226
63	6	0.577769	2.578829	-3.50965

64	1	0.376067	3.6145	-3.79765
65	1	0.796911	2.02563	-4.4274
66	1	-0.33838	2.16514	-3.08032
67	6	3.053752	3.031803	-3.13746
68	1	2.950525	4.095634	-3.37253
69	1	3.891216	2.920026	-2.44567
70	1	3.302759	2.508222	-4.06558
71	7	-2.8353	0.000019	0.00001
72	6	-3.52939	-1.193415	0.034707
73	6	-3.52936	1.193472	-0.0347
74	6	-4.95595	-1.238592	0.033653
75	6	-4.95592	1.238683	-0.03372
76	6	-2.81398	2.412425	-0.07247
77	6	-5.60422	2.474948	-0.06622
78	6	-2.81404	-2.412382	0.07252
79	6	-5.60428	-2.474842	0.066127
80	6	-4.8792	3.661146	-0.10049
81	1	-5.39947	4.612863	-0.1252
82	6	-3.4793	3.626602	-0.10437
83	1	-2.90648	4.546531	-0.13253
84	6	-3.47939	-3.626543	0.104397
85	1	-2.90659	-4.546484	0.132588
86	6	-4.87929	-3.661056	0.100445
87	1	-5.39958	-4.612761	0.125129
88	1	-6.6879	-2.473493	0.06376
89	1	-1.73027	-2.390336	0.076997
90	1	-6.68783	2.473625	-0.0639
91	1	-1.73021	2.390354	-0.07689
92	6	-5.74149	0.000055	-5.1E-05
93	8	-6.97791	0.000071	-8.8E-05

Table S15. Cartesian coordinate for the optimized T_1 geometry of [Au(^{Dipp}PZI)(Cz)]

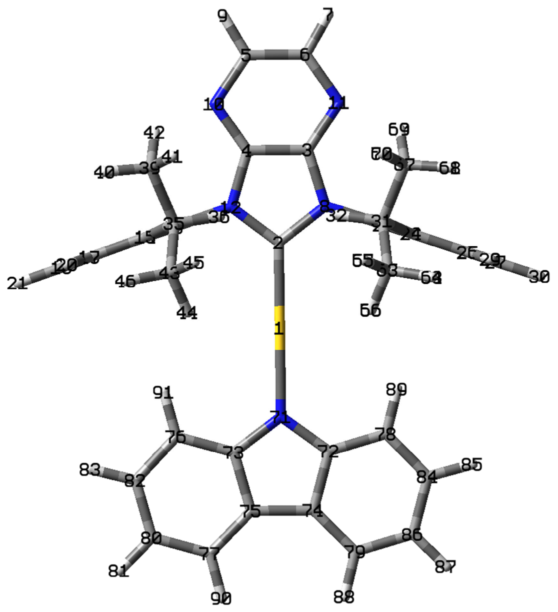

label	atomic number	coordinates (Å)		
		x	*y*	*z*
1	79	0.842949	-0.00046	-0.00006
2	6	-1.153107	0.000191	-0.000062
3	6	-3.320573	0.707173	0.000284
4	6	-3.321149	-0.70502	0.000267
5	6	-5.549667	-0.687912	0.000538
6	6	-5.549105	0.691883	0.000554
7	1	-6.48871	1.235653	0.000676
8	7	-1.998162	1.103628	0.000095
9	1	-6.489715	-1.230915	0.000646
10	7	-4.415054	-1.461528	0.000387
11	7	-4.413861	1.464575	0.000422
12	7	-1.999061	-1.102562	0.000067
13	6	-1.537707	-2.465828	-0.000063
14	6	-1.324422	-3.109474	-1.232352
15	6	-1.324306	-3.109664	1.232109
16	6	-0.865399	-4.429684	-1.204432
17	6	-0.865294	-4.429872	1.203941
18	6	-0.633567	-5.083476	-0.000307
19	1	-0.699153	-4.957341	-2.137542
20	1	-0.698966	-4.957674	2.136955
21	1	-0.283631	-6.111548	-0.000402
22	6	-1.535655	2.466501	0.000008
23	6	-1.32184	3.11001	-1.232263

24	6	-1.321719	3.11012	1.232201
25	6	-0.861688	4.429826	-1.204303
26	6	-0.861577	4.429935	1.204076
27	6	-0.629302	5.083381	-0.000154
28	1	-0.694998	4.957378	-2.137394
29	1	-0.694799	4.95757	2.137105
30	1	-0.278513	6.111162	-0.000217
31	6	-1.631324	2.43534	2.562488
32	1	-1.778397	1.371057	2.367831
33	6	-1.631637	2.435128	-2.562453
34	1	-1.778676	1.370861	-2.367689
35	6	-1.63331	-2.434679	2.562431
36	1	-1.77955	-1.370275	2.367819
37	6	-1.633601	-2.434299	-2.562536
38	1	-1.779835	-1.369927	-2.367746
39	6	-2.949456	-2.982899	3.146352
40	1	-2.861448	-4.046686	3.388898
41	1	-3.212941	-2.451393	4.066148
42	1	-3.769827	-2.865649	2.435379
43	6	-0.47842	-2.548923	3.570043
44	1	0.451239	-2.151054	3.154708
45	1	-0.710733	-1.98122	4.47579
46	1	-0.296002	-3.583186	3.876166
47	6	-0.478845	-2.548378	-3.570314
48	1	0.450857	-2.150524	-3.155059
49	1	-0.296432	-3.582597	-3.87659
50	1	-0.711308	-1.980575	-4.475961
51	6	-2.949816	-2.982456	-3.146373
52	1	-3.213419	-2.450822	-4.066062
53	1	-2.86182	-4.046206	-3.389081
54	1	-3.770104	-2.865322	-2.435286
55	6	-0.476874	2.548346	-3.570312
56	1	0.452542	2.149718	-3.15516
57	1	-0.70987	1.980788	-4.475976
58	1	-0.293656	3.582436	-3.87654
59	6	-2.94747	2.984331	-3.146176
60	1	-3.767803	2.867814	-2.435038
61	1	-2.858658	4.04802	-3.388859
62	1	-3.211544	2.452932	-4.065863
63	6	-0.476398	2.548627	3.570159
64	1	-0.293131	3.582739	3.876287
65	1	-0.70924	1.981126	4.475897
66	1	0.452951	2.149976	3.154879
67	6	-2.947063	2.984589	3.146366
68	1	-2.858214	4.048293	3.388973

69	1	-3.767494	2.86803	2.435348
70	1	-3.211013	2.453246	4.066123
71	7	2.92775	-0.000802	-0.000124
72	6	3.746948	1.110711	-0.000088
73	6	3.746711	-1.112491	-0.000034
74	6	5.119193	0.729669	0.000037
75	6	5.119038	-0.731737	0.000071
76	6	3.359647	-2.457156	-0.000045
77	6	6.102207	-1.703308	0.000175
78	6	3.360157	2.455456	-0.000166
79	6	6.102562	1.701038	0.000094
80	6	5.713776	-3.055101	0.00017
81	1	6.476731	-3.826174	0.000253
82	6	4.365633	-3.422825	0.000061
83	1	4.096111	-4.473004	0.000058
84	6	4.366344	3.420916	-0.000108
85	1	4.097036	4.47115	-0.000165
86	6	5.71441	3.052912	0.000022
87	1	6.477525	3.823826	0.000066
88	1	7.155332	1.43811	0.000194
89	1	2.311282	2.73054	-0.000269
90	1	7.155031	-1.440598	0.000262
91	1	2.310716	-2.73202	-0.000132

Table S16. Cartesian coordinate for the optimized T1 geometry of [Au(^{Dipp}PZI)(DPA)]

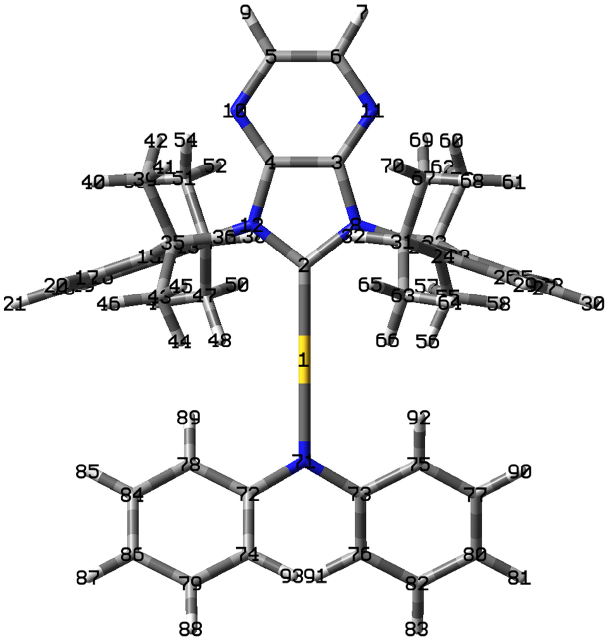

label	atomic number	coordinates (Å)		
		x	*y*	*z*
1	79	0.916099	0.000011	0.000009
2	6	-1.079707	-0.00001	0.000016
3	6	-3.248448	0.698754	0.095516
4	6	-3.248429	-0.698811	-0.09566
5	6	-5.478126	-0.682364	-0.094696
6	6	-5.478143	0.682266	0.094427
7	1	-6.417016	1.222091	0.169375
8	7	-1.923957	1.093214	0.147783
9	1	-6.416985	-1.222207	-0.169695
10	7	-4.341044	-1.447371	-0.20014
11	7	-4.341082	1.447294	0.199935
12	7	-1.923927	-1.09325	-0.147827
13	6	-1.466975	-2.445012	-0.329759
14	6	-1.266209	-2.92397	-1.63725
15	6	-1.258275	-3.248187	0.806016
16	6	-0.824263	-4.242229	-1.785371
17	6	-0.818043	-4.559515	0.602298
18	6	-0.599541	-5.052221	-0.678606
19	1	-0.667469	-4.64521	-2.780218
20	1	-0.656701	-5.207808	1.456949
21	1	-0.266458	-6.076821	-0.815869
22	6	-1.467047	2.444985	0.329763

23	6	-1.258305	3.248186	-0.805988
24	6	-1.266372	2.923926	1.637272
25	6	-0.818124	4.559523	-0.602221
26	6	-0.824484	4.2422	1.785442
27	6	-0.599721	5.052217	0.678705
28	1	-0.656747	5.207835	-1.456852
29	1	-0.667764	4.645168	2.780306
30	1	-0.266679	6.076826	0.816005
31	6	-1.575587	2.076148	2.864575
32	1	-1.693419	1.042813	2.532278
33	6	-1.556784	2.749893	-2.214224
34	1	-1.673091	1.665556	-2.163472
35	6	-1.556845	-2.749877	2.214227
36	1	-1.673163	-1.665542	2.163451
37	6	-1.5754	-2.076227	-2.864583
38	1	-1.693255	-1.042884	-2.532314
39	6	-2.890767	-3.335415	2.716586
40	1	-2.831255	-4.423873	2.817497
41	1	-3.146029	-2.922613	3.697604
42	1	-3.703718	-3.104472	2.025287
43	6	-0.413065	-3.029586	3.201957
44	1	0.530782	-2.60671	2.847989
45	1	-0.637993	-2.581148	4.174091
46	1	-0.262285	-4.100204	3.368903
47	6	-0.438405	-2.084088	-3.898612
48	1	0.506881	-1.762579	-3.453151
49	1	-0.285013	-3.073464	-4.33932
50	1	-0.670888	-1.399053	-4.719205
51	6	-2.912136	-2.51515	-3.493326
52	1	-3.176097	-1.862054	-4.331132
53	1	-2.851743	-3.539519	-3.874787
54	1	-3.719657	-2.474946	-2.75959
55	6	-0.412949	3.029631	-3.201883
56	1	0.530884	2.606768	-2.847863
57	1	-0.63781	2.581197	-4.174034
58	1	-0.262178	4.100253	-3.368811
59	6	-2.890683	3.335424	-2.716654
60	1	-3.70367	3.104466	-2.025403
61	1	-2.831174	4.423883	-2.817549
62	1	-3.145885	2.922629	-3.697691
63	6	-0.43861	2.084002	3.898625
64	1	-0.285234	3.073373	4.339351
65	1	-0.6711	1.398951	4.719204
66	1	0.506685	1.762509	3.453173
67	6	-2.912342	2.515032	3.493302

68	1	-2.85198	3.539393	3.874791
69	1	-3.719848	2.47483	2.75955
70	1	-3.176308	1.86191	4.331086
71	7	3.052445	0.000033	0.000018
72	6	3.735489	-1.172815	0.288128
73	6	3.735478	1.172886	-0.288089
74	6	5.002563	-1.183227	0.921039
75	6	3.111581	2.412675	-0.013773
76	6	5.00255	1.183313	-0.921004
77	6	3.751879	3.604264	-0.310925
78	6	3.111604	-2.41261	0.013817
79	6	5.621252	-2.383665	1.233476
80	6	5.009398	3.599638	-0.919856
81	1	5.499558	4.535317	-1.166303
82	6	5.621225	2.383757	-1.233441
83	1	6.580144	2.37318	-1.740744
84	6	3.751916	-3.604192	0.310968
85	1	3.261453	-4.542415	0.076471
86	6	5.009438	-3.599552	0.919894
87	1	5.499608	-4.535226	1.16634
88	1	6.580173	-2.373076	1.740777
89	1	2.13152	-2.418551	-0.448955
90	1	3.261408	4.542482	-0.076424
91	1	5.462803	0.247614	-1.211956
92	1	2.131499	2.418602	0.449004
93	1	5.462807	-0.247523	1.211989

Table S17. Cartesian coordinate for the optimized T_1 geometry of [Au(^{Dipp}PZI)(DPAC)]

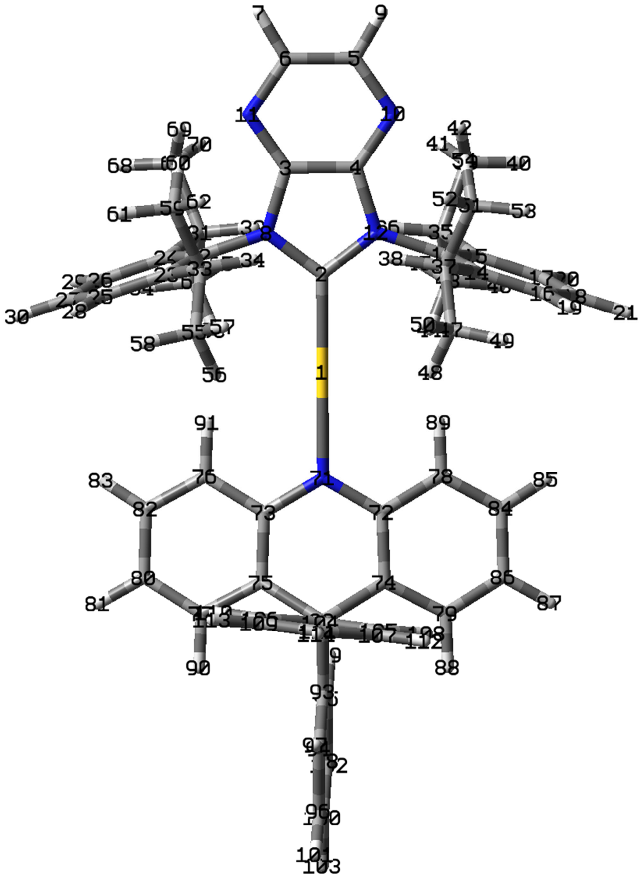

label	atomic number	coordinates (Å)		
		x	*y*	*z*
1	79	0.37915	-0.007575	-0.259237
2	6	2.360058	0.002548	-0.019087
3	6	4.502911	0.719592	0.29453
4	6	4.511845	-0.690588	0.286978
5	6	6.719301	-0.661873	0.598595
6	6	6.710541	0.715764	0.606024
7	1	7.636762	1.265977	0.740007
8	7	3.189299	1.109788	0.107521
9	1	7.652467	-1.201661	0.726685
10	7	5.598519	-1.440149	0.435163
11	7	5.579905	1.48137	0.450869
12	7	3.203273	-1.095522	0.095947
13	6	2.761333	-2.462146	0.019185
14	6	2.398583	-3.125362	1.205335
15	6	2.728043	-3.094877	-1.23714
16	6	1.971586	-4.452682	1.102722
17	6	2.292564	-4.422864	-1.284148
18	6	1.914505	-5.095142	-0.127859

19	1	1.691451	-4.994701	1.999632
20	1	2.264209	-4.942994	-2.235627
21	1	1.588282	-6.129533	-0.184392
22	6	2.729607	2.47117	0.043557
23	6	2.350833	3.116037	1.234768
24	6	2.694138	3.11716	-1.20583
25	6	1.906294	4.438402	1.144386
26	6	2.240626	4.439552	-1.240759
27	6	1.847095	5.093739	-0.079263
28	1	1.613174	4.966171	2.045611
29	1	2.210761	4.969759	-2.186659
30	1	1.506417	6.123925	-0.126151
31	6	3.194114	2.444624	-2.478274
32	1	3.29825	1.378008	-2.270448
33	6	2.471508	2.440766	2.594927
34	1	2.647455	1.376754	2.42438
35	6	3.213292	-2.402157	-2.504558
36	1	3.297471	-1.335534	-2.287693
37	6	2.518608	-2.464002	2.572412
38	1	2.682839	-1.396365	2.413026
39	6	4.618245	-2.909412	-2.8838
40	1	4.596921	-3.972843	-3.142897
41	1	5.003405	-2.362271	-3.750032
42	1	5.317951	-2.777384	-2.05612
43	6	2.231449	-2.541978	-3.678607
44	1	1.237728	-2.171065	-3.413877
45	1	2.586137	-1.963101	-4.53636
46	1	2.127836	-3.578845	-4.011415
47	6	1.238555	-2.596675	3.413222
48	1	0.371276	-2.194575	2.883215
49	1	1.021499	-3.636106	3.676548
50	1	1.345152	-2.042337	4.350379
51	6	3.747197	-3.011051	3.324566
52	1	3.878092	-2.488479	4.277354
53	1	3.635613	-4.078148	3.541934
54	1	4.655997	-2.879334	2.73387
55	6	1.186251	2.550658	3.431125
56	1	0.325562	2.146319	2.892082
57	1	1.294056	1.986107	4.362022
58	1	0.957682	3.584666	3.705938
59	6	3.690844	2.99199	3.359007
60	1	4.603814	2.876377	2.771334
61	1	3.567203	4.055394	3.587643
62	1	3.822719	2.460165	4.306527
63	6	2.210382	2.57562	-3.651709

64	1	2.084373	3.613503	-3.973505
65	1	2.577552	2.01347	-4.515303
66	1	1.225021	2.180079	-3.391216
67	6	4.589628	2.981289	-2.851481
68	1	4.549125	4.046448	-3.101047
69	1	5.290522	2.854534	-2.023978
70	1	4.985976	2.449173	-3.722006
71	7	-1.751749	-0.016948	-0.489354
72	6	-2.444548	-1.20654	-0.56042
73	6	-2.448507	1.164796	-0.629518
74	6	-3.863487	-1.260916	-0.496359
75	6	-3.867036	1.218265	-0.565849
76	6	-1.721643	2.350363	-0.897269
77	6	-4.499517	2.426376	-0.840681
78	6	-1.712941	-2.402607	-0.760167
79	6	-4.490428	-2.484191	-0.711497
80	6	-3.771434	3.580321	-1.137905
81	1	-4.295712	4.506153	-1.349753
82	6	-2.376115	3.541716	-1.148944
83	1	-1.799402	4.434581	-1.3626
84	6	-2.362162	-3.608102	-0.948994
85	1	-1.781876	-4.509112	-1.112769
86	6	-3.757404	-3.649969	-0.943076
87	1	-4.278146	-4.58694	-1.110031
88	1	-5.572452	-2.533833	-0.691348
89	1	-0.630608	-2.353516	-0.791225
90	1	-5.58126	2.474646	-0.809499
91	1	-0.639088	2.304621	-0.928262
92	6	-4.639757	-0.009183	-0.076824
93	6	-6.064263	-0.019422	-0.676127
94	6	-7.219199	0.063481	0.102987
95	6	-6.208915	-0.096168	-2.069817
96	6	-8.484165	0.06508	-0.490949
97	1	-7.143066	0.127947	1.181089
98	6	-7.465708	-0.095292	-2.662356
99	1	-5.325184	-0.157007	-2.696636
100	6	-8.613365	-0.015289	-1.872129
101	1	-9.367286	0.129837	0.136679
102	1	-7.550546	-0.156295	-3.742707
103	1	-9.596002	-0.014735	-2.33261
104	6	-4.678721	0.02424	1.49273
105	6	-4.840696	-1.155403	2.231215
106	6	-4.602233	1.229208	2.201116
107	6	-4.92938	-1.131202	3.620688
108	1	-4.895383	-2.111121	1.726402

109	6	-4.689341	1.255113	3.592281
110	1	-4.471412	2.16516	1.674458
111	6	-4.85308	0.075531	4.310694
112	1	-5.051611	-2.063518	4.162759
113	1	-4.621159	2.205776	4.111449
114	1	-4.913459	0.094623	5.393923

Table S18. Cartesian coordinate for the optimized T_1 geometry of [Au(^{Dipp}PZI)(DMAC)]

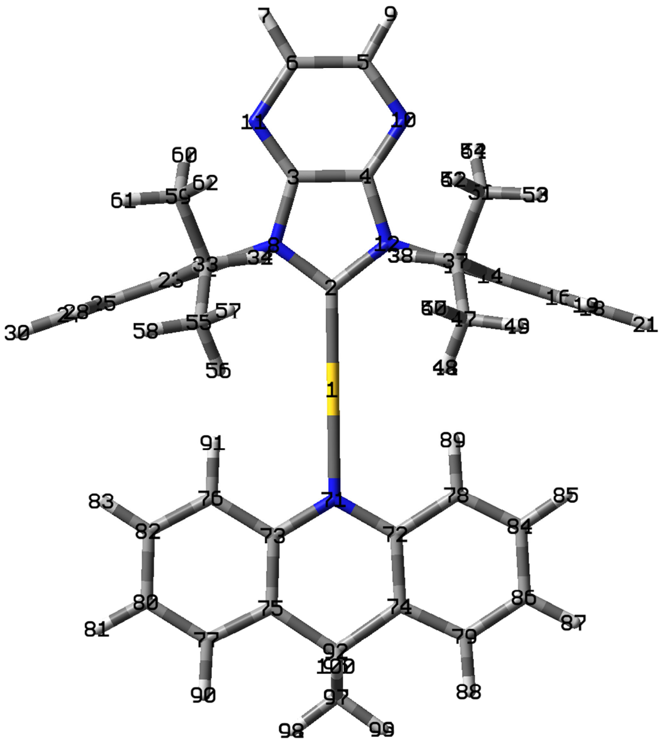

label	atomic number	coordinates (Å)		
		x	*y*	*z*
1	79	0.559232	-0.000009	0.000108
2	6	-1.436777	-0.000006	-0.000023
3	6	-3.606997	-0.704999	-0.000202
4	6	-3.606993	0.704995	-0.00024
5	6	-5.836976	0.688861	-0.00043
6	6	-5.836981	-0.688849	-0.00039
7	1	-6.775545	-1.234355	-0.000451
8	7	-2.282246	-1.103067	-0.000073
9	1	-6.775538	1.234373	-0.000525
10	7	-4.699772	1.459884	-0.000354
11	7	-4.699781	-1.459879	-0.00027
12	7	-2.282242	1.103057	-0.000134
13	6	-1.828723	2.46826	-0.0001
14	6	-1.624363	3.11581	1.232055
15	6	-1.624001	3.115755	-1.232227
16	6	-1.184817	4.442695	1.204199
17	6	-1.184464	4.442639	-1.204302
18	6	-0.963522	5.100177	-0.000033
19	1	-1.026957	4.972866	2.137299
20	1	-1.026326	4.972767	-2.137379

21	1	-0.63195	6.134417	-0.000009
22	6	-1.828714	-2.468266	0.000035
23	6	-1.62417	-3.115694	1.232228
24	6	-1.624147	-3.115872	-1.232057
25	6	-1.184587	-4.442566	1.204436
26	6	-1.184575	-4.442745	-1.204066
27	6	-0.963445	-5.100159	0.000233
28	1	-1.026571	-4.972646	2.137561
29	1	-1.026553	-4.972956	-2.137115
30	1	-0.631844	-6.13439	0.000313
31	6	-1.926294	-2.437018	-2.562007
32	1	-2.044763	-1.36904	-2.368292
33	6	-1.926392	-2.436646	2.562063
34	1	-2.044575	-1.368659	2.36822
35	6	-1.925964	2.436763	-2.56215
36	1	-2.044273	1.36878	-2.368365
37	6	-1.926708	2.436892	2.561929
38	1	-2.045239	1.368936	2.368134
39	6	-3.259326	2.954895	-3.134647
40	1	-3.197021	4.020777	-3.376271
41	1	-3.517809	2.418707	-4.053189
42	1	-4.071214	2.818579	-2.417475
43	6	-0.783194	2.581699	-3.579537
44	1	0.160958	2.210606	-3.172417
45	1	-1.007972	2.006404	-4.48241
46	1	-0.632625	3.620143	-3.889055
47	6	-0.784056	2.581573	3.579487
48	1	0.160045	2.210155	3.172544
49	1	-0.633221	3.620002	3.888926
50	1	-1.009148	2.00643	4.48238
51	6	-3.260043	2.955322	3.134212
52	1	-3.518786	2.419189	4.052712
53	1	-3.197554	4.021191	3.375848
54	1	-4.071843	2.819172	2.416909
55	6	-0.783884	-2.581636	3.579736
56	1	0.160403	-2.210644	3.172836
57	1	-1.008837	-2.00628	4.482526
58	1	-0.633487	-3.620077	3.889345
59	6	-3.259925	-2.954672	3.134259
60	1	-4.071637	-2.818334	2.416893
61	1	-3.197739	-4.020551	3.375931
62	1	-3.518588	-2.418437	4.052723
63	6	-0.783555	-2.58185	-3.579446
64	1	-0.6328	-3.620301	-3.888847
65	1	-1.0085	-2.006706	-4.482374

66	1	0.160548	-2.210523	-3.172423
67	6	-3.259608	-2.955391	-3.134399
68	1	-3.197154	-4.021277	-3.375972
69	1	-4.071473	-2.819154	-2.417189
70	1	-3.51822	-2.419292	-4.052956
71	7	2.71194	0.000001	0.000221
72	6	3.403298	1.193191	0.000209
73	6	3.403311	-1.193182	0.000186
74	6	4.82206	1.257416	0.000039
75	6	4.822074	-1.257392	0.000006
76	6	2.655085	-2.398424	0.00032
77	6	5.423025	-2.516131	-0.000059
78	6	2.65506	2.398426	0.000365
79	6	5.422997	2.51616	0.000008
80	6	4.676361	-3.69162	0.000063
81	1	5.182123	-4.651364	0.000007
82	6	3.280831	-3.62815	0.000261
83	1	2.684106	-4.533234	0.000363
84	6	3.280792	3.628158	0.000339
85	1	2.684059	4.533237	0.000458
86	6	4.676322	3.691642	0.000152
87	1	5.182076	4.651391	0.000123
88	1	6.50466	2.591083	-0.000134
89	1	1.572972	2.33978	0.000516
90	1	6.504688	-2.591042	-0.000211
91	1	1.572997	-2.33979	0.000479
92	6	5.681577	0.000016	-0.000153
93	6	6.579311	0.000039	-1.268799
94	1	7.223333	-0.881068	-1.292665
95	1	5.971842	0.000052	-2.176193
96	1	7.223329	0.88115	-1.292639
97	6	6.579843	0.000002	1.268125
98	1	7.223882	-0.8811	1.291697
99	1	7.223872	0.881109	1.291727
100	1	5.972742	-0.000016	2.175764

Table S19. Cartesian coordinate for the optimized T1 geometry of [Au(^{Dipp}PZI)(PXZ)]

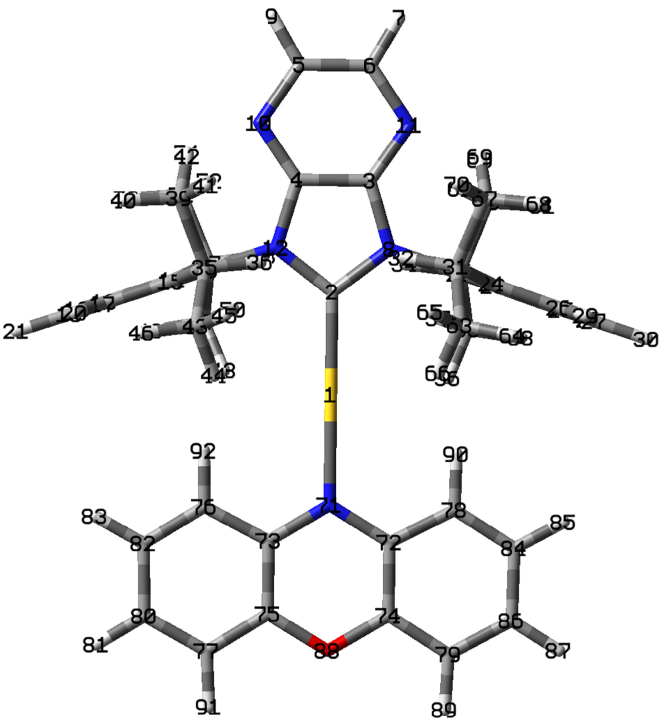

label	atomic number	coordinates (Å)		
		x	*y*	*z*
1	79	0.795981	0.000152	0.000039
2	6	-1.198445	-0.000096	-0.000038
3	6	-3.367907	0.704614	0.014145
4	6	-3.367686	-0.705486	-0.014367
5	6	-5.597646	-0.689496	-0.013993
6	6	-5.597855	0.687952	0.013645
7	1	-6.536351	1.2334	0.024465
8	7	-2.043098	1.10287	0.022228
9	1	-6.535975	-1.23523	-0.024867
10	7	-4.460207	-1.459782	-0.029442
11	7	-4.460653	1.458581	0.029157
12	7	-2.042755	-1.103332	-0.022359
13	6	-1.584294	-2.466612	-0.050479
14	6	-1.396237	-3.094548	-1.294998
15	6	-1.354405	-3.13048	1.168705
16	6	-0.944972	-4.417945	-1.294018
17	6	-0.903713	-4.452712	1.113859
18	6	-0.696885	-5.0905	-0.10373
19	1	-0.798458	-4.933156	-2.237337
20	1	-0.726349	-4.995428	2.03619

21	1	-0.355925	-6.121453	-0.124512
22	6	-1.585107	2.466306	0.050424
23	6	-1.355259	3.130263	-1.168721
24	6	-1.397502	3.094307	1.294969
25	6	-0.905048	4.452652	-1.113795
26	6	-0.946732	4.417877	1.294069
27	6	-0.698667	5.090519	0.103832
28	1	-0.727729	4.995434	-2.036097
29	1	-0.800593	4.933138	2.23742
30	1	-0.358083	6.121595	0.124672
31	6	-1.726502	2.40003	2.610484
32	1	-1.879261	1.340301	2.396849
33	6	-1.644048	2.473069	-2.51244
34	1	-1.758324	1.401399	-2.337647
35	6	-1.643606	-2.473363	2.512374
36	1	-1.758151	-1.401727	2.337548
37	6	-1.725342	-2.40037	-2.610538
38	1	-1.877926	-1.340599	-2.396977
39	6	-2.975416	-2.995831	3.084802
40	1	-2.915567	-4.065807	3.308221
41	1	-3.226026	-2.473988	4.013726
42	1	-3.791757	-2.845029	2.375563
43	6	-0.495357	-2.639907	3.520077
44	1	0.448385	-2.26781	3.112636
45	1	-0.711024	-2.076468	4.43251
46	1	-0.348714	-3.68344	3.813977
47	6	-0.580771	-2.490125	-3.632331
48	1	0.348946	-2.08688	-3.222504
49	1	-0.390562	-3.518858	-3.952081
50	1	-0.829575	-1.914712	-4.528858
51	6	-3.043927	-2.948885	-3.188626
52	1	-3.321136	-2.404437	-4.09672
53	1	-2.951498	-4.007971	-3.449513
54	1	-3.85676	-2.848162	-2.46665
55	6	-0.49567	2.639942	-3.519944
56	1	0.448102	2.268109	-3.112332
57	1	-0.711015	2.076442	-4.432414
58	1	-0.349268	3.683518	-3.813812
59	6	-2.97591	2.995165	-3.08508
60	1	-3.792326	2.84411	-2.375979
61	1	-2.916339	4.065165	-3.308457
62	1	-3.226224	2.473278	-4.014059
63	6	-0.581694	2.489549	3.632042
64	1	-0.391153	3.518259	3.951669
65	1	-0.830437	1.914243	4.528656

66	1	0.347824	2.086051	3.222015
67	6	-3.044923	2.948607	3.188862
68	1	-2.952401	4.00766	3.449853
69	1	-3.857883	2.847997	2.467011
70	1	-3.322022	2.404086	4.096946
71	7	2.932485	0.000314	0.000055
72	6	3.646089	1.174848	-0.048965
73	6	3.646258	-1.174125	0.049078
74	6	5.062489	1.172361	-0.047985
75	6	5.062657	-1.171434	0.048105
76	6	2.997312	-2.429056	0.102462
77	6	5.798538	-2.349508	0.096048
78	6	2.996957	2.429685	-0.102348
79	6	5.798195	2.350547	-0.095931
80	6	5.130839	-3.565627	0.146859
81	1	5.698598	-4.488513	0.184366
82	6	3.728426	-3.599975	0.150171
83	1	3.207552	-4.54964	0.190415
84	6	3.727897	3.60071	-0.150057
85	1	3.206888	4.550301	-0.1903
86	6	5.130318	3.566566	-0.146746
87	1	5.697941	4.489535	-0.184251
88	1	5.760419	0.000516	0.000072
89	1	6.880132	2.289061	-0.092619
90	1	1.913325	2.452908	-0.105856
91	1	6.880465	-2.28786	0.092741
92	6	1.913685	-2.452455	0.105975

Table S20. Summary of the TD-CAM-B3LYP calculation results for the T_1 geometry of [Au(^{Dipp}PZI)(ACD)]

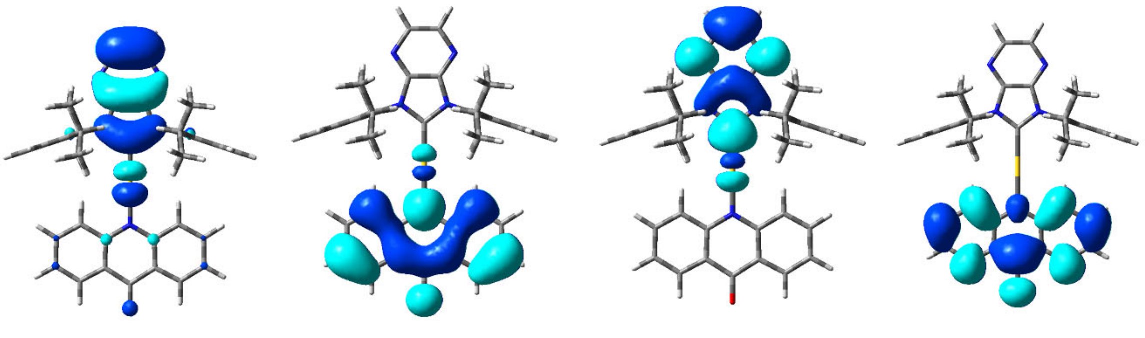

state	energy (eV)	participating molecular orbitals (expansion coefficient)	transition character
T_1	2.49	HOMO → LUMO+1 (0.94)	$\pi-\pi^*_{ACD}$
T_2	3.08	HOMO-9 → LUMO (0.56)<br>HOMO → LUMO (0.34)	$\pi-\pi^*_{DippPZI}$ + LLCT
T_3	3.19	HOMO → LUMO+1 (0.42)	$\pi-\pi^*_{ACD}$
T_4	3.33	HOMO-9 → LUMO (0.26)<br>HOMO → LUMO (0.21)	$\pi-\pi^*_{DippPZI}$ + LLCT
S_1	3.38	HOMO → LUMO (0.91)	LLCT

Table S21. Summary of the TD-CAM-B3LYP calculation results for the T_1 geometry of [Au(^{Dipp}PZI)(Cz)]

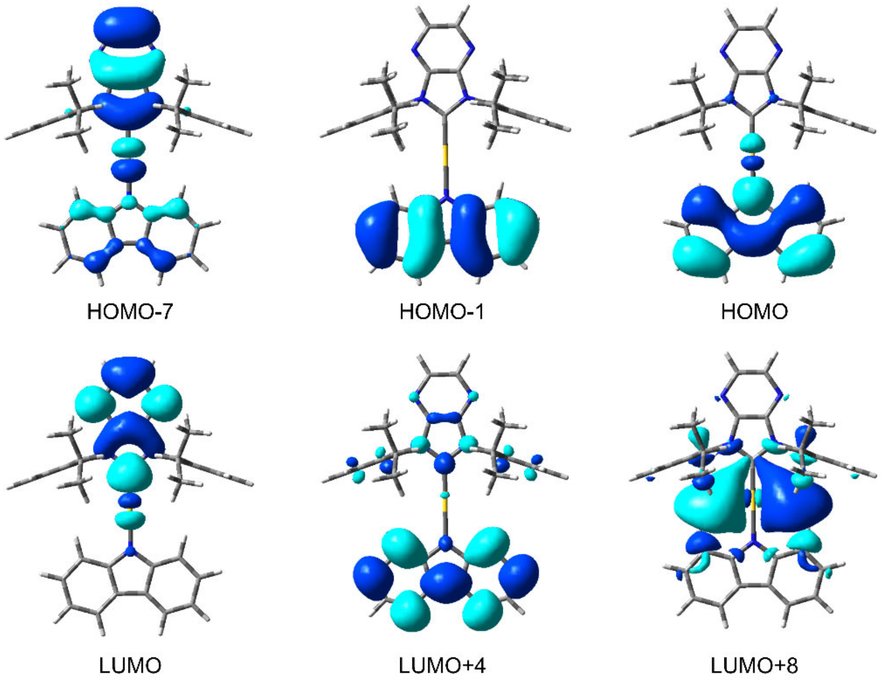

state	energy (eV)	participating molecular orbitals (expansion coefficient)	transition character
T_1	2.94	HOMO → LUMO (0.47)<br>HOMO-7 → LUMO (0.34)	$\pi-\pi^{*}{}_{DippPZI}$
T_2	3.10	HOMO → LUMO+4 (0.59)	$\pi-\pi^{*}{}_{Cz}$
T_3	3.13	HOMO-1 → LUMO+4 (0.40)<br>HOMO → LUMO+8 (0.31)	$\pi-\pi^{*}{}_{Cz}$ + LMCT
S_1	3.23	HOMO → LUMO (0.91)	LLCT

Table S22. Summary of the TD-CAM-B3LYP calculation results for the T_1 geometry of [Au(^{Dipp}PZI)(DPA)]

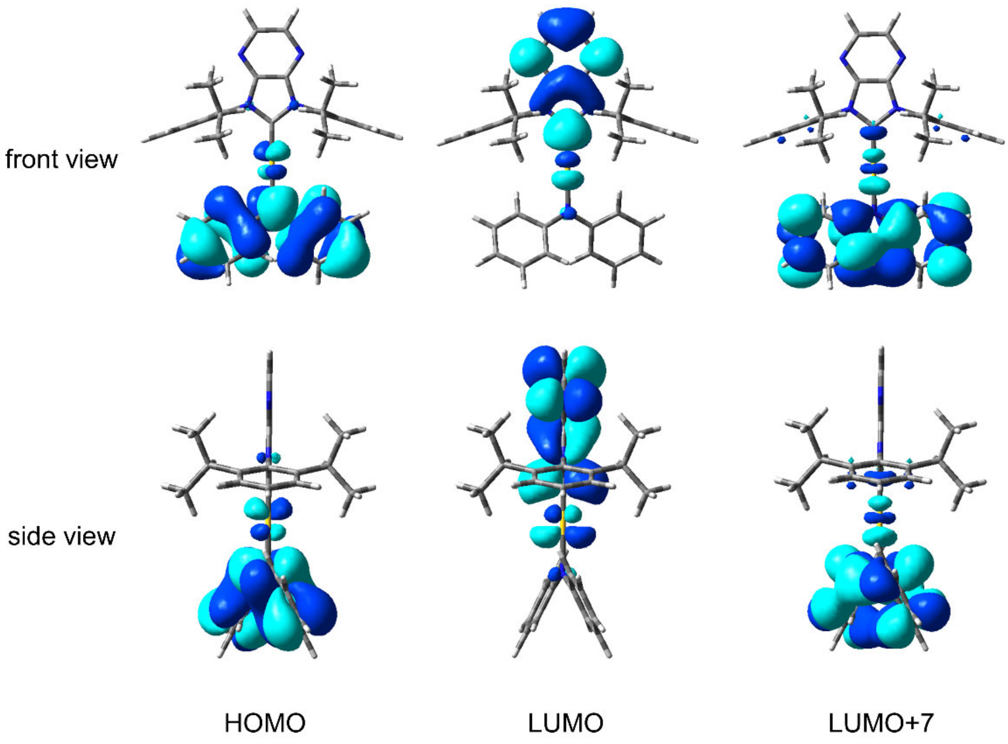

state	energy (eV)	participating molecular orbitals (expansion coefficient)	transition character
T_1	2.64	HOMO → LUMO (0.83)	LLCT
S_1	2.78	HOMO → LUMO (0.92)	LLCT
T_2	2.84	HOMO → LUMO+7 (0.62)	π–π^*_{DPA}

Table S23. Summary of the TD-CAM-B3LYP calculation results for the T_1 geometry of [Au(^{Dipp}PZI)(DPAC)]

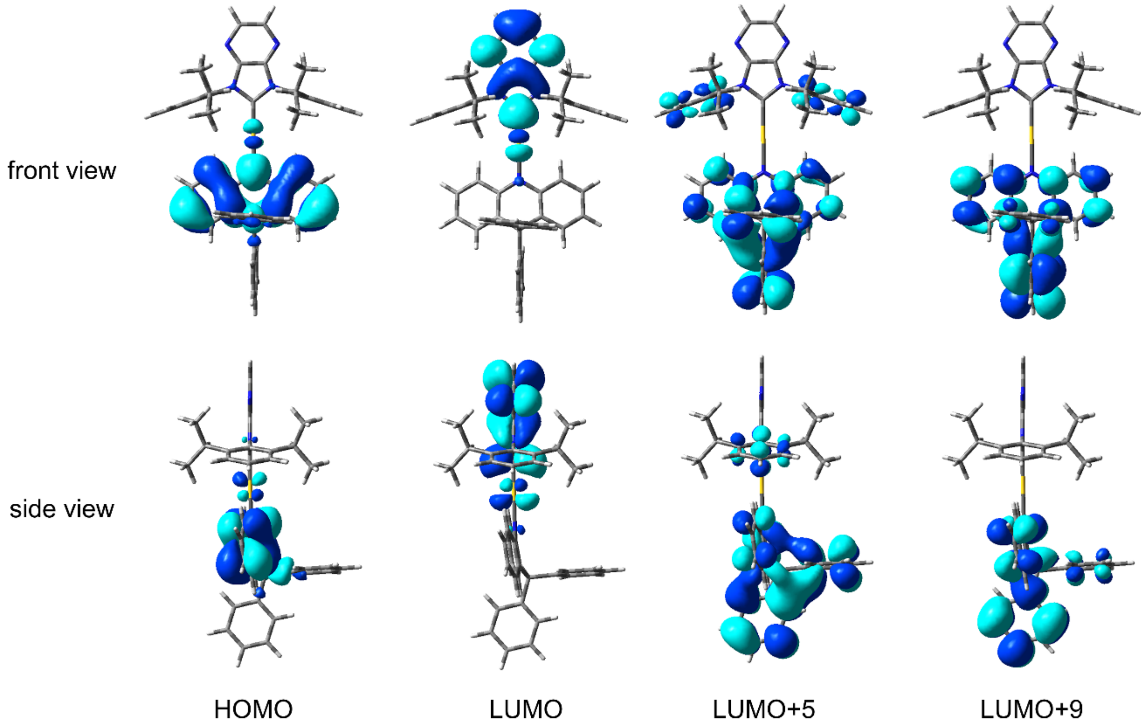

state	energy (eV)	participating molecular orbitals (expansion coefficient)	transition character
T_1	2.65	HOMO → LUMO (0.80)	LLCT
S_1	2.79	HOMO → LUMO (0.91)	LLCT
T_2	2.93	HOMO → LUMO+5 (0.22)<br>HOMO → LUMO+9 (0.22)	$\pi-\pi^*_{DPAC}$

Table S24. Summary of the TD-CAM-B3LYP calculation results for the T_1 geometry of $[Au(^{Dipp}PZI)(DMAC)]$

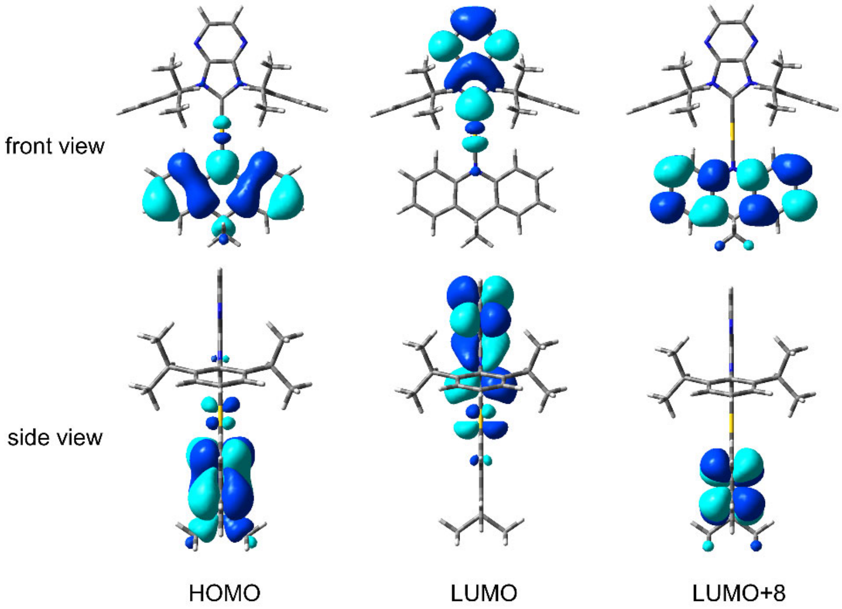

state	energy (eV)	participating molecular orbitals (expansion coefficient)	transition character
T_1	2.50	HOMO → LUMO (0.84)	LLCT
S_1	2.63	HOMO → LUMO (0.91)	LLCT
T_2	2.87	HOMO → LUMO+8 (0.78)	π–π^*_{DMAC}

Table S25. Summary of the TD-CAM-B3LYP calculation results for the T_1 geometry of $[Au(^{Dipp}PZI)(PXZ)]$

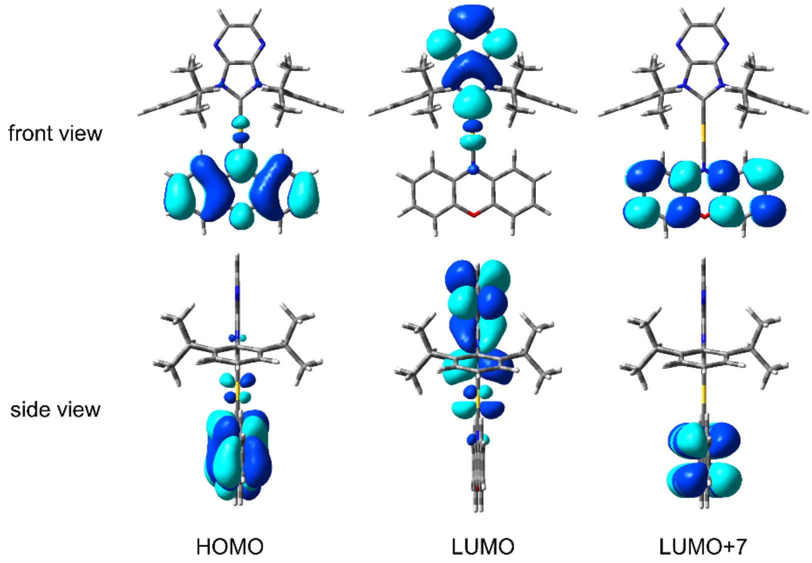

state	energy (eV)	participating molecular orbitals (expansion coefficient)	transition character
T_1	2.27	HOMO → LUMO (0.87)	LLCT
S_1	2.40	HOMO → LUMO (0.92)	LLCT
T_2	2.41	HOMO → LUMO+7 (0.78)	$\pi-\pi^*_{PXZ}$

Table S26. $S_r(r)$ values calculated for the fragments in Au(I) complexes[a]

	[Au(^{Dipp}PZI)(ACD)]	[Au(^{Dipp}PZI)(Cz)]	[Au(^{Dipp}PZI)(DPA)]	[Au(^{Dipp}PZI)(DPAC)]	[Au(^{Dipp}PZI)(DMAC)]	[Au(^{Dipp}PZI)(PXZ)]
$S_{r,carbene}(r)$	0.10	0.10	0.09	0.09	0.09	0.10
$S_{r,Au}(r)$	0.06	0.06	0.05	0.05	0.05	0.05
$S_{r,amido}(r)$	0.16	0.14	0.12	0.15	0.15	0.16

[a]Fragmental $S_r(r)$ values were calcuated for the carbene ligand ($S_{r,carbene}(r)$), Au(I) ($S_{r,Au}(r)$), and the amido ligand ($S_{r,amido}(r)$).

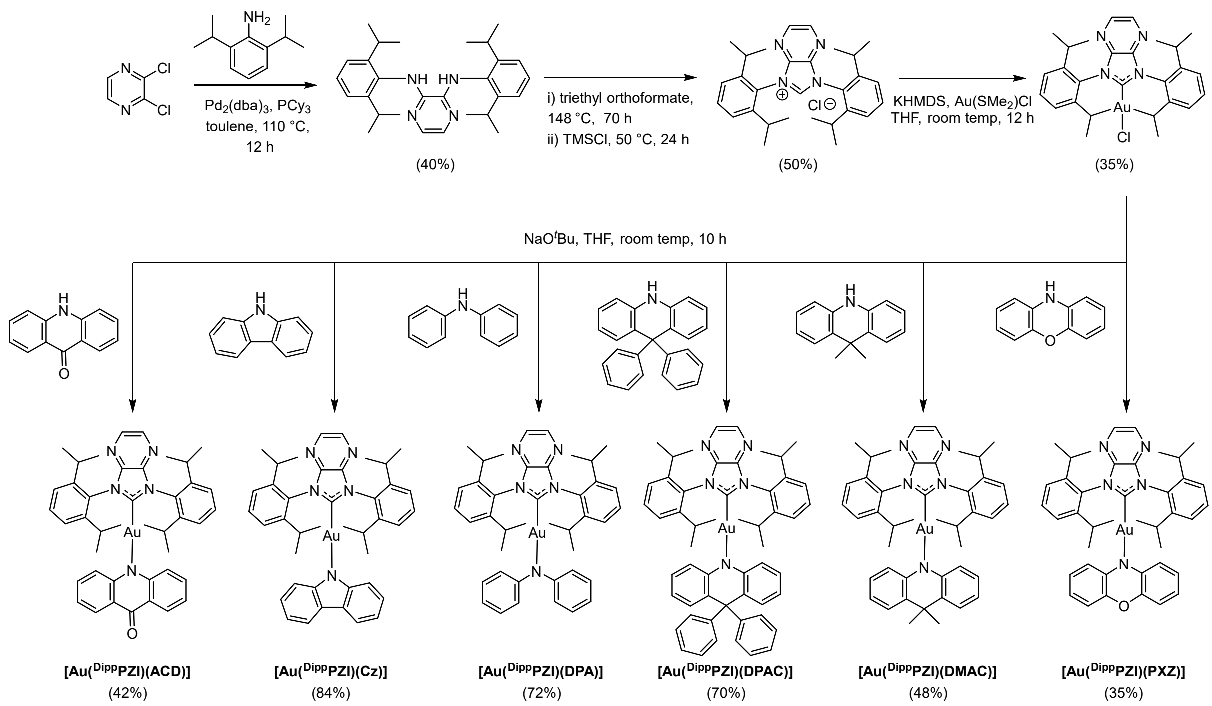

Scheme S1. Syntheses of the Au(I) complexes.

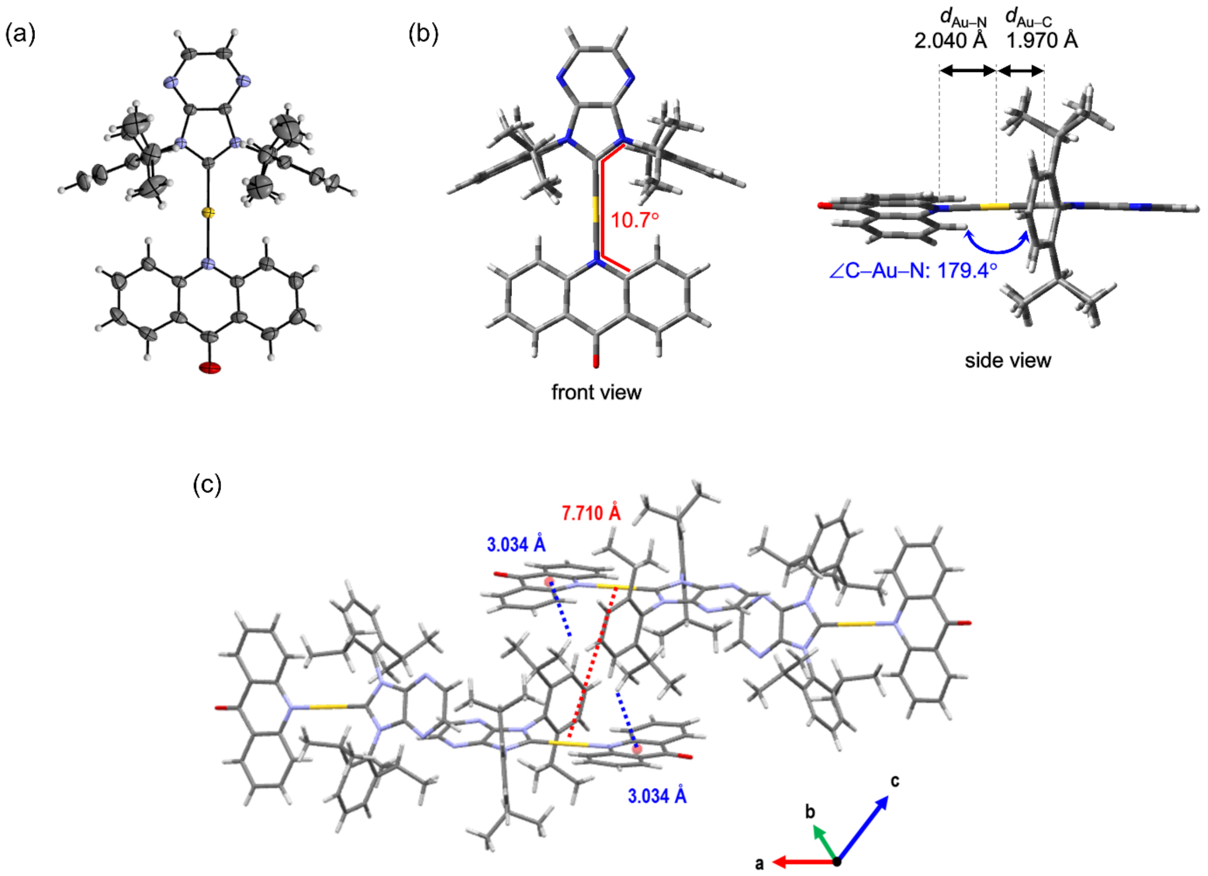

Figure S1. Single crystal X-ray structure of [Au(^{Dipp}PZI)(ACD)]. (a) Oak Ridge Thermal Ellipsoid Plot (ORTEP) drawing at the 50% probability level. (b) Geometric parameters. See the main text for the definitions of the symbols. (c) Crystallographic packing diagram showing the intermolecular C–H···π bonding interaction (blue dotted line) and the Au···Au distance (red dotted line).

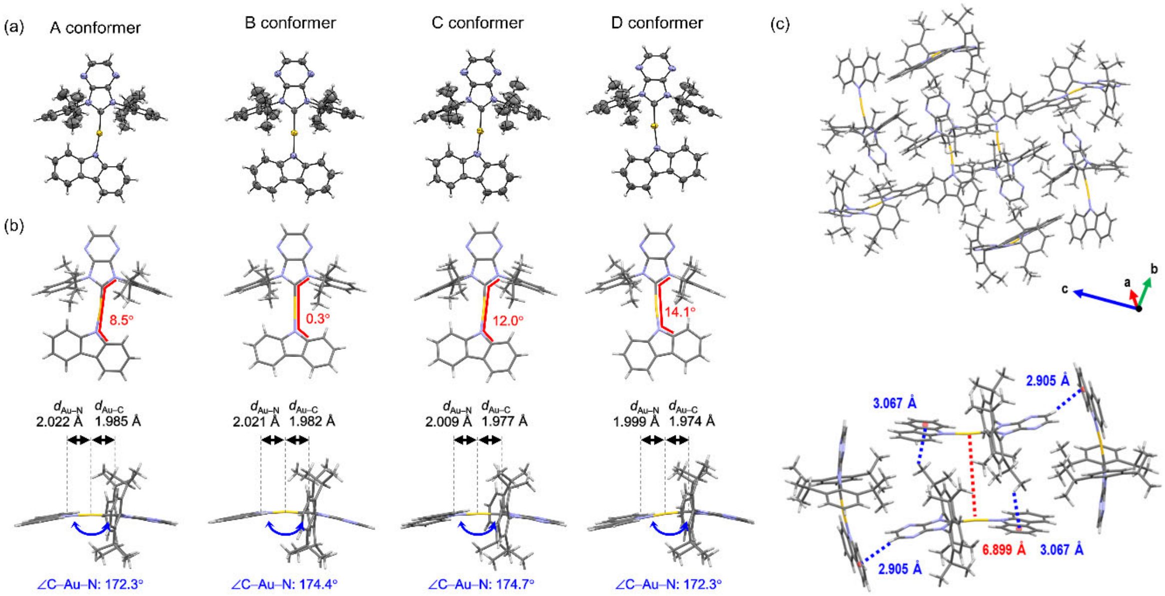

Figure S2. Single crystal X-ray structures of four conformers of [Au(^{Dipp}PZI)(Cz)]. (a) ORTEP drawing at the 50% probability level. (b) Geometric parameters. See the main text for the definitions of the symbols. (c) Crystallographic packing diagram showing the intermolecular C–H···π bonding interaction (blue dotted line) and the Au···Au distance (red dotted line).

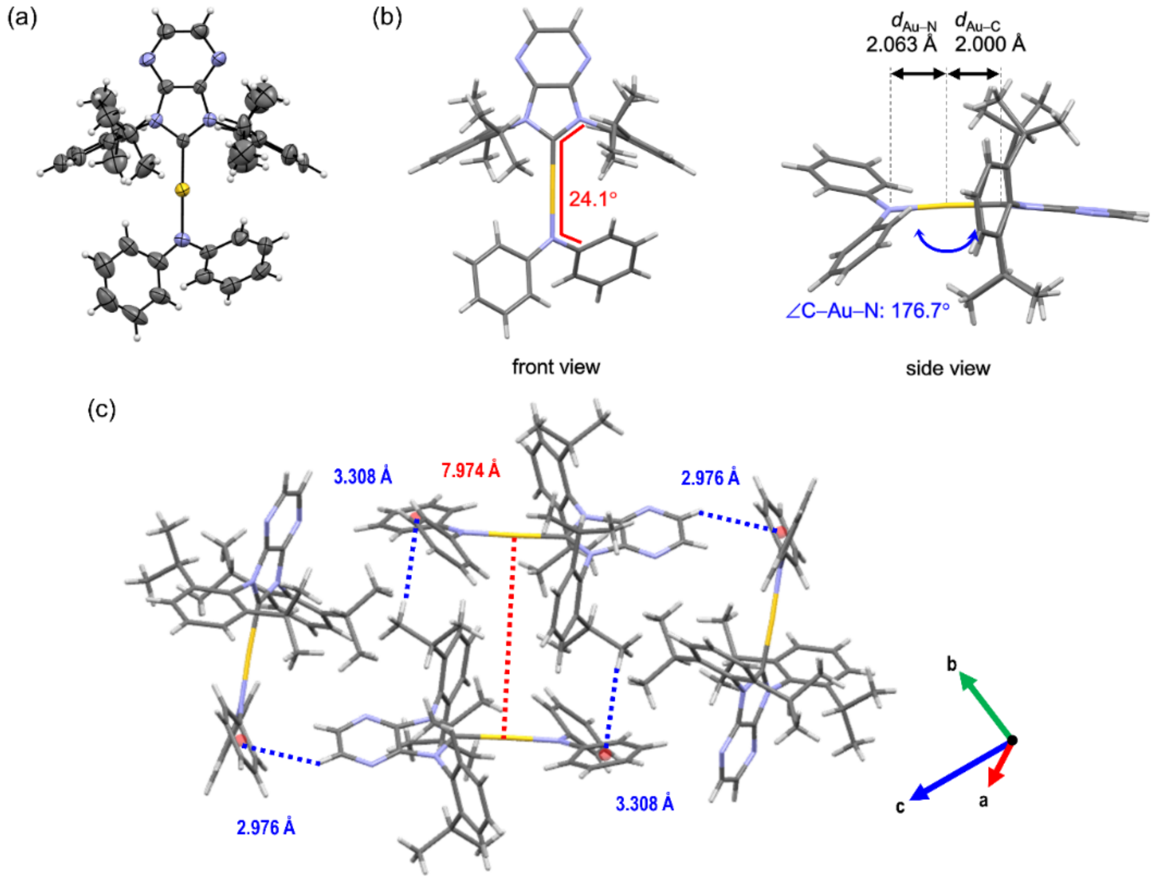

Figure S3. Single crystal X-ray structure of [Au(^{Dipp}PZI)(DPA)]. (a) ORTEP drawing at the 50% probability level. (b) Geometric parameters. See the main text for the definitions of the symbols. (c) Crystallographic packing diagram showing the intermolecular C–H···π bonding interaction (blue dotted line) and the Au···Au distance (red dotted line).

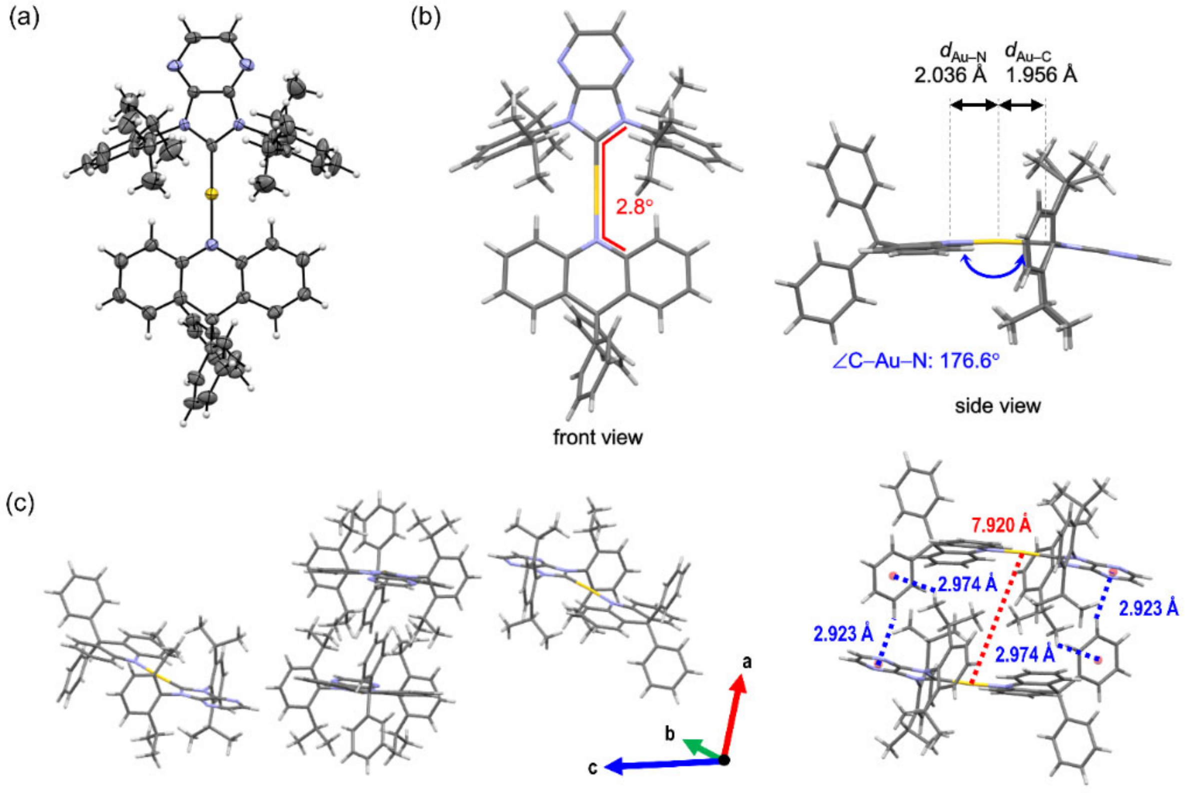

Figure S4. Single crystal X-ray structure of [Au(^{Dipp}PZI)(DPAC)]. (a) ORTEP drawing at the 50% probability level. (b) Geometric parameters. See the main text for the definitions of the symbols. (c) Crystallographic packing diagram showing the intermolecular C–H···π bonding interaction (blue dotted line) and the Au···Au distance (red dotted line).

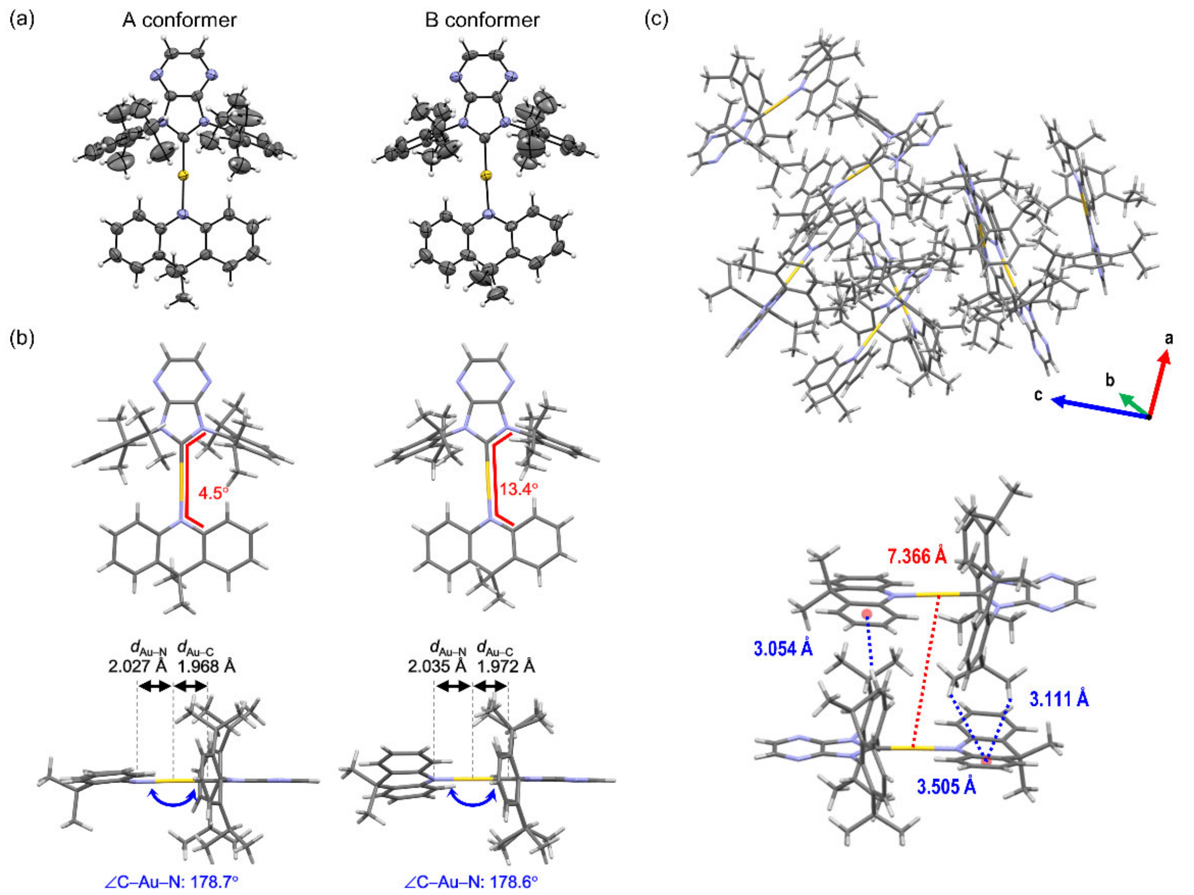

Figure S5. Single crystal X-ray structures of two conformers of [Au(^{Dipp}PZI)(DMAC)]. (a) ORTEP drawing at the 50% probability level. (b) Geometric parameters. See the main text for the definitions of the symbols. (c) Crystallographic packing diagram showing the intermolecular C–H···π bonding interaction (blue dotted line) and the Au···Au distance (red dotted line).

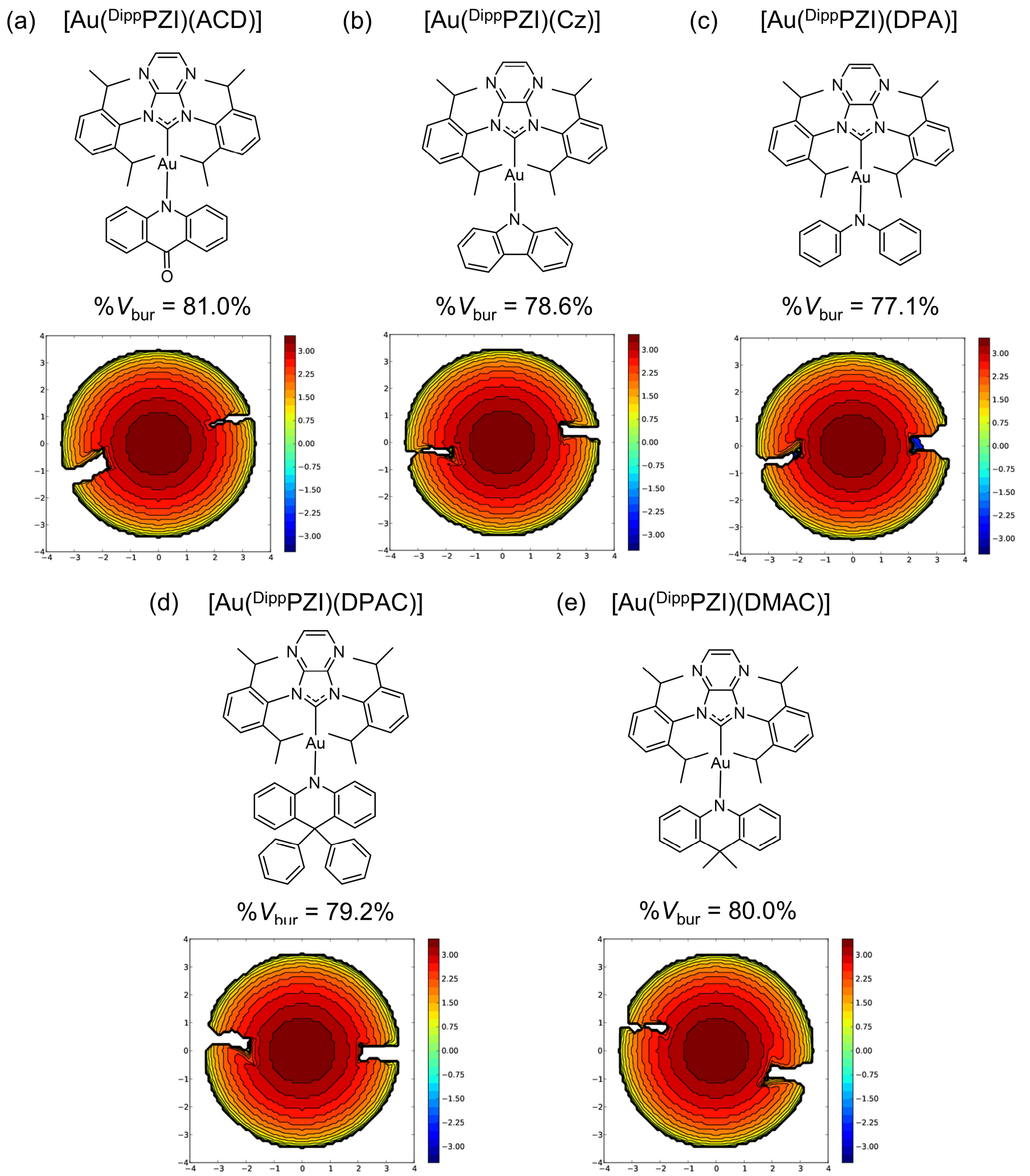

Figure S6. Calculated percent buried volumes (%V_{bur}) and steric maps of the Au(I) complexes (sphere radius = 3.5 Å, Bondi radii scaled by 1.17).

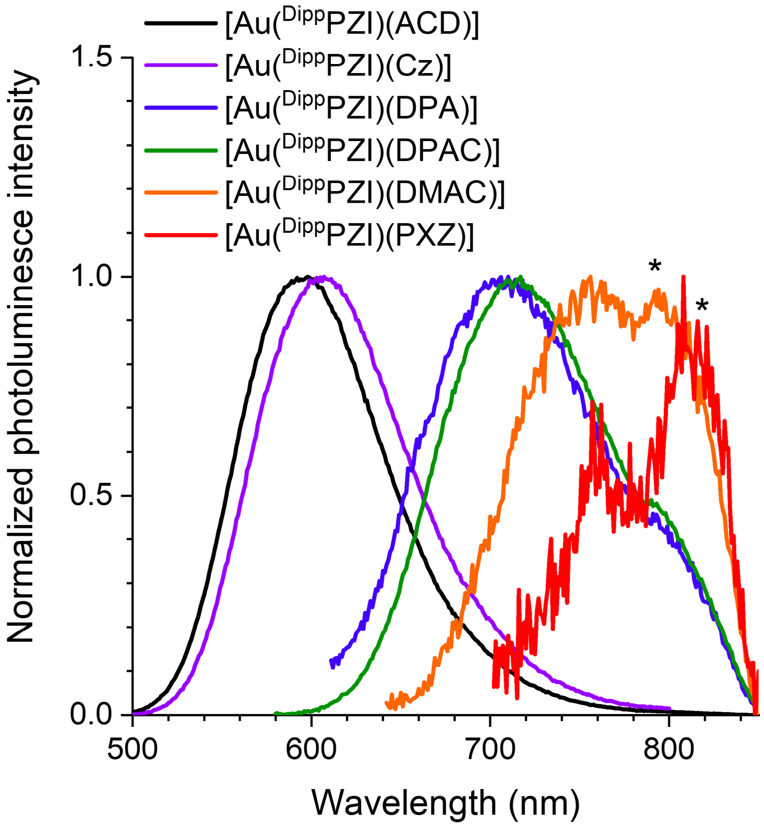

Figure S7. Normalized photoluminescence spectra of 10 μM Au(I) complex in toluene: photoexcitation wavelength (λ_{ex}) = [Au(^{Dipp}PZI)(ACD)], 462 nm; [Au(^{Dipp}PZI)(Cz)], 475 nm; [Au(^{Dipp}PZI)(DPA)], 549 nm; [Au(^{Dipp}PZI)(DPAC)], 560 nm; [Au(^{Dipp}PZI)(DMAC)], 590 nm; [Au(^{Dipp}PZI)(PXZ)], 619 nm. Peaks marked with an aesterisk (*) at wavelengths of 795 and 824 nm are due to instrumental drifts.

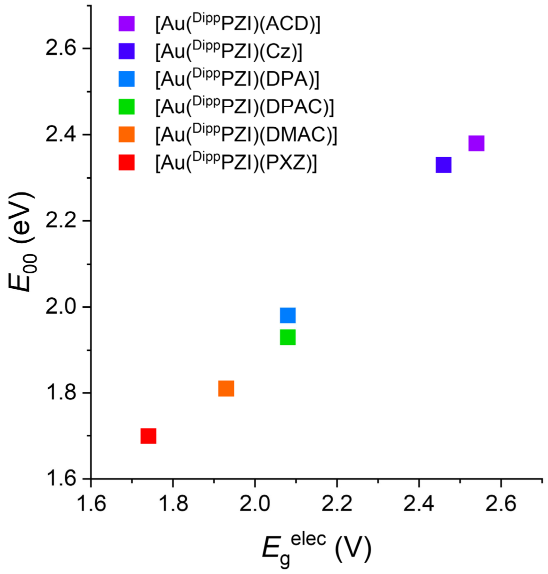

Figure S8. Correlation between E_{00} and the electrochemical bandgap energy (E_g^{elec}) of Au(I) complexes.

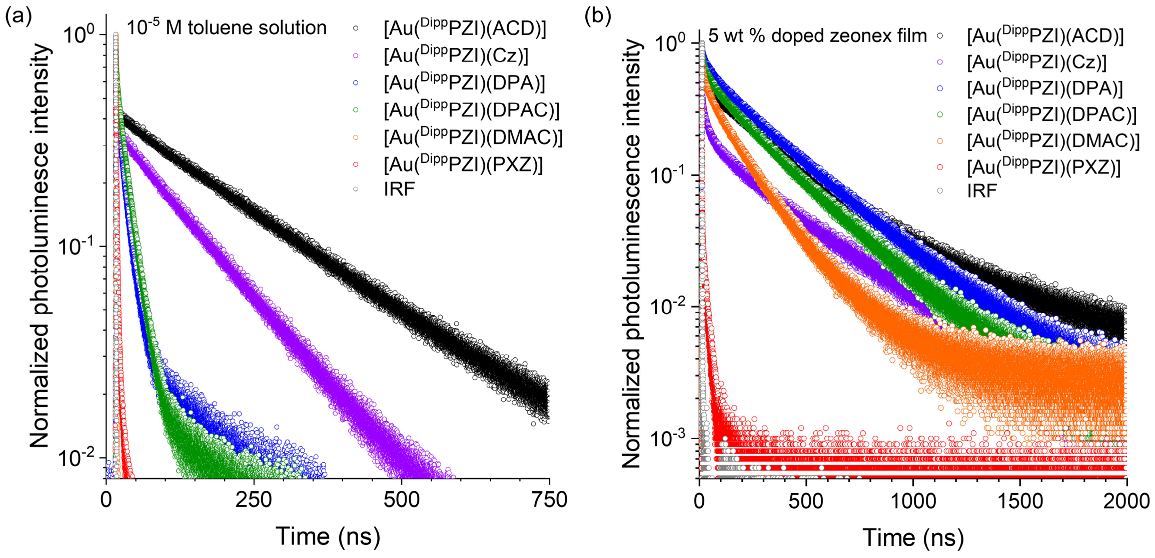

Figure S9. Photoluminescence decay traces of (a) 10 μM Au(I) complexes in deaerated toluene and (b) Zeonex films doped with 5 wt % Au(I) complexes, recorded after 377 nm pulsed laser excitation (time resolution: 16 ps for solutions and 32 ps for polymer films). Photoluminescence decay traces were collected at peak emission wavelengths. Instrumental response functions (IRF) were determined at a wavelength of 377 nm.

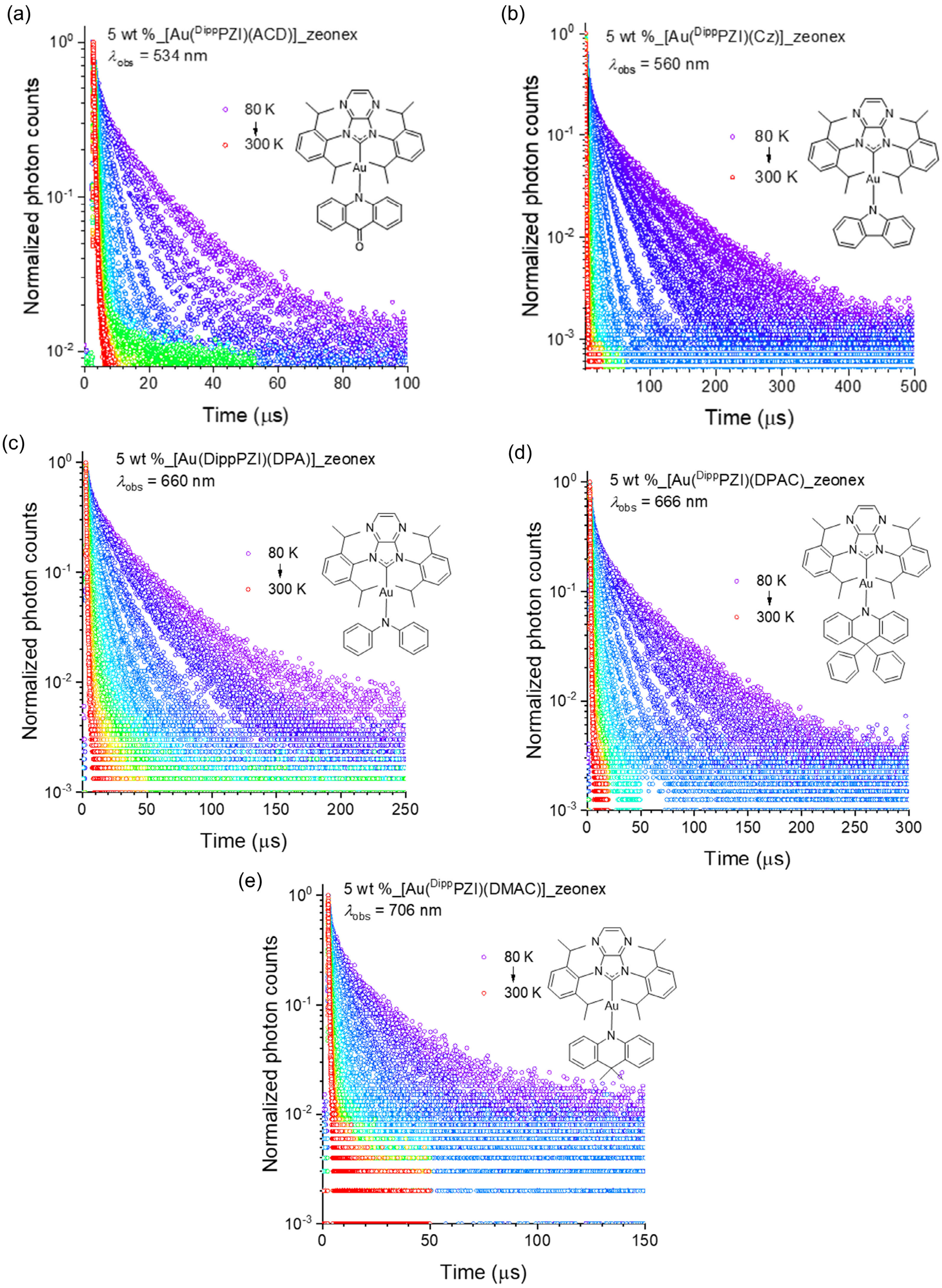

Figure S10. Variable-temperature (80–300 K) photoluminescence decay traces of Zeonex films doped with 5 wt % Au(I) complexes: (a) [Au(^{Dipp}PZI)(ACD)] (observation wavelength (λ_{obs}) = 534 nm), (b) [Au(^{Dipp}PZI)(Cz)] (λ_{obs} = 560 nm), (c) [Au(^{Dipp}PZI)(DPA)] (λ_{obs} = 660 nm), (d) [Au(^{Dipp}PZI)(DPAC)] (λ_{obs} = 666 nm), and (e) [Au(^{Dipp}PZI)(DMAC)] (λ_{obs} = 706 nm). The photoluminescence decay curves were fitted to a triexponential decay model to return a weighted average lifetime (τ_{obs}).

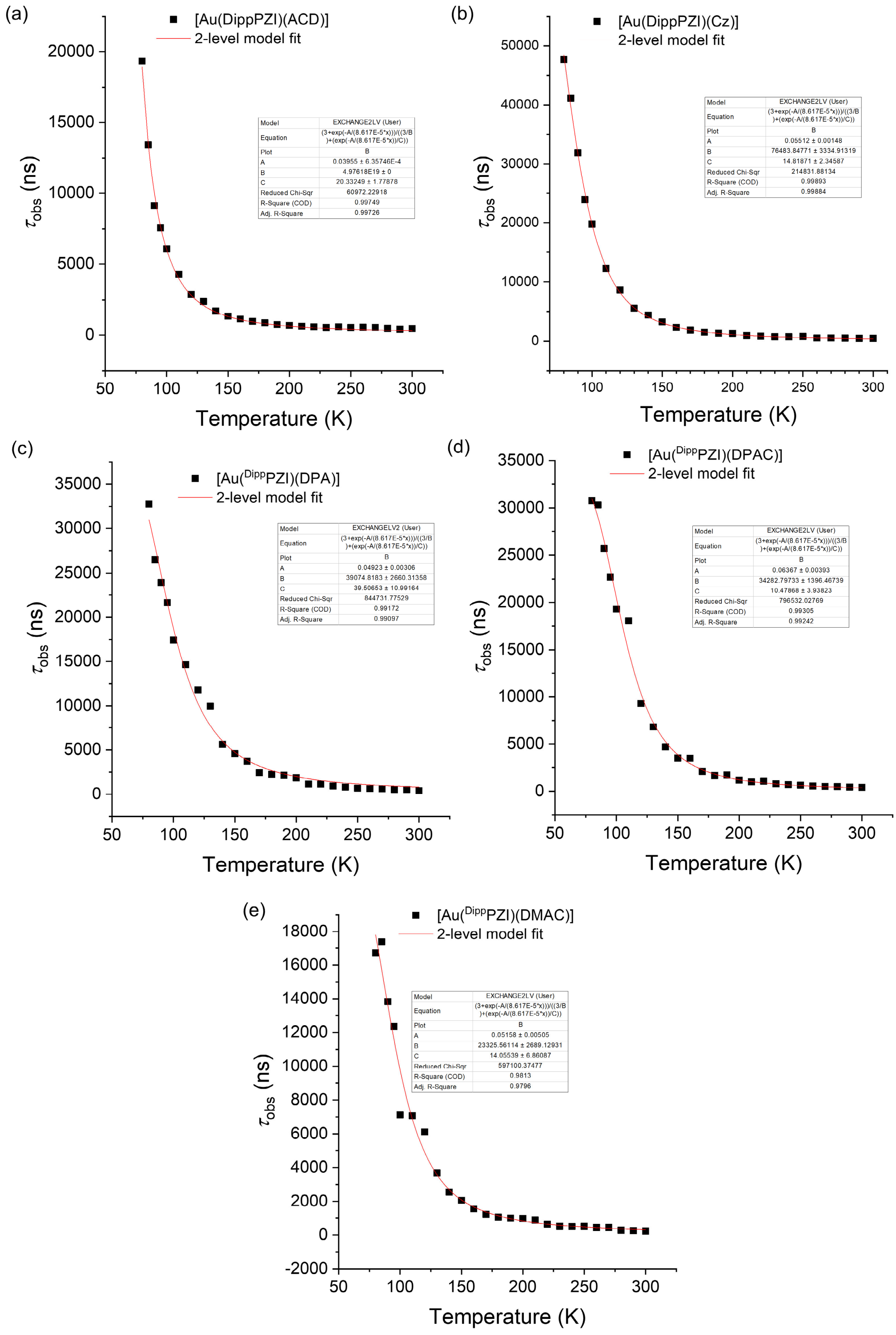

Figure S11. Photoluminescence lifetime (τ_{obs}) of Zeonex films doped with 5 wt % Au(I) complexes as a function of temperature: (a) [Au(^{Dipp}PZI)(ACD)], (b) [Au(^{Dipp}PZI)(Cz)], (c) [Au(^{Dipp}PZI)(DPA)], (d) [Au(^{Dipp}PZI)(DPAC)], and (e) [Au(^{Dipp}PZI)(DMAC)]. Red curves are nonlinear least-squares fits of the data to eq S1.

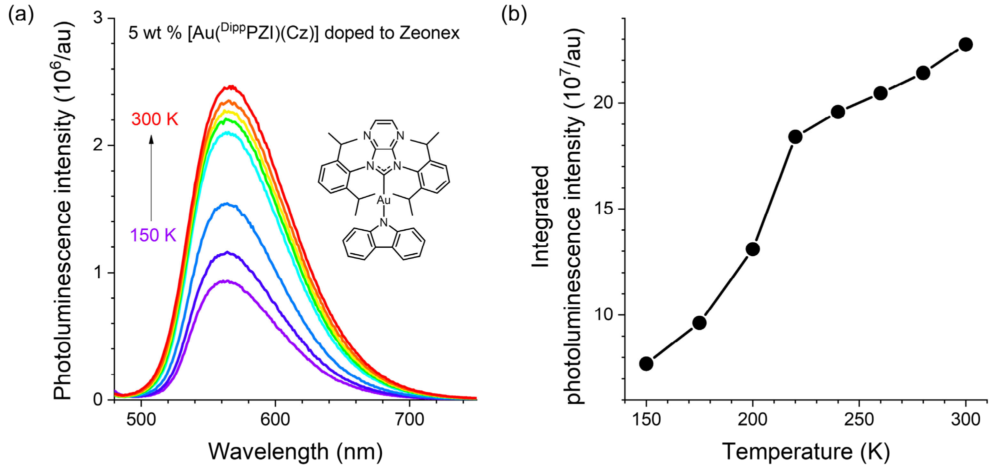

Figure S12. Photoluminescence spectra of Zeonex film doped with 5 wt % [Au(^{Dipp}PZI)(Cz)] at various temperature (150 to 300 K) and integrated photoluminescence intensities as a function of temperature.

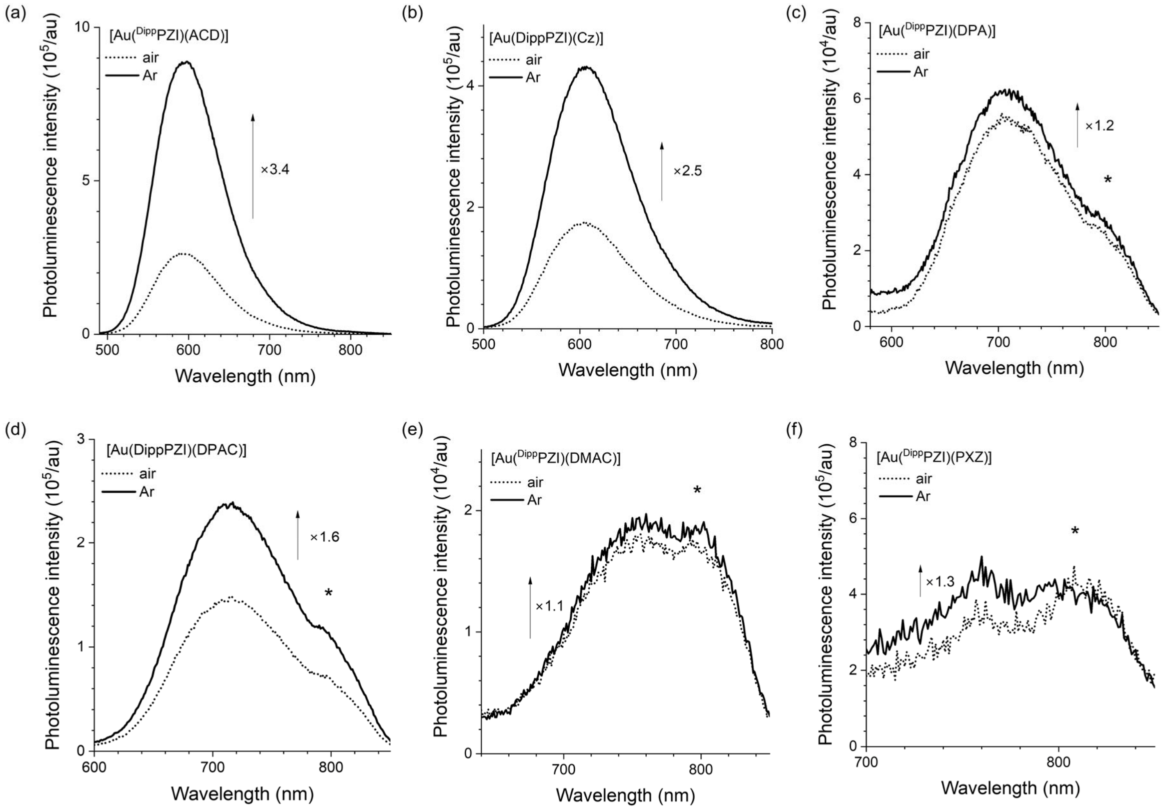

Figure S13. Photoluminescence spectra of the Au(I) complexes (10 μM in toluene) obtained before (dotted line) and after (solid line) deaeration: (a) [Au(^{Dipp}PZI)(ACD)], (b) [Au(^{Dipp}PZI)(Cz)], (c) [Au(^{Dipp}PZI)(DPA)], (d) [Au(^{Dipp}PZI)(DPAC)], (e) [Au(^{Dipp}PZI)(DMAC)], and (f) [Au(^{Dipp}PZI)(PXZ)]. Peaks marked with an aesterisk (*) at wavelengths of 810 nm are due to instrumental drifts.

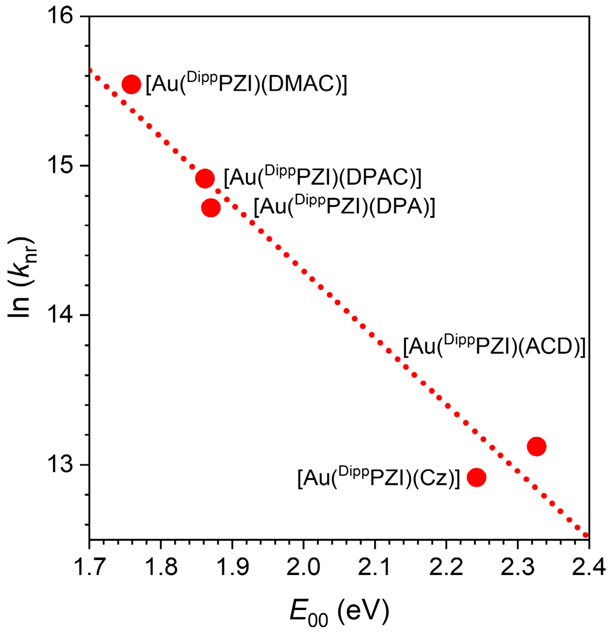

Figure S14. Plot of logarithm of k_{nr} as a function of E_{00} for Au(I) complexes. See Table 1 for the values of k_{nr} and E_{00}. The red dotted line is a visual guidance to the energy gap law of eq 1 in the main text.

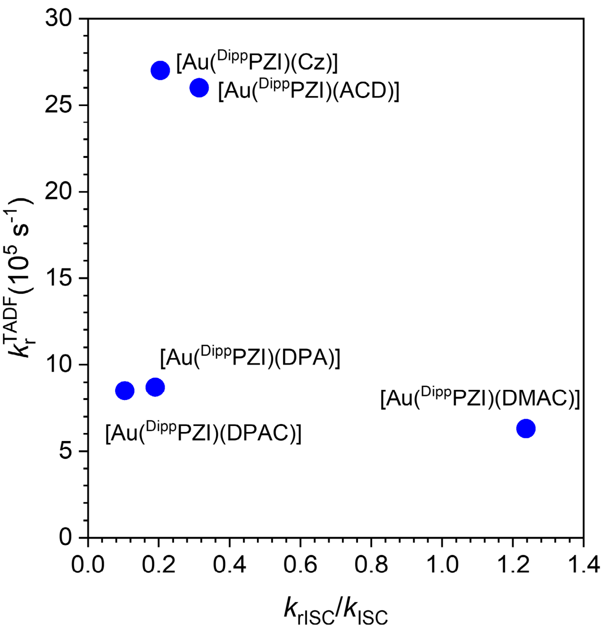

Figure S15. Plot of k_r^{TADF} as a function of the ratio k_{rISC}/k_{ISC} for Au(I) complexes. See Table 1 for the values of k_r^{TADF}, k_{rISC} and k_{ISC}.

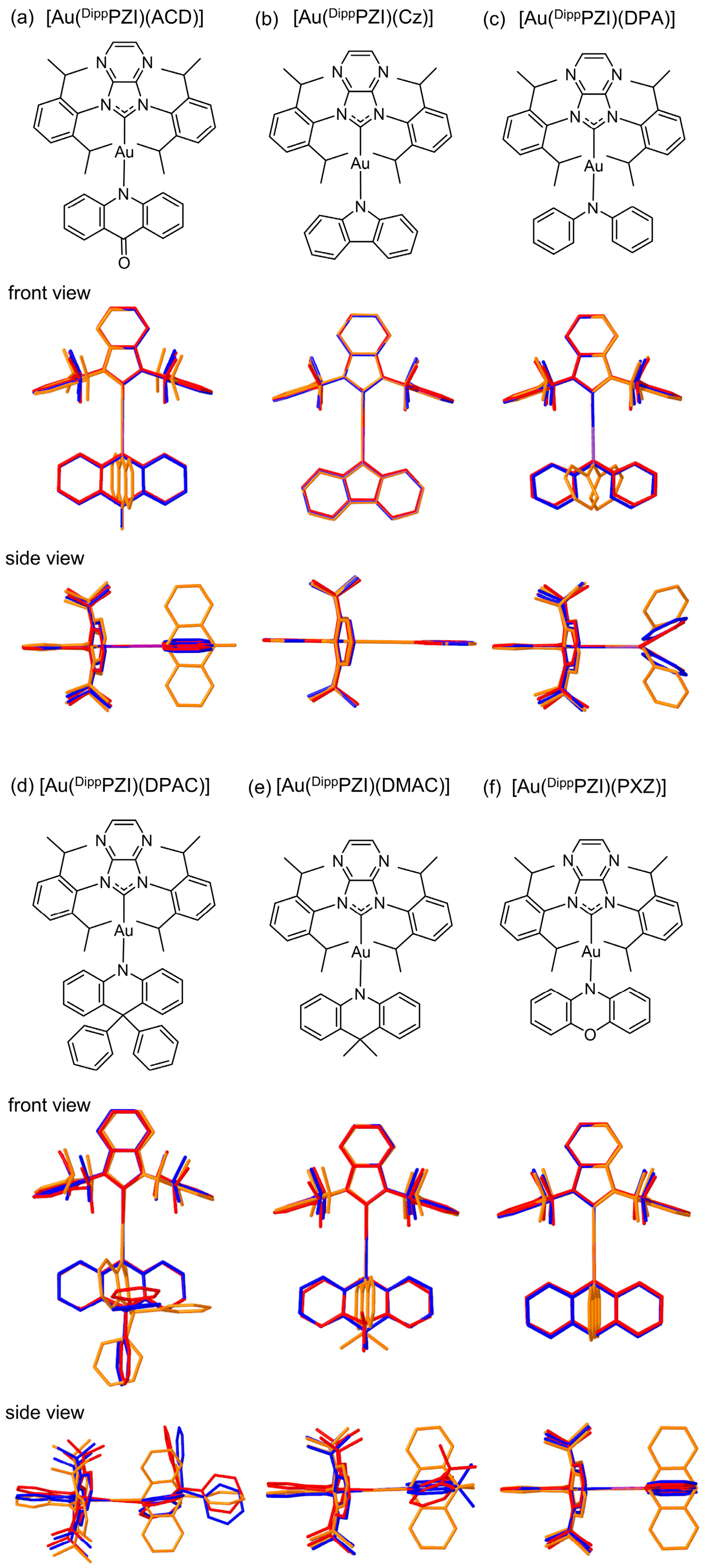

Figure S16. Overlays of the optimized geometries of the S_0 (red), S_1 (orange), and T_1 (blue) states of (a) [Au(^{Dipp}PZI)(ACD)], (b) [Au(^{Dipp}PZI)(Cz)], (c) [Au(^{Dipp}PZI)(DPA)], (d) [Au(^{Dipp}PZI)(DPAC)], (e) [Au(^{Dipp}PZI)(DMAC)], and (f) [Au(^{Dipp}PZI)(PXZ)].

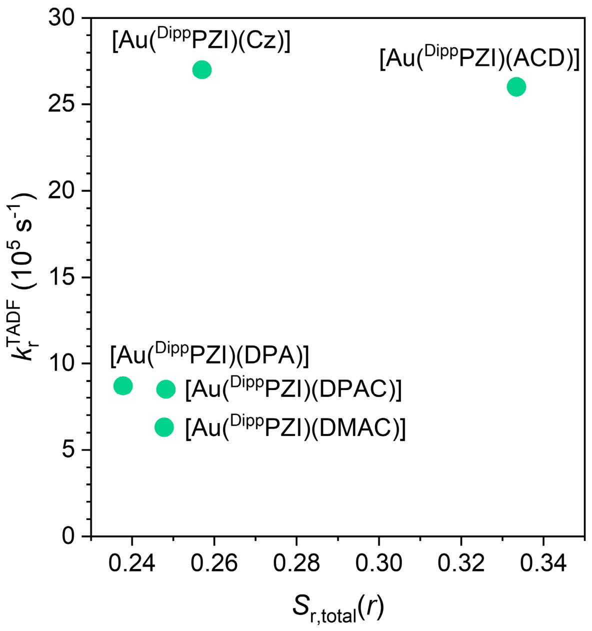

Figure S17. Correlation between k_r^{TADF} and $S_{r,total}(r)$. k_r^{TADF} increases with S_r(r), and then reaches to a plateau.

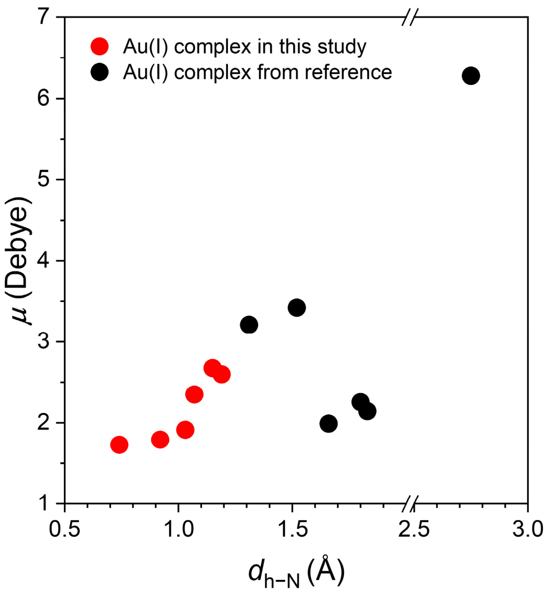

Figure S18. The relation between the transition dipole moment (μ) and d_{h-N} of the Au(I) complexes in this study (red circle) and previous Au(I) complexes (black circles).[2,12,13]

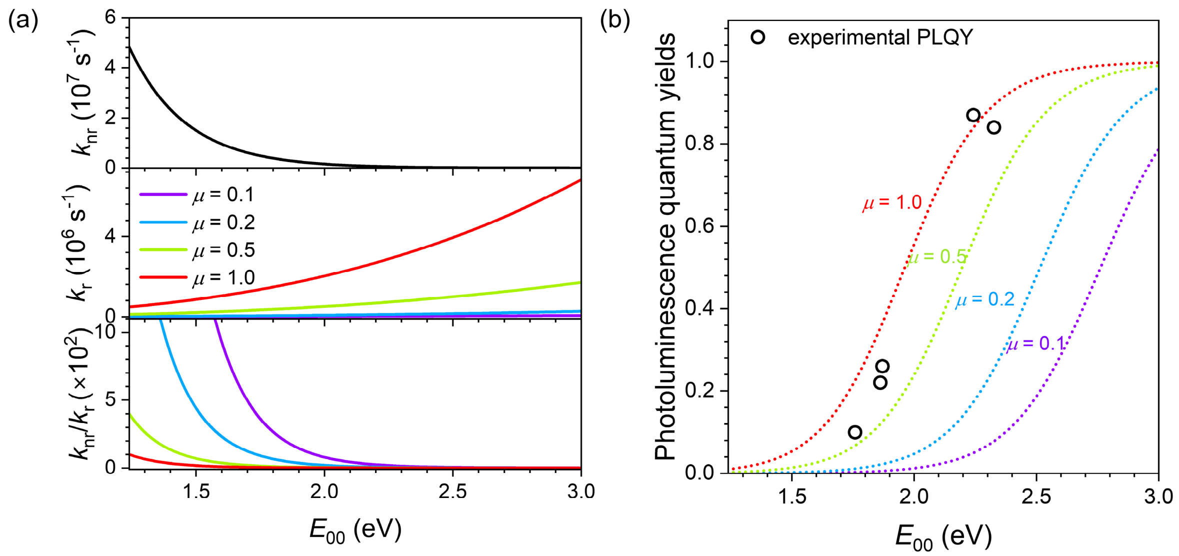

Figure S19. (a) The theoretical plots of predicted k_{nr}, k_r, and k_{nr}/k_r as functions of E_{00}. k_{nr} values were calculated using eq 2 in the main text. k_r values were computed using the equation $k_r/\mu^2 = K_\mu \cdot (n \, / \, 3\pi\varepsilon_0\hbar^4c^3)\cdot E_{00}{}^3$ at various μ, where μ is the transition dipole moment, K_μ is a conversion factor to express the transition dipole moment in Debye (1.11×10^{-59} C^2 m^2 D^{-2}), n is the refractive index for the Zeonex host (1.53), ε_0 is the vacuum permittivity, and c is the speed of light. (b) The predicted photoluminescence quantum yields as functions of E_{00} with different transition dipole moments (μs) (dotted curves), and experimental photoluminescence quantum yields of Au(I) complexes (empty circles).

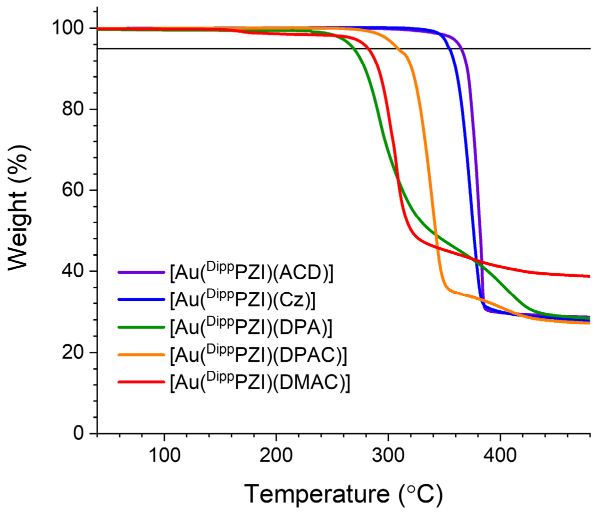

Figure S20. Thermogravimetric analyses of the Au(I) complexes performed at a heating rate of 10 °C min^{-1}. The horizontal line indicates a 5 wt % loss.

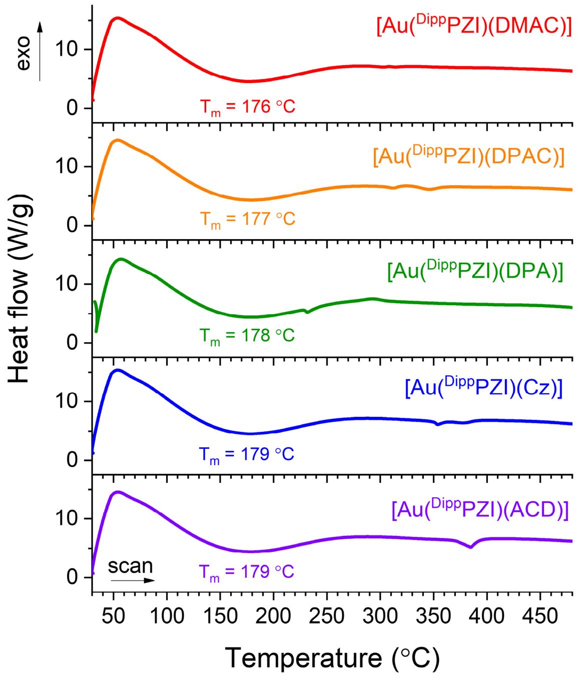

Figure S21. Differntial scanning calorimetry results for Au(I) complexes.

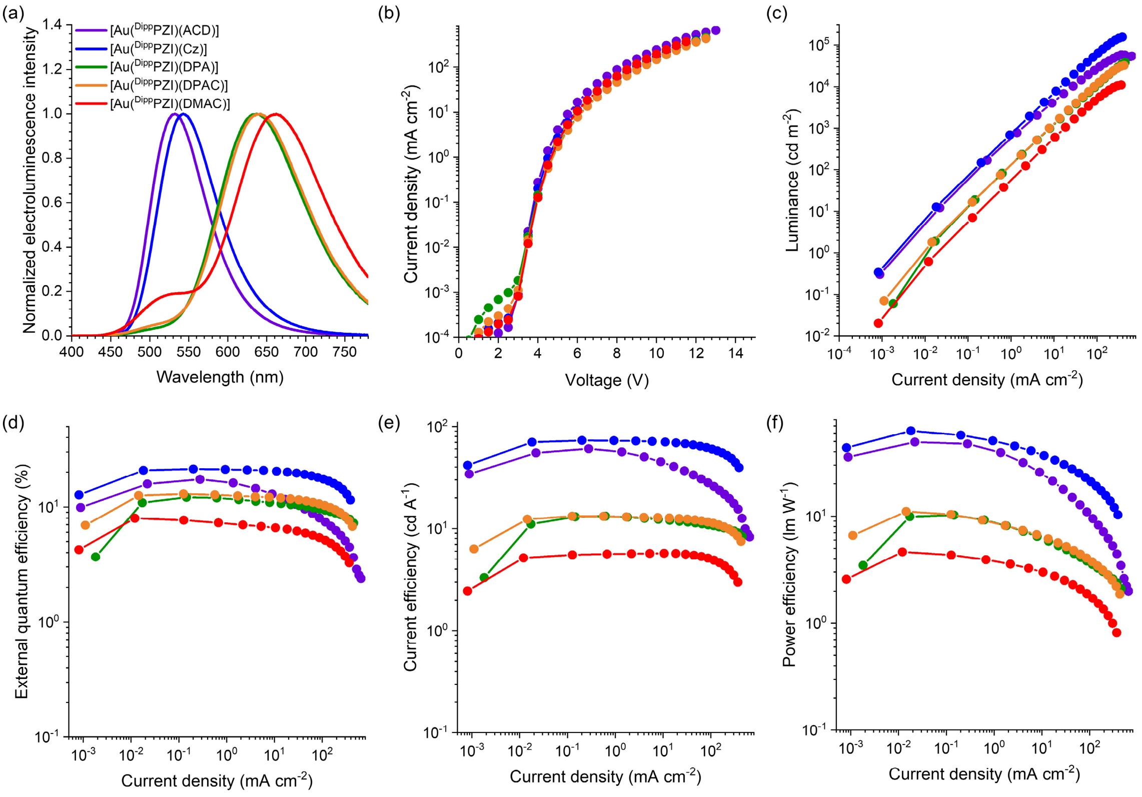

Figure S22. Electroluminescence behaviors of devices involving emitting layers doped with 1% Au(I) complex dopants. (a) Normalized electroluminescence spectra. (b) Current density−voltage curves. (c) Luminance−current density curves. (d) External quantum efficiencies−current density curves. (e) Current efficiency−current density curves. (f) Power efficiency−current density curves.

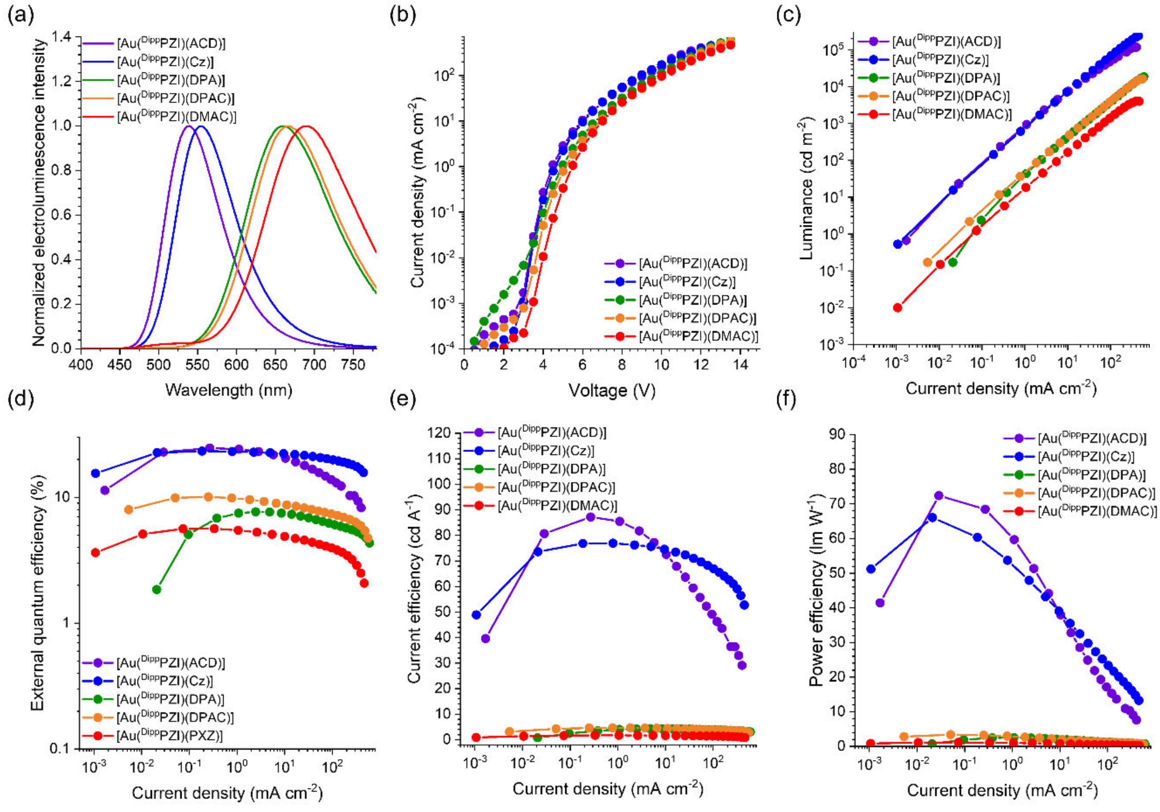

Figure S23. Electroluminescence behaviors of devices involving emitting layers doped with 5% Au(I) complex dopants. (a) Normalized electroluminescence spectra. (b) Current density–voltage curves. (c) Luminance–current density curves. (d) External quantum efficiencies–current density curves. (e) Current efficiency–current density curves. (f) Power efficiency–current density curves.

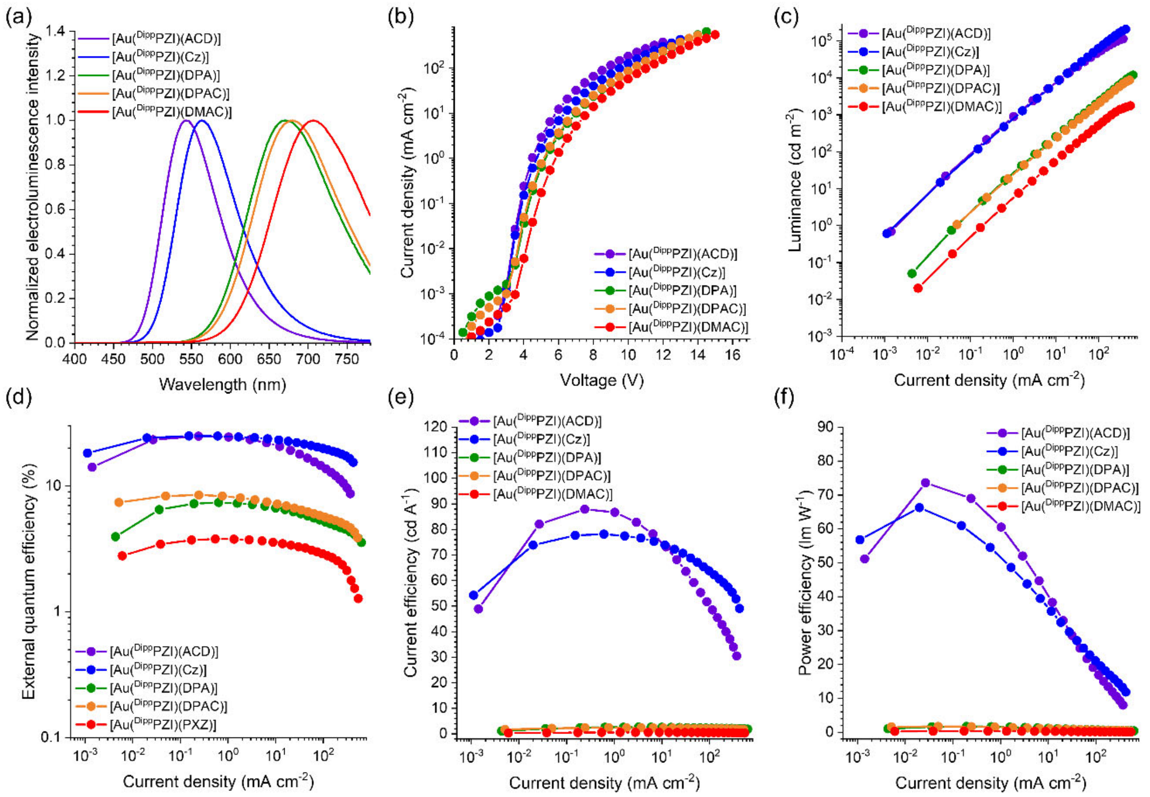

Figure S24. Electroluminescence behaviors of devices involving emitting layers doped with 10% Au(I) complex dopants. (a) Normalized electroluminescence spectra. (b) Current density–voltage curves. (c) Luminance–current density curves. (d) External quantum efficiencies–current density curves. (e) Current efficiency–current density curves. (f) Power efficiency–current density curves.

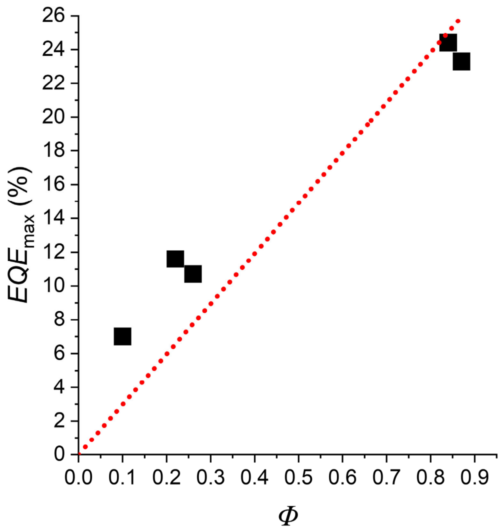

Figure S25. Correlation between EQE_{max} recorded for the devices involving 3% Au(I) complex dopants and Φ of Zeonex films doped with 5 wt % Au(I) complexes.

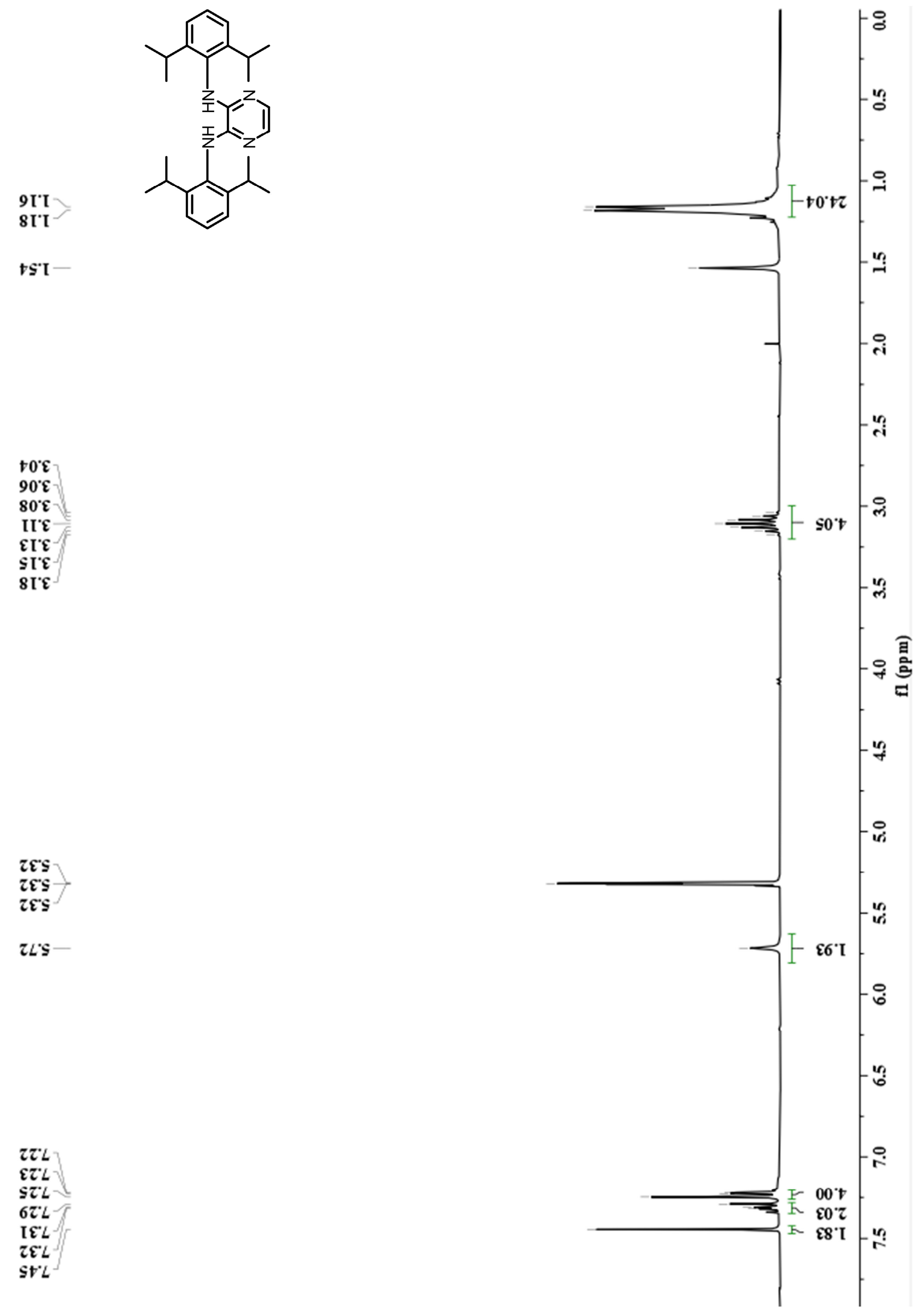

Figure S26. ^{1}H NMR (300 MHz, CD_2Cl_2) spectrum of N^2,N^3-bis(2,6-diisopropylphenyl)pyrazine-2,3-diamine.

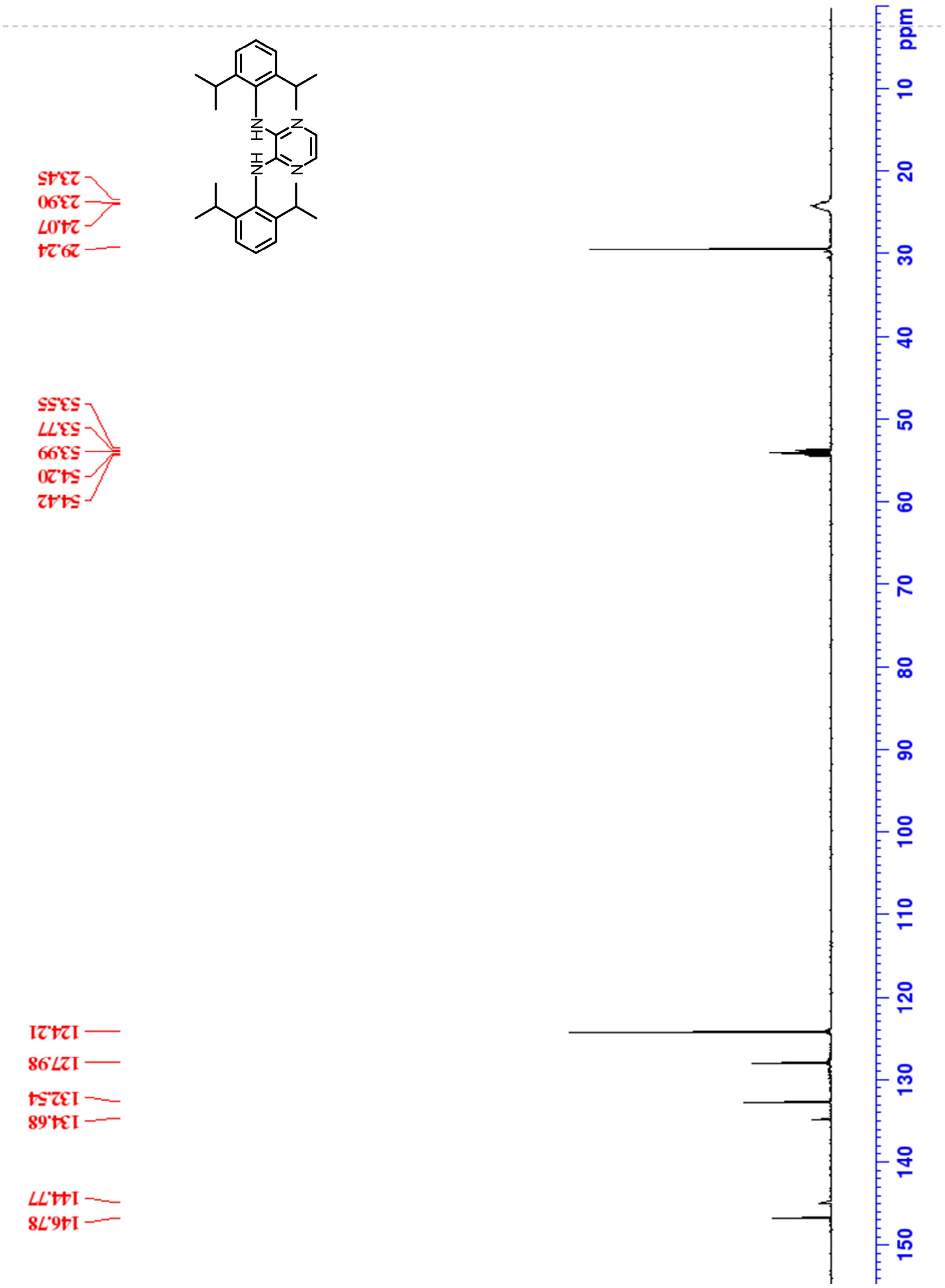

Figure S27. $^{13}C\{^{1}H\}$ NMR (126 MHz, CD_2Cl_2) spectrum of N^2,N^3-bis(2,6-diisopropylphenyl)pyrazine-2,3-diamine.

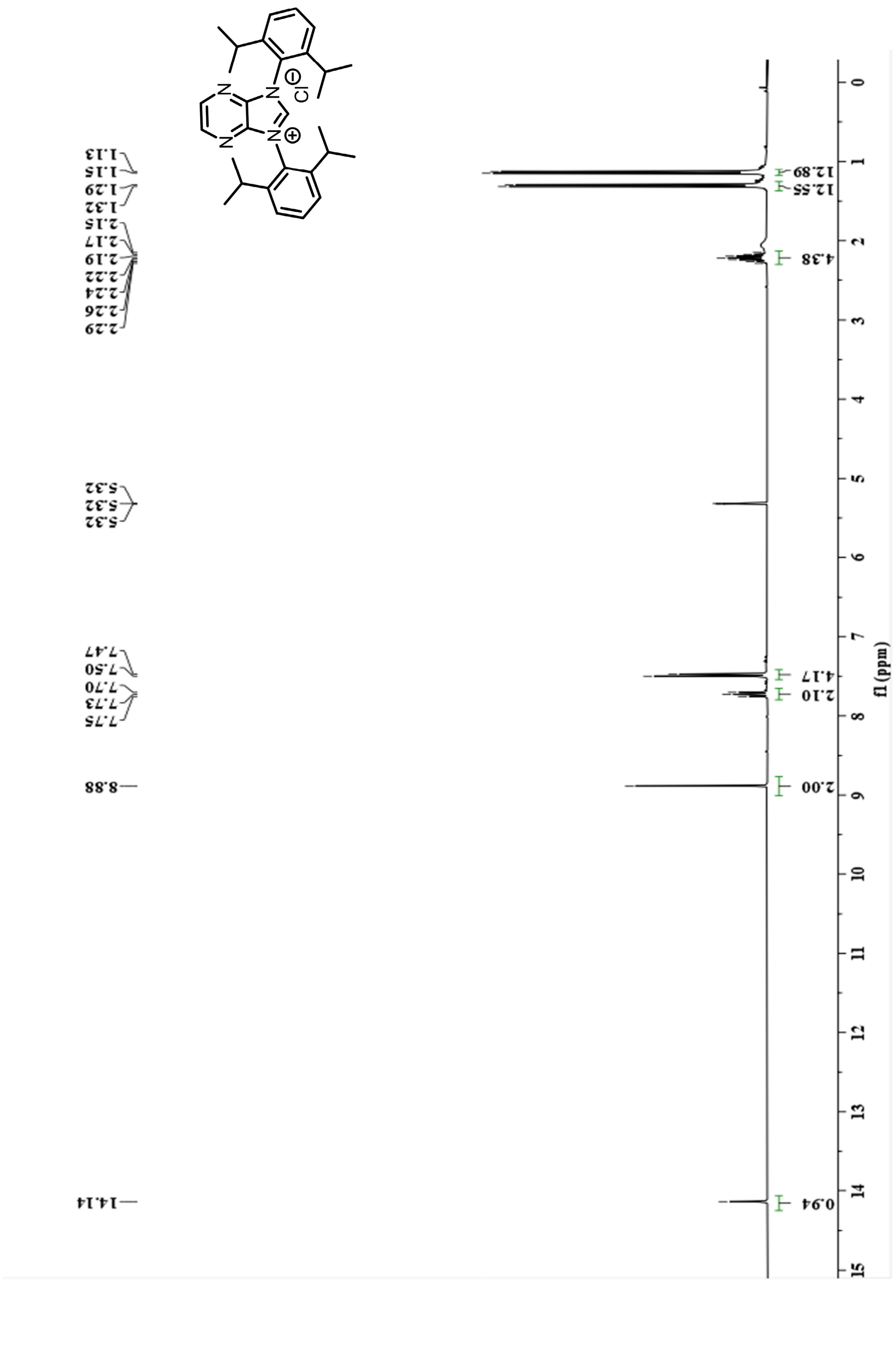

Figure S28. ^{1}H NMR (300 MHz, CD_2Cl_2) spectrum of 1,3-bis(2,6-diisopropylphenyl)-1*H*-imidazo[4,5-*b*]pyrazine-3-ium chloride.

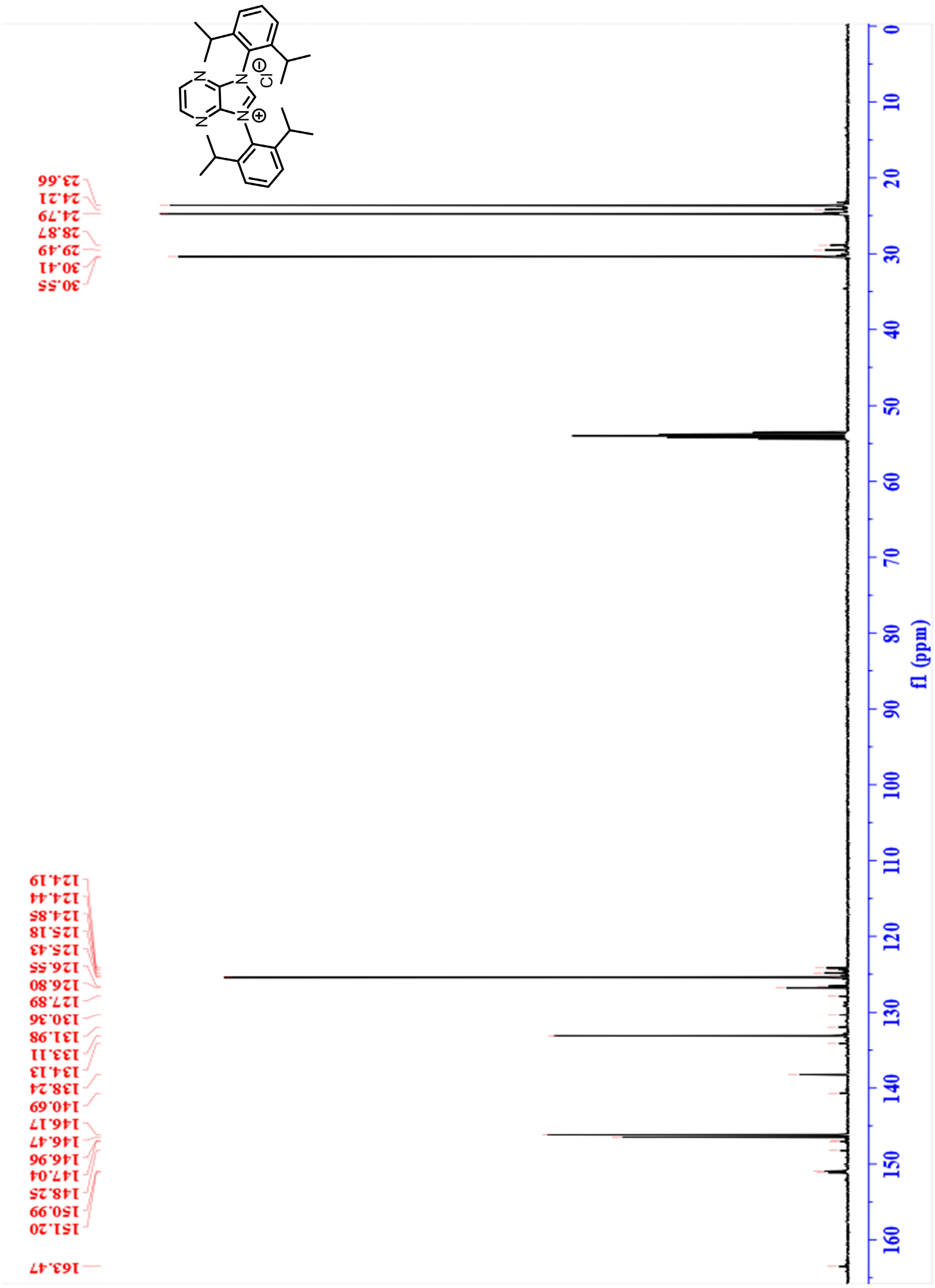

Figure S29. $^{13}C\{^{1}H\}$ NMR (126 MHz, CD_2Cl_2) spectrum of 1,3-bis(2,6-diisopropylphenyl)-1*H*-imidazo[4,5-*b*]pyrazine-3-ium chloride.

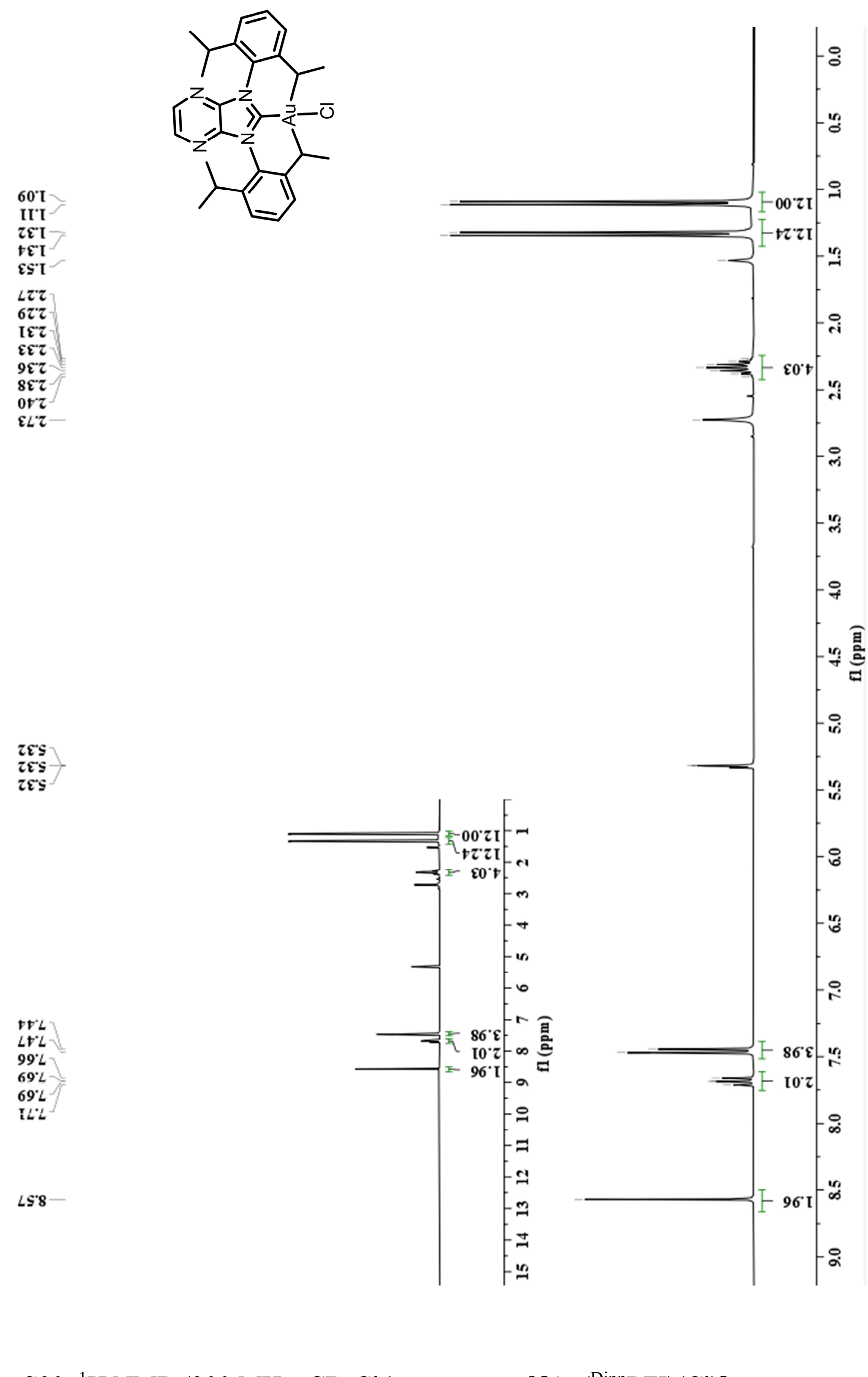

Figure S30. ^{1}H NMR (300 MHz, CD_2Cl_2) spectrum of [Au(^{Dipp}PZI)(Cl)].

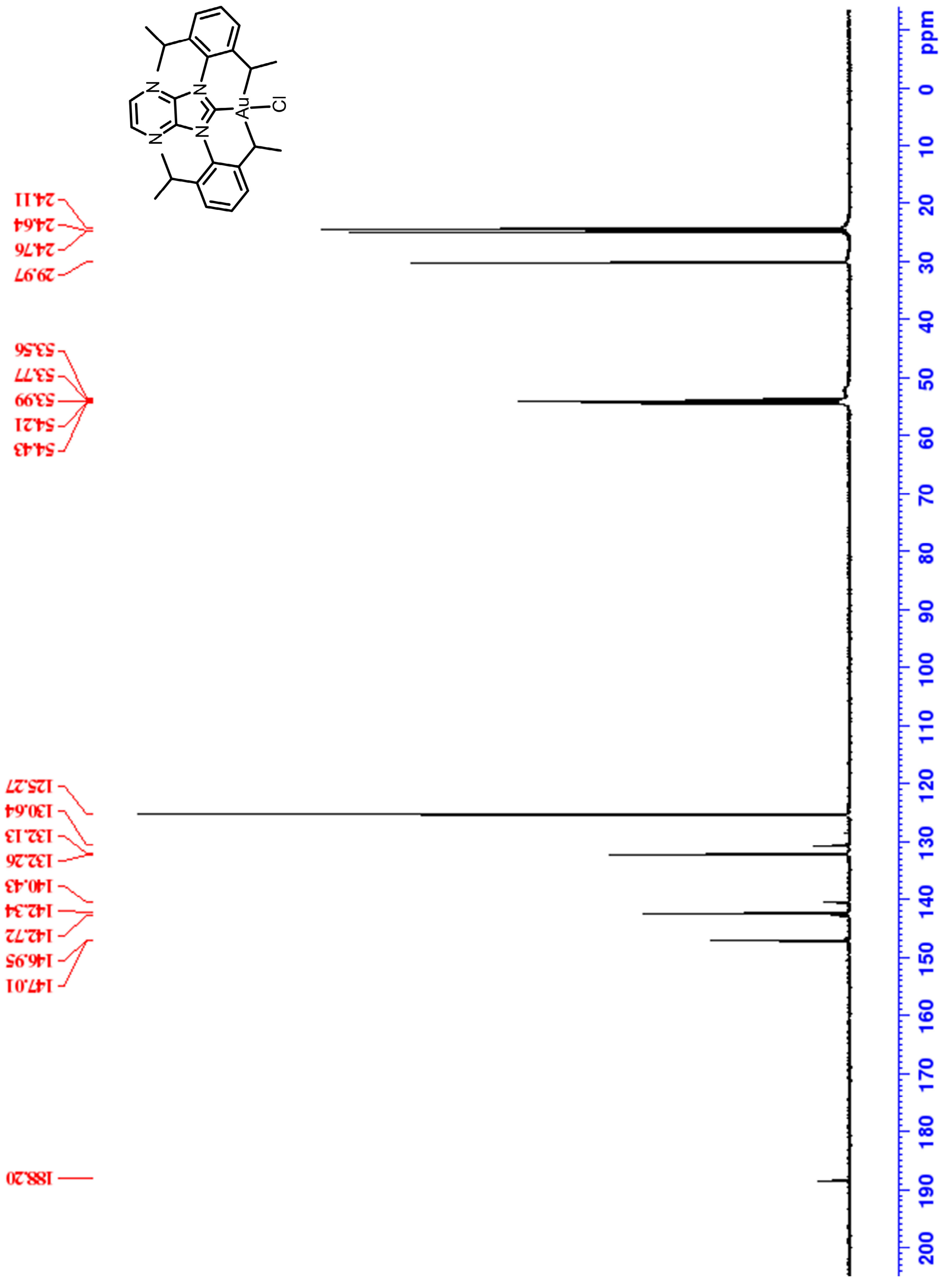

Figure S31. $^{13}C\{^{1}H\}$ NMR (126 MHz, CD_2Cl_2) spectrum of [Au(^{Dipp}PZI)(Cl)].

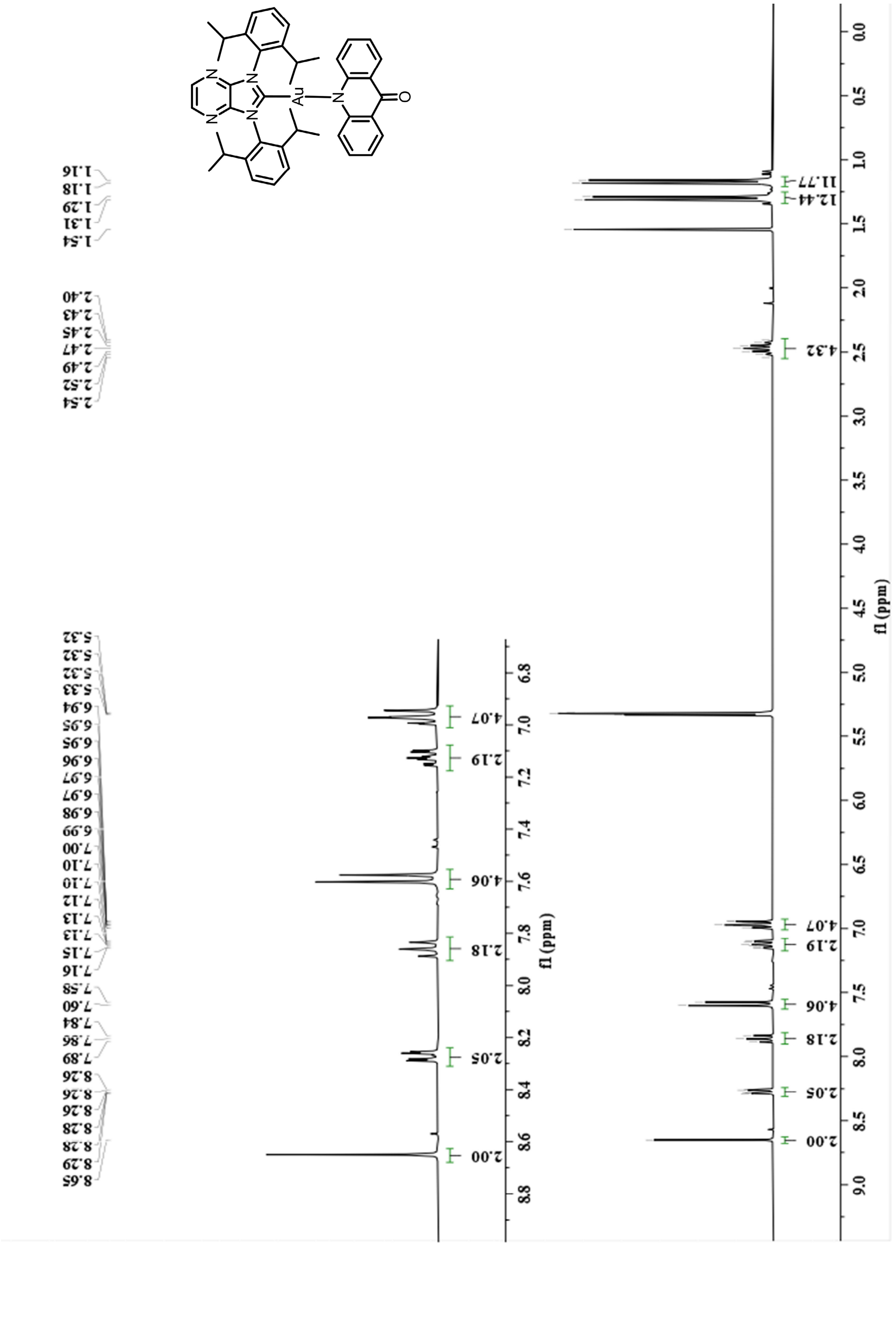

Figure S32. ^{1}H NMR (300 MHz, CD_2Cl_2) spectrum of [Au(^{Dipp}PZI)(ACD)].

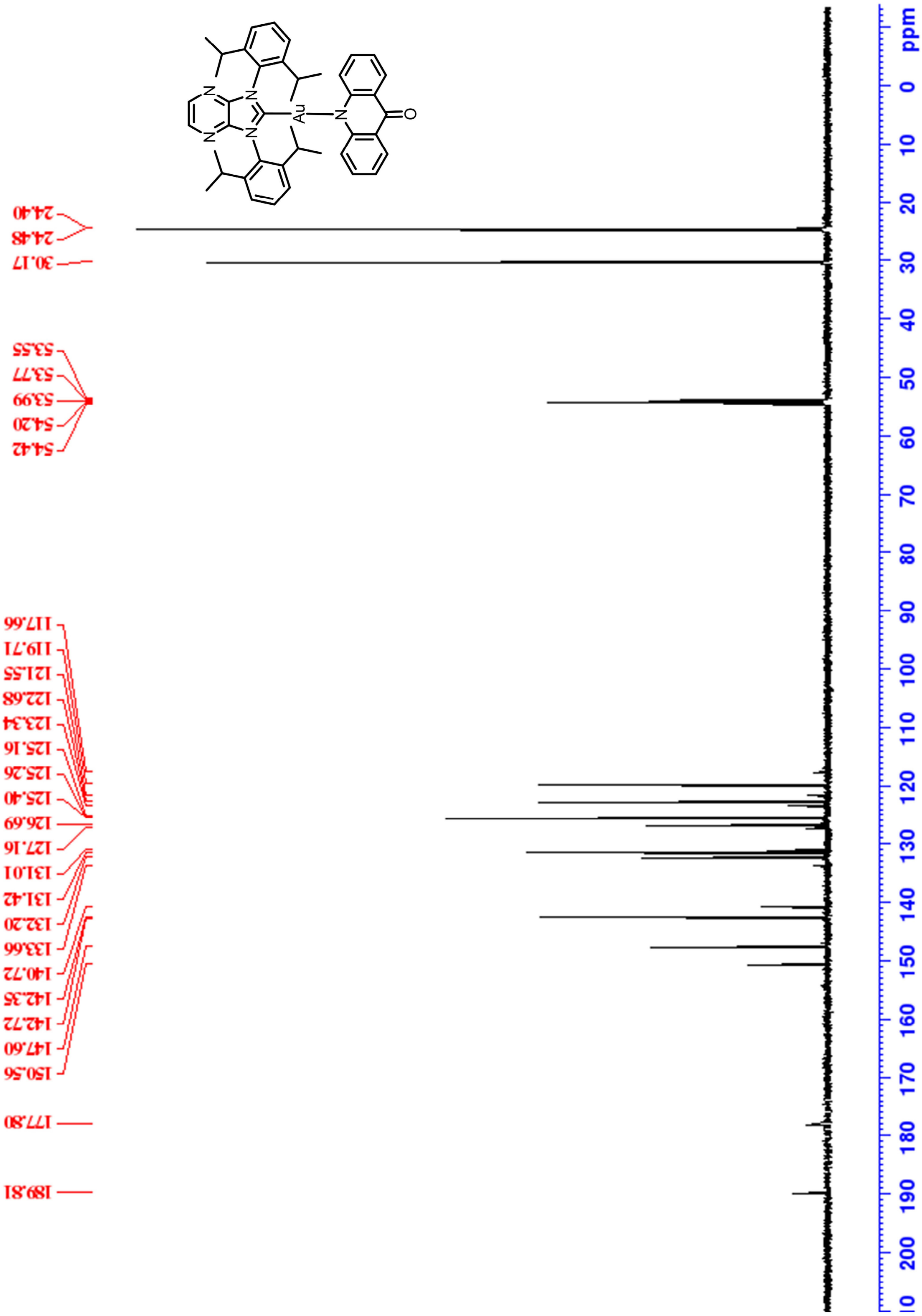

Figure S33. $^{13}C\{^{1}H\}$ NMR (126 MHz, CD_2Cl_2) spectrum of [Au(^{Dipp}PZI)(ACD)].

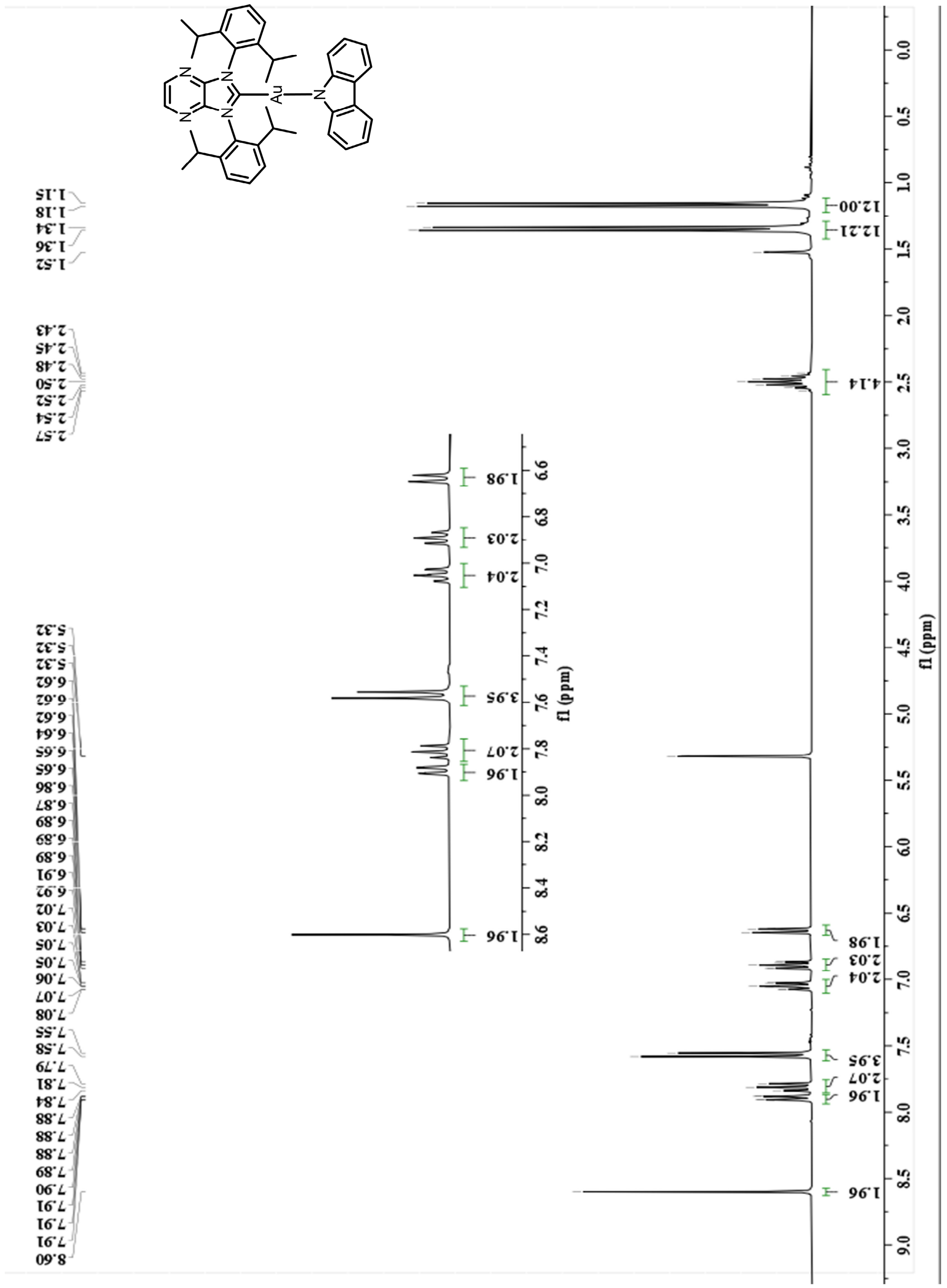

Figure S34. ^{1}H NMR (300 MHz, CD_2Cl_2) spectrum of [Au(^{Dipp}PZI)(Cz)].

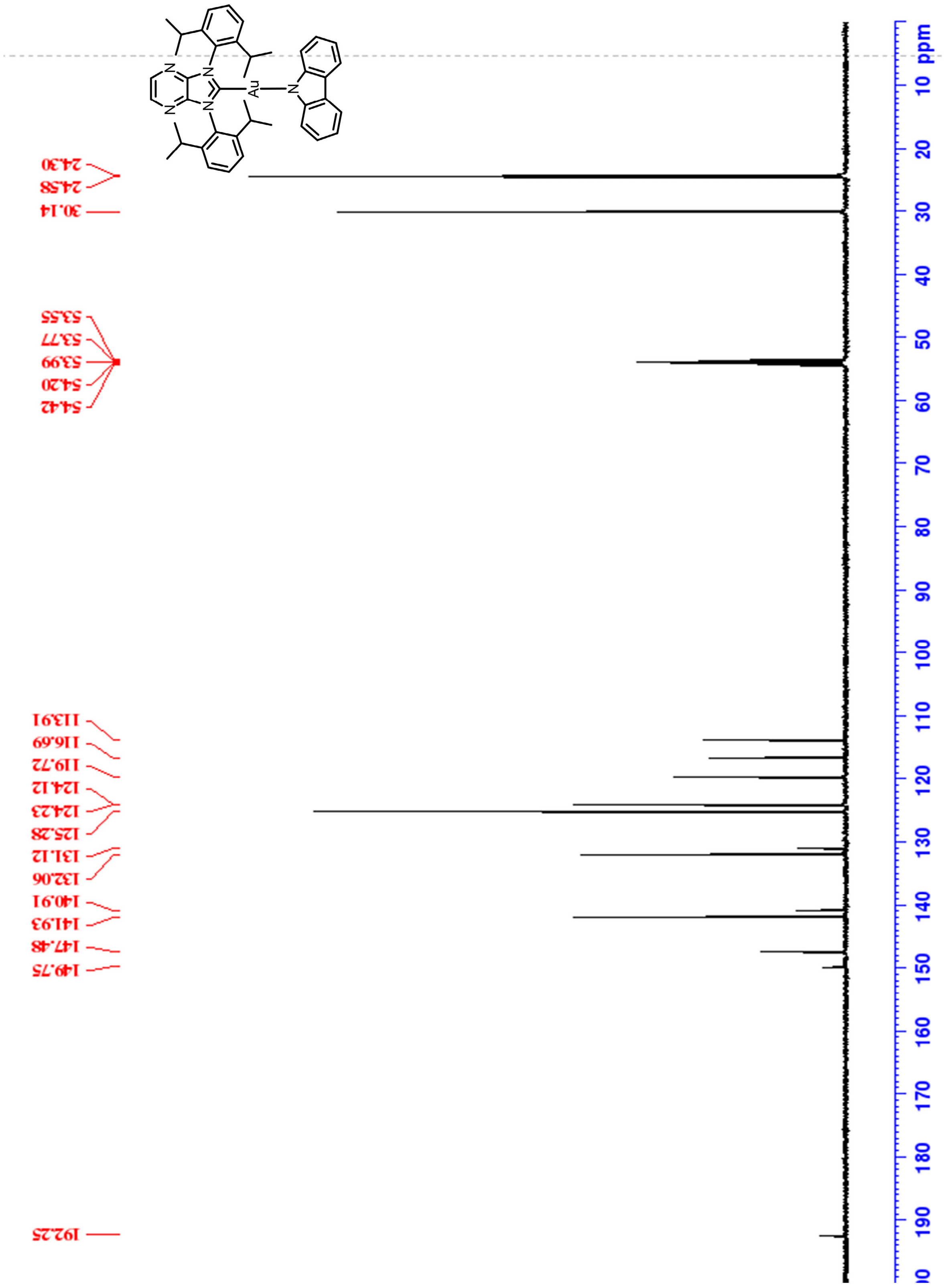

Figure S35. $^{13}C\{^{1}H\}$ NMR (126 MHz, CD_2Cl_2) spectrum of [Au(^{Dipp}PZI)(Cz)].

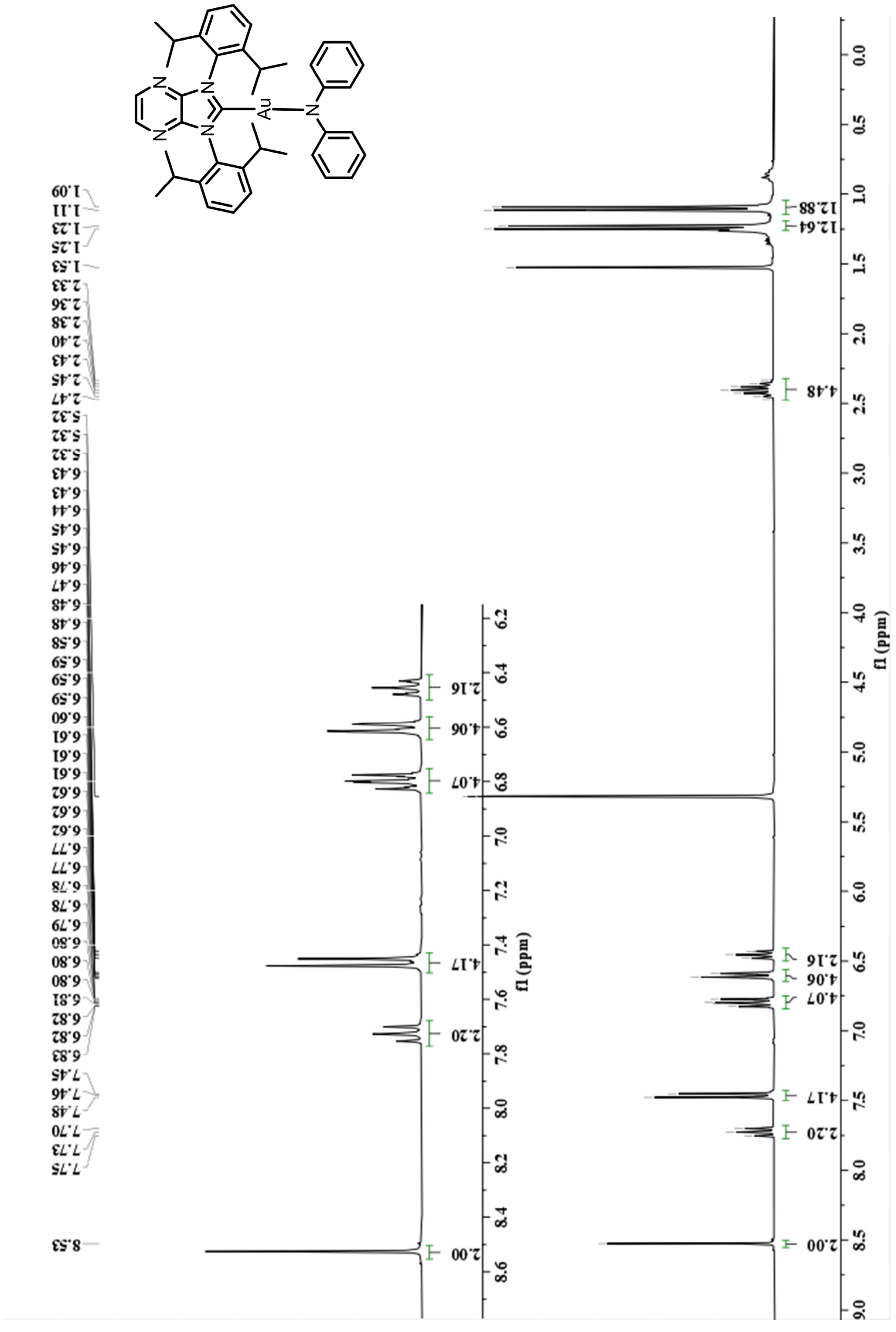

Figure S36. ^{1}H NMR (300 MHz, CD_2Cl_2) spectrum of [Au(^{Dipp}PZI)(DPA)].

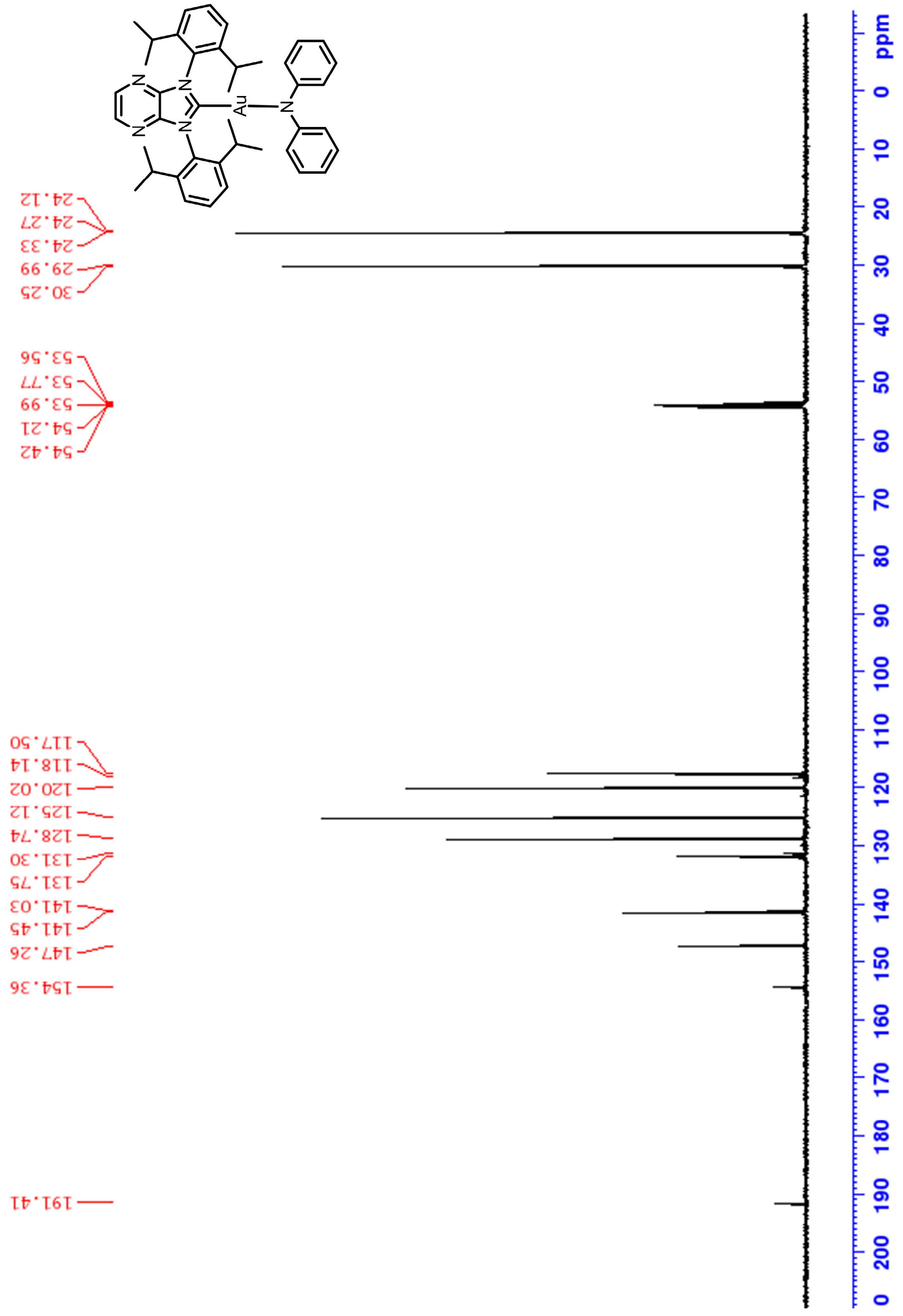

Figure S37. $^{13}C\{^{1}H\}$ NMR (126 MHz, CD_2Cl_2) spectrum of [Au(^{Dipp}PZI)(DPA)].

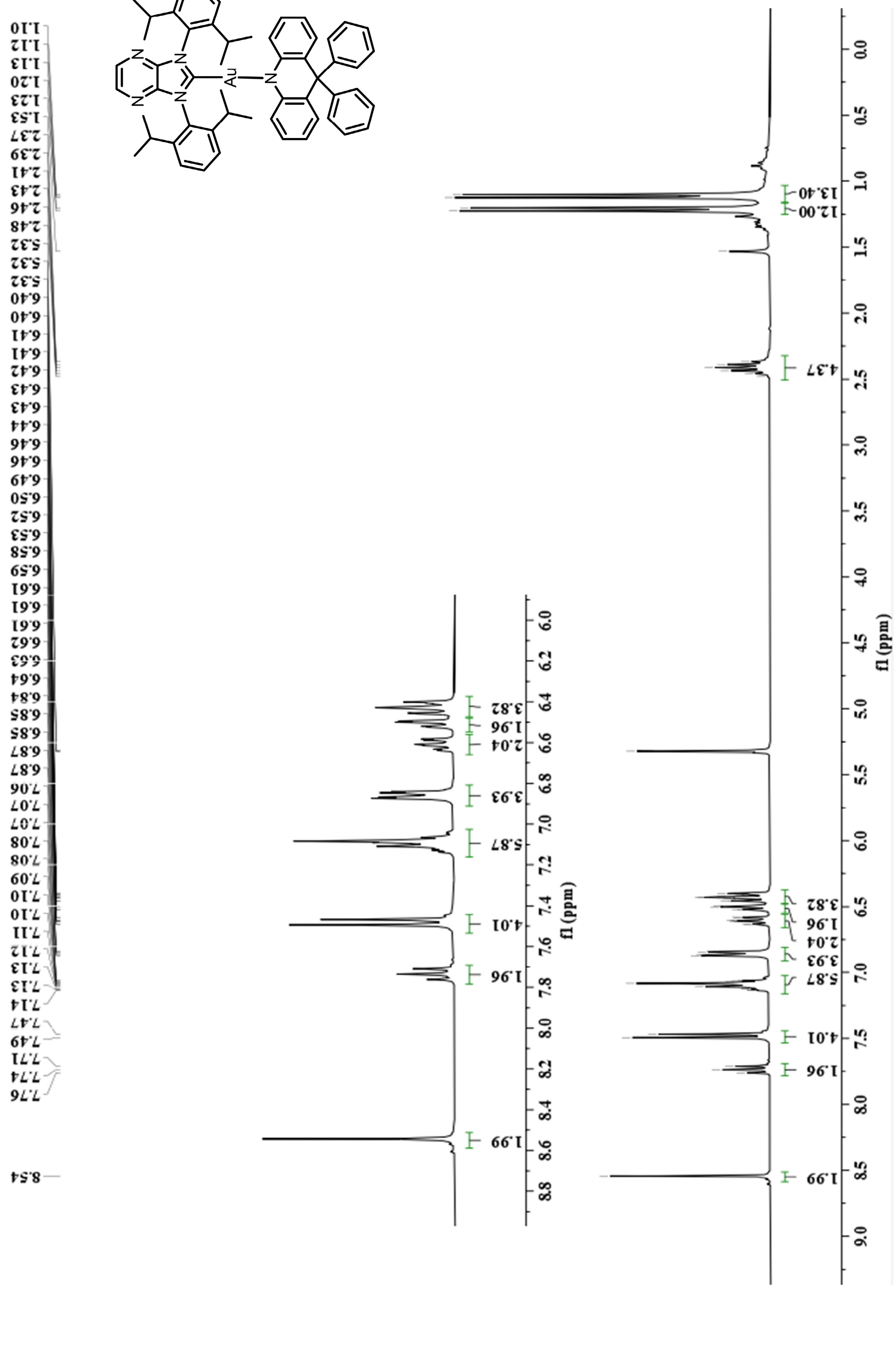

Figure S38. ^{1}H NMR (300 MHz, CD_2Cl_2) spectrum of [Au(^{Dipp}PZI)(DPAC)].

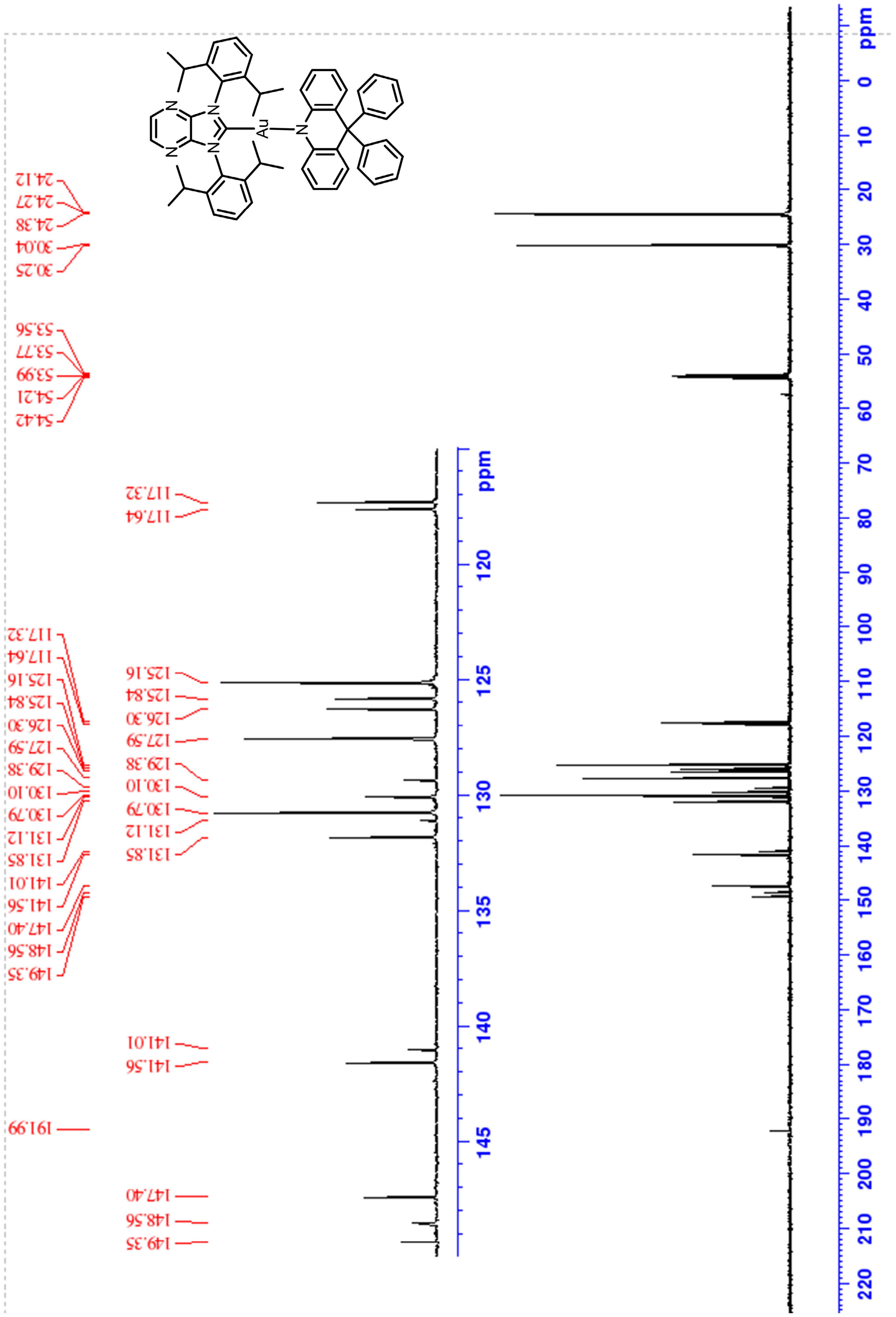

Figure S39. $^{13}C\{^{1}H\}$ NMR (126 MHz, CD_2Cl_2) spectrum of [Au(^{Dipp}PZI)(DPAC)].

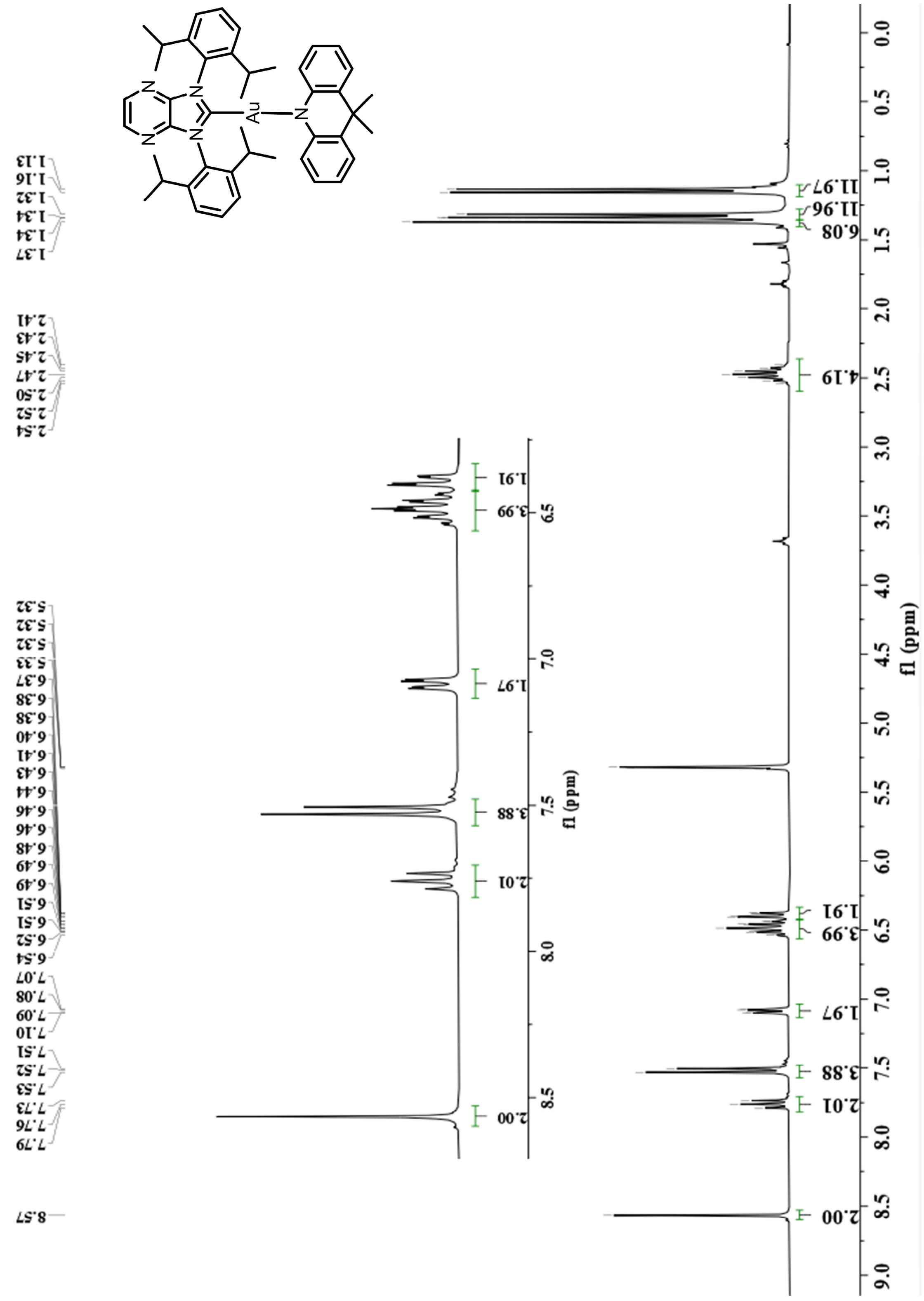

Figure S40. ^{1}H NMR (300 MHz, CD_2Cl_2) spectrum of [Au(^{Dipp}PZI)(DMAC)].

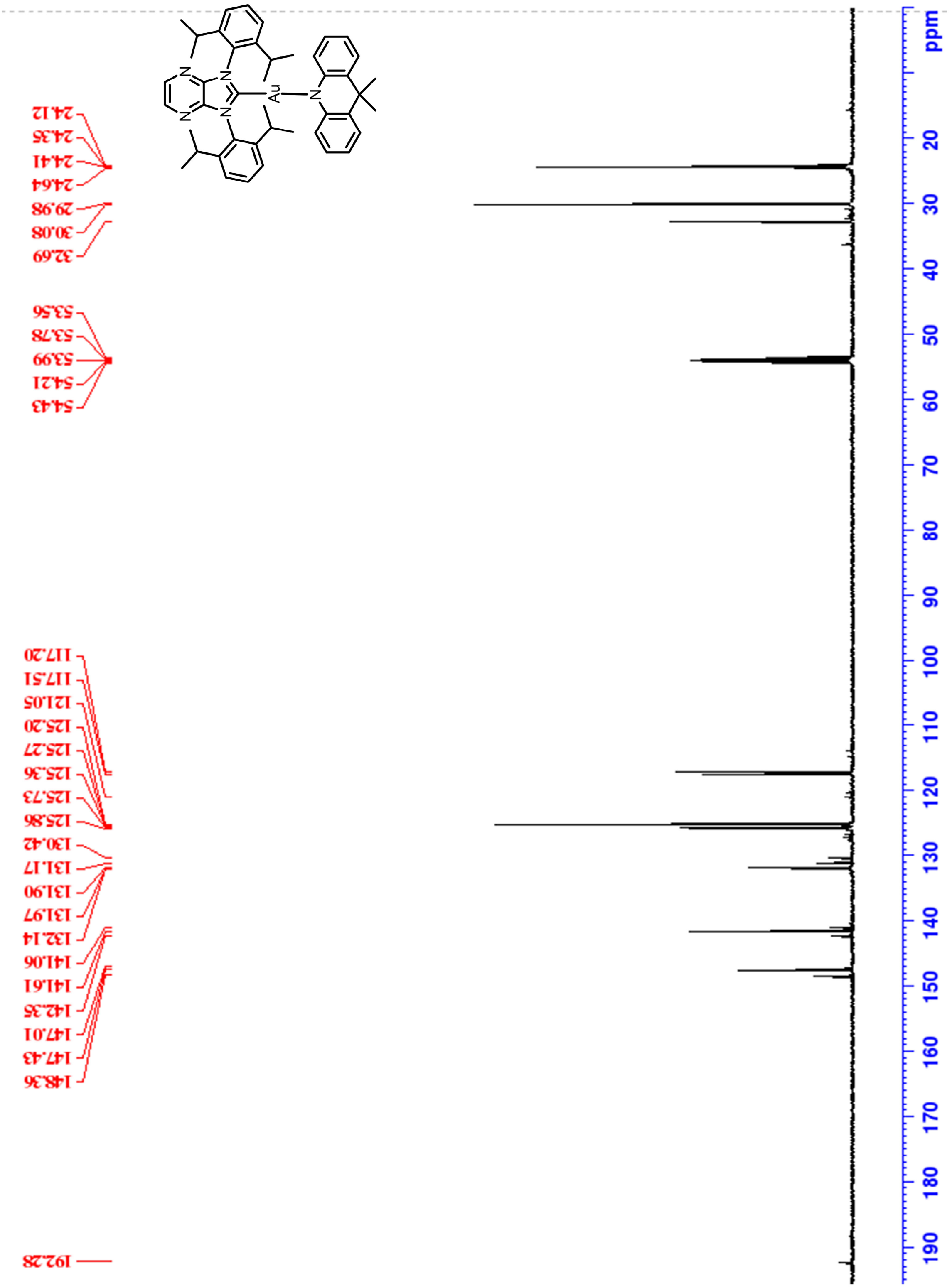

Figure S41. $^{13}C\{^{1}H\}$ NMR (126 MHz, CD_2Cl_2) spectrum of [Au(^{Dipp}PZI)(DMAC)].

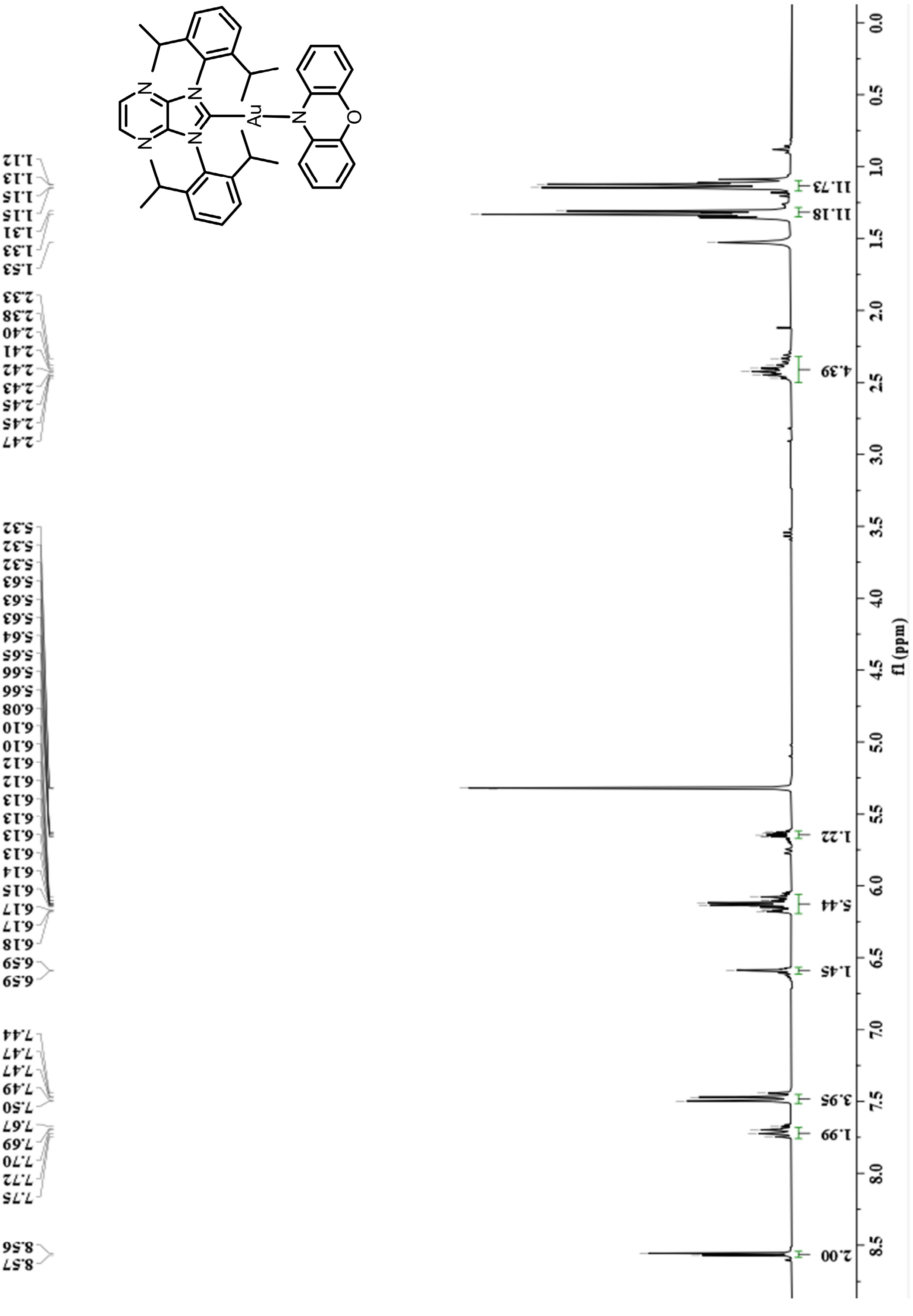

Figure S42. ^{1}H NMR (300 MHz, CD_2Cl_2) spectrum of [Au(^{Dipp}PZI)(PXZ)].

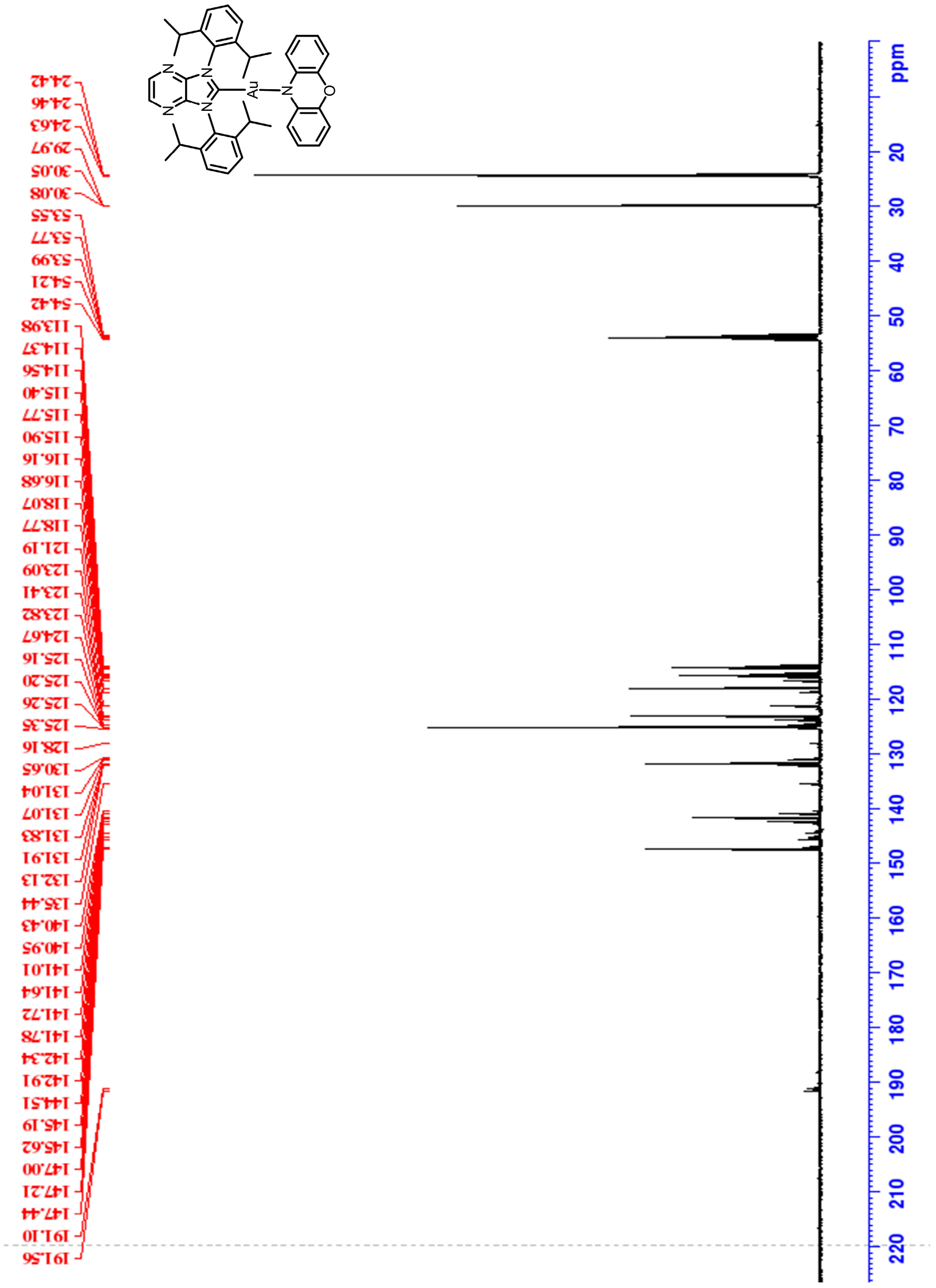

Figure S43. $^{13}C\{^{1}H\}$ NMR (126 MHz, CD_2Cl_2) spectrum of [Au(^{Dipp}PZI)(PXZ)].

References

[1] R. Hamze, M. Idris, D. S. M. Ravinson, M. C. Jung, R. Haiges, P. I. Djurovich, M. E. Thompson, *Front. Chem.* **2020**, 8, 401.
[2] C. N. Muniz, J. Schaab, A. Razgoniaev, P. I. Djurovich, M. E. Thompson, *J. Am. Chem. Soc.* **2022**, 144, 17916.
[3] G. M. Sheldrick, *Acta Cryst. A* **2008**, 64, 112.
[4] G. Heinrich, S. Schoof, H. Gusten, *J. Photochem.* **1974**, 3, 315.
[5] T. Hofbeck, U. Monkowius, H. Yersin, *J. Am. Chem. Soc.* **2015**, 137, 399.
[6] A. Ying, Y.-H. Huang, C.-H. Lu, Z. Chen, W.-K. Lee, X. Zeng, T. Chen, X. Cao, C.-C. Wu, S. Gong, C. Yang, *ACS Appl. Mater. Interfaces* **2021**, 13, 13478.
[7] T. Lu, F. Chen, *J. Comput. Chem.* **2011**, 33, 580.
[8] Z. Liu, T. Lu, Q. Chen, *Carbon* **2020**, 165, 461.
[9] Y. Li, L. Yang, H. He, L. Sun, H. Wang, X. Fang, Y. Zhao, D. Zheng, Y. Qi, Z. Li, W. Deng, *Nat. Commun.* **2022**, 13, 1355.
[10] S. Jin, X. Sui, G. C. Haug, V. D. Nguyen, H. T. Dang, H. D. Arman, O. V. Larionov, *ACS Catal.* **2022**, 12, 285.
[11] W. Song, J. Y. Lee, *Org. Electron.* **2017**, 51, 1.
[12] R. Hamze, S. Shi, S. C. Kapper, D. S. M. Ravinson, L. Estergreen, M.-C. Jung, A. C. Tadel, R. Haiges, P. I. Djurovich, J. L. Peltier, R. Jazzar, G. Bertrand, S. E. Bradforth, and M. E. Thompson, *J. Am. Chem. Soc.* **2019**, *141*, 8616.
[13] X. Feng, J.-G. Yang, J. Miao, C. Zhong, X. Yin, N. Li, C. Wu, Q. Zhang, Y. Chen, K. Li, C. Yang, *Angew. Chem., Int. Ed.* **2022**, *61*, e202209451.
